# The KWW Relaxation Spectrum Determination Using the Post–Widder Inversion Formula

**DOI:** 10.3390/ma19153256

**Published:** 2026-08-01

**Authors:** Anna Stankiewicz

**Affiliations:** Department of Technology Fundamentals, Faculty of Production Engineering, University of Life Sciences in Lublin, 20-612 Lublin, Poland; anna.m.stankiewicz@gmail.com

**Keywords:** viscoelasticity, relaxation time spectrum, linear relaxation modulus, Post–Widder inverse formula, Kohlrausch–Williams–Watts (KWW) model

## Abstract

**Highlights:**

- An analytical formula describing the KWW spectrum model directly in terms of the KWW modulus for an arbitrarily high model order was derived.
- It is shown that the differentiation of the relaxation modulus, inherent in the Post–Widder rule, is not needed here.
- The KWW spectrum approximate model is expressed as the product of the modulus and a finite series of the relaxation time.
- The algorithm ensures fast convergence of the generated model sequence, providing an arbitrarily accurate approximation of the KWW spectrum.
- A simple, adaptive rule for selecting subsequent model orders significantly reduces the number of iterations.

**Abstract:**

The problem of recovering the relaxation spectrum of the process described by the stretched exponential Kohlrausch–Williams–Watts (KWW) model is considered using the Post–Widder Laplace transform inversion rule. Based on the specific properties of the stretched exponential function, an analytical formula was derived that describes an arbitrarily high-order Post–Widder approximation of the spectrum directly in terms of the relaxation modulus, without any—neither analytical nor numerical—differentiation of the modulus as a product of finite power series of the relaxation times and the relaxation modulus. An alternative recurrence formula defining a sequence of the Post–Widder approximate models of the relaxation spectrum was developed. The positive definiteness, multiple differentiability, and zero asymptotic properties of the spectrum model are demonstrated; its extreme properties are discussed, and a sensitivity analysis with respect to the model parameters is conducted. The developed algorithm ensures fast convergence of the generated model sequence by applying a simple adaptive rule to select subsequent model orders, which relates them to the discrepancy between successive models and results in high-order models in at most a dozen iterations. Detailed numerical studies performed for nine values of the stretching exponent for KWW spectra covering relaxation times from 3 to 41 decades show that the proposed approach allows for generating an excellent approximation of the KWW spectrum by using only the relaxation modulus values in simple algebraic calculations.

## 1. Introduction

The history of the Kohlrausch–Williams–Watts (KWW) equation dates back to 1854 [1] when Rudolf Kohlrausch proposed the stretched exponential function to describe the relaxation of charge from a glass Leiden jar. Next, Friedrich Kohlrausch expanded the scope of the stretched exponential applicability to describing mechanical relaxations in the natural polymer silk in 1863 [2]. The stretched exponential relaxation model came to be referred to as the Kohlrausch–Williams–Watts model only after 1970, when Williams and Watts used it to describe non-Maxwellian dielectric relaxation [3]. According to Elton [4], the term “stretched exponential model”, currently identified with the KWW model, does not appear in the literature until 1984. Among the papers devoted to the history of the KWW model, articles [4,5,6,7] deserve special attention.

Modernly, stretched exponential models are used to describe slow dielectric and mechanical relaxation processes in many complex systems like glasses [8,9], glass-forming systems [10], ion-conducting glasses [11], pure glass-forming liquids and binary mixtures [12], simple van der Waals glass-formers [13], chalcogenide glasses [14], polymers [15,16], polymer melts [17], reprocessable cross-linked polymers [18], and thermoplastic and thermosetting polymers [19]. The scope of the KWW model’s applicability also includes new classes of polymers, e.g., vitrimers [20,21] and bone and bone collagen [22]. In the field of geophysics, the KWW model is used for modeling the decay rate of aftershocks [23] and time-domain-induced polarization data [24]. Recently, various researchers have effectively modeled the dielectric modulus using the KWW function, e.g., in [25,26]. The KWW equation is also applied for the mathematical description of a luminescence decay curve [27,28,29].

Combined and modified KWW models have also been used to obtain accurate predictions of relaxation processes. For example, the combined Maxwell–KWW model was applied by Triolo et al. [30] to describe the MD-derived hydrogen bonding relaxation dynamics and, in turn, by Sasaki and Enyo [22] to describe the stress relaxation of bovine femur. Multimode KWW equations were used for modeling relaxation processes in the polyester-based PU vitrimer (V-PU) by Farshbaf and Aghjeh [21] and in bovine femoral cortical bone specimens treated with KOH solution by Shirakawa et al. [31]. Bergmann [32] used a modified KWW model to describe dielectric relaxation processes in glass-forming materials, while Thakur et al. [26] applied a modified KWW model to describe the relaxation process hindering electrode polarization of polyvinylidene fluoride (PVDF) and its nanocomposites filled with MoS2 and h-BN as 2D nanofillers.

The wide scope of applications of the KWW model, established by hundreds of experimental works by dozens of researchers over many decades, has implied analytical studies devoted to this mathematical formalism, among which fundamental papers by Pollard [33], Mikusiński [34], Anderssen et al. [7], Montroll and Bendler [35], and Berberan-Santos [28] must be recalled.

This paper deals with the problem of determining the relaxation spectrum of a material whose relaxation process is described by the KWW model. In rheology, the relaxation time spectrum is a basic characteristic that provides a deep insight into the viscoelastic complex behavior of a material and allows for the unambiguous determination of its other rheological functions [36]. Knowledge of the relaxation spectrum is valuable for describing, analyzing, comparing, and improving the mechanical properties of materials [37]. Although the KWW relaxation modulus has a concise stretched exponential form, the related KWW relaxation spectrum is given by an infinite power series of the relaxation time [7,38,39]. Only for a few stretching exponents are analytical formulas known to describe the spectrum; generally, the series representation must be applied for the spectrum determination, as demonstrated in [40] for polyisoprene networks with the KWW stretching exponent 0.4. However, this series, although convergent, is generally very inconvenient due to its polynomial-type form. The KWW spectrum also has alternative integral representations [33,34], but their practical usefulness is also questioned [38].

Many methods and algorithms for determining relaxation spectra have been proposed under the assumption that the time [41,42,43,44,45,46,47,48] and frequency [49,50,51,52,53] domain characteristics or measurement data are known. These methods, inspired by various techniques of mathematical modeling and system identification, use a variety of analytical and numerical tools. Different reviews of relaxation spectra models and algorithms for their determination, carried out according to various criteria, can be found in many articles, for example in [46,51,54], and recently in [47,48].

Although there are many known methods of the relaxation spectrum recovery from the data available in the time domain, these methods are usually general and neither the classes of models used to describe the spectrum nor the spectra determination procedures themselves take into account the specificity of the stretched exponential relaxation. Moreover, the stretched exponential nature of the relaxation process may exacerbate the ill-posedness of the numerical optimization problems involving the best fit of the data and models. Although the results concerning the probability density of Lévy stable distribution given by stretched exponential function [55] can be applied here, the series form of the spectrum model composed with the products of powers of time and hypergeometric functions, while mathematically elegant, is not very convenient for further applications. Therefore, it is still desirable to develop a method for determining the relaxation spectrum model of the KWW relaxation process. The aim of this paper was to develop such a method, dedicated to stretched exponential relaxation, which will ensure computational and analytical convenience and simplicity of the obtained KWW spectrum model. It is assumed that for the considered material the KWW model describing the relaxation modulus is known.

The relaxation spectrum, not directly accessible by measurement, is uniquely determined by the inverse Laplace transform of the relaxation modulus given on the positive real axis. The basic inverse Laplace rule of this kind is the Post–Widder formula [56], which approximates the spectrum using the values of the modulus and its derivatives of high order. Therefore, the oldest class of methods for determining relaxation spectra uses the Post–Widder formula; the first methods were developed at the turn of the 1940s and 1950s [41,42,57,58]. The differential model proposed by Alfrey and Doty [57] resulted from the first-order Post–Widder rule. Kytopoulos et al. [15] applied the Alfrey and Doty formula to determine the KWW spectrum of solid polymers, while Zatryb et al. [29] used this formula studying stretched exponential photoluminescence decay for silicon nanocrystals. The simple spectrum model proposed by Ter Haar [58], given by the modulus multiplied by the inverse of the relaxation frequency, can be treated as an implementation of the zero-order Post–Widder rule. Ferry and Williams [59] also used the first-order Post–Widder differential formula; however, their method is, in fact, a second-order method since it requires taking the second derivative of the relaxation modulus. The relaxation spectrum approximation of the second-order was introduced by Schwarzl and Staverman [60]. Only twenty years later Tschoegl [61] used a third-order approximate spectrum model based on the Post–Widder formula. Stanislav and Hlaváček [62] discuss the numerical aspects and difficulties of realizing second- and third-order approximations of the relaxation spectrum using the Schwarzl and Staverman and Tschoegl methods, conducting their studies for the Rouse distribution of relaxation times. With the increasing order of approximation, the numerical inconveniences intensify.

In the above methods, differentiation is applied directly to the material’s relaxation modulus. In the next methods, following a two-stage approach, the relaxation modulus data are first approximated using multiple differentiable model, and then the Post–Widder formula is used for the determined model. Zi and Bažant [43] modeled the relaxation modulus using the Prony exponential series and decided the third-order Post–Widder rule to be sufficient to approximate the relaxation spectra of concrete. Cho et al. [47] used the Post–Widder inversion rule to compute an arbitrarily high-order relaxation time spectrum model based on a double-logarithmic-power-series modulus model and the author’s application of the cubic B-splines, applied both to find the optimal relaxation modulus model and to the numerical calculation of the high-order derivatives of this model. Recently, Stankiewicz [63], using specific properties of the double-logarithmic-power-series model, proved that the Post–Widder approximate spectrum model of arbitrarily high order is analytically described by a series of recurrence type directly related to the relaxation modulus model and a certain power series of the logarithm of time, without the need of the numerical differentiation.

In this paper, the Post–Widder inversion formula is applied to determine an approximate model of the KWW relaxation spectrum. The main results concern designation of the analytical formulas describing the n-th order Post–Widder approximate inversion of the stretched exponential relaxation modulus for the relaxation time spectrum modeling. First, analytical formulas, given by a series of recursive type, describing the n-th order derivative of the KWW modulus and approximate Post–Widder models of the KWW relaxation spectrum for arbitrarily high order were developed. Then, an analytical formula is derived that describes the relaxation spectrum model directly in terms of the relaxation modulus. This formula, valid for any high-order Post–Widder approximation, allows one to determine the relaxation spectrum model without the need to calculate the derivatives of the relaxation modulus. The monotonicity and asymptotic properties of the positive definite and multiple-differentiable relaxation spectrum model are examined and their extreme properties are outlined. Finally, computational determination of the KWW relaxation spectrum model is discussed and a complete computational algorithm is presented. The behavior of the KWW spectrum model in the presence of non-zero, bounded perturbations is investigated; analytical and numerical analysis is conducted and asymptotic properties of the perturbed model are studied. Extensive numerical studies have been carried out, both for the stretching exponents for which the analytical description of the spectrum is known, i.e., 1/3, 1/2 and 2/3, and for six additional exponents. To summarize the preliminary numerical studies, during which approximate models were determined for subsequent integer orders of the model, an adaptive simple rule for selecting successive orders of models iterated by the algorithm is derived that reduces the number of the algorithm’s iterations. Then, the effectiveness of the algorithm is verified and the quality of the generated models is assessed for nine stretching exponents. Concluding the considerations, it has been found that the proposed approach allows for the generation of an excellent approximation of the real KWW spectrum using only the values of the relaxation modulus, without any, either analytical or numerical, modulus differentiation. This means that only algebraic calculations are necessary to determine the KWW spectrum model for a given relaxation time.

Some of the tables and figures are moved to Supplementary Materials. Appendix A gives the proofs of the theorems and discusses the details of the integral coefficients of the new analytical formula describing the high-order Post–Widder approximation of the relaxation spectrum. The main symbols and abbreviations are summarized in Appendix B; wherever possible, units of physical quantities and references to the equation in which the quantity is defined or first appears are given.

## 2. Materials and Methods

In this section the Post–Widder inverse formula is applied to the KWW relaxation modulus model. First, the KWW relaxation model and the Post–Widder differential formula for the inversion of the Laplace transform are recalled. Then, the main results are formulated and proved. An analytical formula, given by a series of recursive type, describing the n-th order derivative of the relaxation modulus model is derived. Then, a recurrence formula defining a sequence of the Post–Widder approximate models of the relaxation spectrum is developed for an arbitrarily high order.

### 2.1. KWW Stretched Relaxation

The KWW model of the stretched (also called extended [20]) exponential relaxation is described by the relaxation modulus function [5,6,7,8]:

(1) $$G\left(t\right)=G_{0}e^{-{\left(\frac{t}{\tau_{r}}\right)}^{\beta }},$$

where $G\left(t\right)$ is a linear relaxation modulus, i.e., the stress induced in the material when the unit step strain is imposed, $G_{0}$ denotes the initial shear modulus, $\tau_{r}$ is the relaxation time, and 0<β<1 denotes the stretching exponent.

It is known that $G\left(t\right)$ (1) is a completely monotonic function [7,64,65], i.e., its subsequent derivatives are alternately a negative or positive definite as described for non-negative integers n and t>0 by the condition

(2) $${\left(-1\right)}^{n}\frac{d^{n}G\left(t\right)}{dt^{n}}>0,$$

which, based on the fundamental Hausdorff–Bernstain–Widder theorem on the completely monotonic functions [56,65], implies the spectral representation of $G\left(t\right)$ according to [7,56,65]:

(3) $$G\left(t\right)=\int_{0}^{\infty }\frac{H\left(\tau \right)}{\tau }e^{-t/\tau }d\tau ,$$

where $H\left(\tau \right)$ is a unique positive definite continuous spectrum that characterizes distribution of relaxation times τ.

The uni-modal relaxation time spectrum $H\left(\tau \right)$ of the KWW model, defined by the integral Equation (3), is described by the infinite series [7,38,39]:

(4) $$H\left(\tau \right)=\frac{G_{0}}{\pi }\;\sum_{k=1}^{\infty }\frac{{\left(-1\right)}^{k+1}}{k!}\sin\left(\pi \beta k\right)\;\Gamma \left(\beta k+1\right)\;{\left(\frac{\tau }{\tau_{r}}\right)}^{\beta k},$$

derived by Humbert [66] and, next, rigorously justified by Pollard [33], where $\Gamma \left(n\right)$ is Euler’s gamma function [64].

The KWW relaxation spectrum has also integral representation which follows directly from Pollard’s studies [33] and, using this paper’s notation, is as follows

(5) $$H\left(\tau \right)=\frac{G_{0}}{\pi }\frac{\tau_{r}}{\tau }\overset{\infty }{\underset{0}{\int }}e^{-\frac{\tau_{r}}{\tau }\;x}e^{-x^{\beta }\cos\left(\pi \beta \right)}sin\left[x^{\beta }\sin\left(\pi \beta \right)\right]dx.$$

Berberan-Santos [28] presented three alternative integral representations of $H\left(\tau \right)$. Another integral formula was given by Mikusiński [34], where the integration of some positive integrand is over the interval from 0 to π.

For some values of the stretching exponent the relaxation spectrum can be written in an analytical compact form. For $\beta =\frac{1}{2}$, from the known Laplace inverse transform of $e^{-\sqrt{t}}$ [67], the explicit formula follows [7,35]

(6) $$H\left(\tau \right)=\frac{G_{0}}{2\sqrt{\pi }}\sqrt{\frac{\tau }{\tau_{r}}}\;e^{-\frac{\tau }{4\tau_{r}\;}}.$$

Based on the results of Montroll and Bendler [35] on the Lévy stable distributions, for $\beta =\frac{1}{3}$ the spectrum is described by

(7) $$H\left(\tau \right)=\frac{G_{0}}{3\pi }\sqrt{\frac{\tau }{\tau_{r}}}\;K_{1/3}\left(\frac{2}{3}\sqrt{\frac{\tau }{3\tau_{r}}}\right),$$

where $K_{k}\left(x\right)$ is the modified Bessel function of the second kind [68] and is positive for x>0 and real v according to the property A.1 in [69].

However, the first description of the KWW relaxation spectrum for $\beta =\frac{2}{3}$ in terms of the Whittaker function comes from a fundamental paper by Pollard [33]; a correct formula can be derived from the inverse Laplace transform recently clarified by Mainardi et al. [70] and it is as follows

(8) $$H\left(\tau \right)=G_{0}\sqrt{\frac{3}{\pi }\;}exp\left[-\frac{2}{27}{\left(\frac{\tau }{\tau_{r}\;}\right)}^{2}\right]\;W_{1/2,1/6}\left(\frac{4}{27}{\left(\frac{\tau }{\tau_{r}\;}\right)}^{2}\right),$$

where $W_{u,v}\left(x\right)$ denotes the Whittaker function, defined via the confluent hypergeometric functions [71,72], which for the indices u=1/2 and v=1/6 takes complex values on the negative real axis and is a positive definite for non-negative real arguments.

The reader is referred to [7] for a more detailed historical perspective.

For other values of β, practical applications of the spectrum $H\left(\tau \right)$ require replacing the infinite series in (4) with a finite one. The series described by Equation (4), however convergent, is inconvenient both for analysis and for the use in determining other rheological characteristics. Lindsey and Patterson [38] have pointed out difficulties in calculating the spectrum using the series representation according to (4), especially for $\tau >\tau_{r}$. They found the integral formula in (5) to be more convenient for spectrum calculation. The numerical difficulties associated with the use of infinite power series were also pointed out by Wu and Jia [39]. The numerical problems can be eliminated if the Post–Widder Laplace inversion rule is applied to find the model of the KWW spectrum.

### 2.2. Post–Widder Approximation of the Relaxation Spectrum

The integral in Equation (3) can be equivalently expressed as

$$G\left(t\right)=\int_{0}^{\infty }\frac{H\left(\frac{1}{v}\right)}{v}e^{-tv}dv,$$

which means that the modulus $G\left(t\right)$ is directly the Laplace integral of $H\left(1/v\right)/v$. Since $G\left(t\right)$ is known on the positive real axis, the Post–Widder inversion formula [56] holds, which, written in terms of the relaxation times τ>0, is as follows [63]

(9) $$H\left(\tau \right)=\underset{n\to \infty }{\lim}{\left(-1\right)}^{n}\frac{1}{\left(n-1\right)!}{\left(n\tau \right)}^{n}{\left.\frac{d^{n}G\left(t\right)}{dt^{n}}\right|}_{t=n\tau },$$

whence the approximated model

(10) $$H_{n}\left(\tau \right)={\left(-1\right)}^{n}\frac{1}{\left(n-1\right)!}{\left(n\tau \right)}^{n}{\left.\frac{d^{n}G\left(t\right)}{dt^{n}}\right|}_{t=n\tau },$$

results for n≥1 when the limit is dropped, being the n-th order Post–Widder approximate formula; the subscript denotes the order of the spectrum $H\left(\tau \right)$ approximation.

Some known relaxation time spectrum approximations are special cases of the formula (10). For n=1, we obtain

(11) $$H_{1}\left(\tau \right)=-\tau {\left.\frac{dG\left(t\right)}{dt}\right|}_{t=\tau },$$

which is the differential model developed by Alfrey and Doty [57]. If n=2, then the approximate model takes the form

(12) $$H_{2}\left(\tau \right)={\left(2\tau \right)}^{2}{\left.\frac{d^{2}G\left(t\right)}{dt^{2}}\right|}_{t=2\tau },$$

which is the Schwarzl and Staverman [60] second order relaxation spectrum approximation. In turn, the simple model proposed by Ter Haar [58]

(13) $$H_{0}\left(\tau \right)=G\left(\tau \right),$$

can be treated as a zero-order Post–Widder approximate model.

The above models used low-order approximations, from zero to third order by Tschoegl [61], whence the quality of the real relaxation spectrum approximation was unsatisfactory, which for the KWW model is perfectly illustrated by the examples given below. However, by choosing a sufficiently high order of approximation, excellent spectrum accuracy can be obtained, especially when the numerical differentiation of the relaxation modulus is replaced by the calculation of derivatives using the analytical formulas. Such formulas, describing derivatives of arbitrarily high order, are derived in the next section.

### 2.3. Application of the Post–Widder Formula to the KWW Model

The complete monotonic KWW relaxation modulus has infinitely many derivatives of integer order. The goal of this section is to determine the analytical formula describing the n-th order Post–Widder approximation $H_{n}\left(\tau \right)$ given by (10) of the KWW relaxation spectrum. First, the derivative $\frac{d^{n}G\left(t\right)}{dt^{n}}$ is expressed by a recurrence-type formula; next, the respective result is given for the approximate spectrum model $H_{n}\left(\tau \right)$.

By (1), for the first derivative with respect to the time we have

(14) $$\frac{dG\left(t\right)}{dt}=-\frac{\beta }{\tau_{r}}G\left(t\right){\left(\frac{t}{\tau_{r}}\right)}^{\beta -1}.$$

Generally, a derivative $\frac{d^{n}G\left(t\right)}{dt^{n}}$ is described by the analytical formula of recursive-type series specified by the following result proved in Appendix A.1.

**Theorem** **1.** *Let* n≥1 *and* t>0 *. Then, the* n*-th order derivative of the KWW relaxation modulus* $G\left(t\right)$ *expressed by Equation (1) is given by*

(15) $$\frac{d^{n}G\left(t\right)}{dt^{n}}=-\tau_{r}^{-\beta }\sum_{k=1}^{n}\left(\begin{array}{c} n-1\\ k-1 \end{array}\right){\left(\beta \right)}_{k}\frac{d^{n-k}G\left(t\right)}{dt^{n-k}}t^{\beta -k},$$

*where* $\left(\begin{array}{c} n\\ k \end{array}\right)$ *denotes the binomial coefficient and* ${\left(p\right)}_{i}$ *is the failing factorial defined as follows* [73]

(16) $${\left(p\right)}_{i}=p\left(p-1\right)\left(p-2\right)\left(p-3\right)\;\ldots \;\left(p-i+1\right)=\prod_{m=0}^{i-1}\left(p-m\right).$$

The result is proved by induction; however, it can also be obtained by the general Leibniz rule for higher-order derivatives of the product of two functions applied to the first derivative (14). The binomial coefficients $\left(\begin{array}{c} n-1\\ k-1 \end{array}\right)$ are positive for any n and k, while the sign of the failing factorial ${\left(\beta \right)}_{k}$ for 0<β<1 depends on k and is characterized by inequality

(17) $${\left(-1\right)}^{k+1}{\left(\beta \right)}_{k}>0,$$

which means that ${\left(\beta \right)}_{k}$ are positive for odd k and are negative whenever k is even.

From the inspection of the Post–Widder n-th order relaxation spectrum approximation in (10), we see that the product $t^{n}\frac{d^{n}G\left(t\right)}{dt^{n}}$, not the derivative $\frac{d^{n}G\left(t\right)}{dt^{n}}$ itself, is fundamental here. Multiplying both sides of (15) by $t^{n}$ we obtain

(18) $$t^{n}\frac{d^{n}G\left(t\right)}{dt^{n}}=-{\left(\frac{t}{\tau_{r}}\right)}^{\beta }\sum_{k=1}^{n}\left(\begin{array}{c} n-1\\ k-1 \end{array}\right){\left(\beta \right)}_{k}\;t^{n-k}\frac{d^{n-k}G\left(t\right)}{dt^{n-k}},$$

from which, having in mind Equation (10), the next result is easily derived.

**Theorem** **2.** *Let* n≥1 *and* τ>0*. Then, the* n*-th order Post–Widder approximation* $H_{n}\left(\tau \right)$ *given by Equation (10) of the KWW relaxation spectrum is described by the recurrence type formula*

(19) $$H_{n}\left(\tau \right)={\left(\frac{n\tau }{\tau_{r}}\right)}^{\beta }\sum_{k=1}^{n-1}\frac{{\left(\beta \right)}_{k}}{\left(k-1\right)!\left(n-k\right)}{\left(-1\right)}^{k+1}H_{n-k}\left(\frac{n}{n-k}\tau \right)+\frac{{\left(-1\right)}^{n+1}}{\left(n-1\right)!}{\left(\frac{n\tau }{\tau_{r}}\right)}^{\beta }{\left(\beta \right)}_{n}G\left(n\tau \right).$$

Including the notation introduced in (13), the above formula can be written as

(20) $$H_{n}\left(\tau \right)={\left(\frac{n\tau }{\tau_{r}}\right)}^{\beta }\left[\sum_{k=1}^{n-1}\frac{{\left(\beta \right)}_{k}}{\left(k-1\right)!\left(n-k\right)}{\left(-1\right)}^{k+1}H_{n-k}\left(\frac{n}{n-k}\tau \right)+\frac{{\left(-1\right)}^{n+1}}{\left(n-1\right)!}{\left(\beta \right)}_{n}H_{0}\left(n\tau \right)\right].$$

Thus, the n-th order spectrum approximation is a linear combination of all the preceding approximations $H_{n-k}\left(\cdot \right)$ for the shifted arguments $\frac{n}{n-k}\tau$ (compressed with the scaling factor $\frac{n}{n-k}>1$) and $H_{0}\left(n\tau \right)$ jointly multiplied by ${\left(\frac{n\tau }{\tau_{r}}\right)}^{\beta }$, in which, in view of the property described by Equation (17), all the coefficients are positive.

Let us consider the first Post–Widder spectrum approximations $H_{n}\left(\tau \right)$. For n=1, Equation (19) takes the form

(21) $$H_{1}\left(\tau \right)=\beta {\left(\frac{\tau }{\tau_{r}}\right)}^{\beta }G\left(\tau \right),$$

being, in view of (14), identical with the result from the Alfrey and Doty rule (11). For n=2, Equation (20) is as follows

$$H_{2}\left(\tau \right)=\beta {\left(\frac{2\tau }{\tau_{r}}\right)}^{\beta }\left[H_{1}\left(2\tau \right)+\left(1-\beta \right)H_{0}\left(2\tau \right)\right],$$

and, by (21), it takes the form

(22) $$H_{2}\left(\tau \right)=\beta \left[\beta {\left(\frac{2\tau }{\tau_{r}}\right)}^{\beta }-\left(\beta -1\right)\right]{\left(\frac{2\tau }{\tau_{r}}\right)}^{\beta }G\left(2\tau \right).$$

Thus, on the basis of (14), model $H_{2}\left(\tau \right)$ coincides with the model obtained by Schwarzl and Staverman as Equation (12).

In view of (19), the third order spectrum approximation takes the form

$$H_{3}\left(\tau \right)=\frac{\beta }{2}\left[H_{2}\left(\frac{3}{2}\tau \right)-2\left(\beta -1\right)H_{1}\left(3\tau \right)+\left(\beta -1\right)\left(\beta -2\right)G\left(3\tau \right)\right]{\left(\frac{3\tau }{\tau_{r}}\right)}^{\beta }.$$

Whence, taking into account the previous approximations $H_{2}\left(\tau \right)$ and $H_{1}\left(\tau \right)$ given by (22) and (21), we can express $H_{3}\left(\tau \right)$ directly in terms of the relaxation modulus $G\left(3\tau \right)$ for triple relaxation time τ as follows

(23) $$H_{3}\left(\tau \right)=\frac{\beta }{2}\left[\beta^{2}{\left(\frac{3\tau }{\tau_{r}}\right)}^{2\beta }-3\beta \left(\beta -1\right){\left(\frac{3\tau }{\tau_{r}}\right)}^{\beta }+\left(\beta -1\right)\left(\beta -2\right)\right]{\left(\frac{3\tau }{\tau_{r}}\right)}^{\beta }G\left(3\tau \right).$$

The formulas described in Equations (21)–(23) suggest that in the case of the KWW model the Post–Widder approximation of the relaxation time spectrum can be expressed directly in terms of the relaxation modulus, without the need to calculate its derivatives. It the next section, such formulas describing the modulus derivatives and the models $H_{n}\left(\tau \right)$ of an arbitrarily high orders are derived and a computational algorithm resulting in an approximate spectrum model is presented.

## 3. Results

In this section an algebraic formula describing model $H_{n}\left(\tau \right)$ directly in terms of the relaxation modulus $G\left(\tau \right)$ is derived. The monotonicity and asymptotic properties of the positive definite and multiple-differentiable relaxation spectrum model $H_{n}\left(\tau \right)$ are examined and their extreme properties are discussed. A short background of the algebraic computations is given. Then, two model quality indices are introduced. Computational determination of the Post–Widder relaxation spectrum approximate model is discussed and a complete computational algorithm is presented. The behavior of the KWW spectrum model in the presence of non-zero, bounded perturbations is investigated; deterministic analysis is conducted and asymptotic properties of the perturbed model are studied.

### 3.1. The Main Formulas

The formulas (21)–(23) suggest that, in the case of the KWW relaxation, the Post–Widder approximation of the relaxation spectrum can be expressed directly in terms of the modulus, without the need to determine its derivatives. To specify this result, first the next theorem is proved in Appendix A.2.

**Theorem** **3.** *Let* n≥1 *and* t>0*. Then, the* n*-th order derivative of the KWW relaxation modulus* $G\left(t\right)$ *given by Equation (1) is as follows*

(24) $$\frac{d^{n}G\left(t\right)}{dt^{n}}={\left(-1\right)}^{n}{\left(\frac{1}{\tau_{r}}\right)}^{n}\left[\sum_{k=1}^{n}a\left(n,k;\beta \right){\left(\frac{t}{\tau_{r}}\right)}^{-\left(k-1\right)\beta }\right]{\left(\frac{t}{\tau_{r}}\right)}^{n\left(\beta -1\right)}G\left(t\right),$$

*where the coefficients* $a\left(n,k;\beta \right)$*, being the functions of two integer arguments* n≥1*,* 1≤k≤n *and dependent on* β*, are defined by the recurrence:*

(25) $$a\left(n+1,k;\beta \right)=\left[n-\beta \left(n-k+2\right)\right]a\left(n,k-1;\beta \right)+\beta a\left(n,k;\beta \right)$$

*for* 2≤k≤n *and the boundary conditions with respect to* k*:*

(26) $$a\left(n,1;\beta \right)=\beta^{n},$$

(27) $$a\left(n,n;\beta \right)={\left(-1\right)}^{n+1}{\left(\beta \right)}_{n}.$$

According to the above result, the n-th order derivative of the KWW modulus $G\left(t\right)$ can be expressed directly in terms of the modulus, without the derivatives of $G\left(t\right)$, as it was in Theorem 1. The specific stretched-exponential form of the KWW model was consequential here.

Although mathematical induction was applied to prove the above theorem, the derivative $d^{n}G\left(t\right)/dt^{n}$ can also be found by applying the Faà di Bruno’s formula [74,75], which is a generalization of the chain rule to higher-order derivatives, to the modulus $G\left(t\right)$ given by Equation (1), expressed as a composite function $G\left(t\right)=G_{0}e^{-x\left(t\right)}$, where $x\left(t\right)={\left(\frac{t}{\tau_{r}}\right)}^{\beta }$. However, the proof by mathematical induction directly uses the properties of the KWW model and allows us to avoid the very complicated additive–multiplicative form of the Faà di Bruno’s formula, in which the sum and powers of the successive derivatives of the internal function are taken over all n-triples of nonnegative integers satisfying some linear constraint.

Theorem 3 implies the following expression describing the Post–Widder approximate model $H_{n}\left(\tau \right)$ defined in (10) directly in terms of the KWW relaxation modulus $G\left(t\right)$.

**Theorem** **4.** *Let* n≥1 *and* τ>0*. Then, the* n*-th order Post–Widder approximate model* $H_{n}\left(\tau \right)$ *defined by Equation (10) of the KWW relaxation spectrum* $H\left(\tau \right)$ *is given by*

(28) $$H_{n}\left(\tau \right)=\frac{1}{\left(n-1\right)!}\left[\sum_{k=1}^{n}a\left(n,k;\beta \right){\left(\frac{n\tau }{\tau_{r}}\right)}^{-\left(k-1\right)\beta }\right]{\left(\frac{n\tau }{\tau_{r}}\right)}^{n\beta }G\left(n\tau \right),$$

*where* $G\left(n\tau \right)$ *is the KWW relaxation modulus for the time* t=nτ *and the coefficients* $a\left(n,k;\beta \right)$ *are defined by Equations (25)–(27).*

The following property, useful for further considerations, is proved in Appendix A.3.

**Proposition** **5.** *Let* n≥1 *and* 0<β<1*. Then, for any* 1≤k≤n *the coefficients* $a\left(n,k;\beta \right)$*, defined by Equations (25)–(27), are positive,* i.e.*,*

(29) $$a\left(n,k;\beta \right)>0.$$

Two compact formulas describing coefficients $a\left(n,k;\beta \right)$ for k=2,3 are derived in Supplementary Materials S1. The coefficients $a\left(n,k;\beta \right)$, in particular according to Equation (S2):

(30) $$a\left(n,2;\beta \right)=\frac{n\left(n-1\right)}{2}\left(1-\beta \right)\beta^{n-1}.$$

For fixed model order n, monotonicity of the coefficients $a\left(n,k;\beta \right)$ depends on the parameter β and is characterized by the next property proved in Appendix A.4.

**Proposition** **6.** *Let* n≥2 *and* 0<β<1*. Then, for fixed* n*, the sequence* $\left\{a\left(n,k;\beta \right)\right\}$ *of coefficients* $a\left(n,k;\beta \right)$ *defined by Equations (25)–(27):*

*(i)*    *For* $0<\beta <\frac{1}{2}$ *is monotonically (strictly) increasing for* 1≤k≤n*;*
*(ii)*   *For* $\beta =\frac{1}{2}$ *is monotonically (strictly) increasing for* 1≤k≤n−1 *and such that* $a\left(n,n-1;\beta \right)=a\left(n,n;\beta \right)$*;*
*(iii)*  *For* $\frac{1}{2}<\beta <\frac{3}{4}$ *is monotonically (strictly) increasing for* 1≤k≤n−1 *and such that* $a\left(n,n-1;\beta \right)>a\left(n,n;\beta \right)$*.*

For $\frac{3}{4}\leq \beta <1$, the monotonicity of the sequence $\left\{a\left(n,k;\beta \right)\right\}$ is more complicated: it can change, increase and decrease with the increase of k for a fixed n, especially for small model orders n. For example, for $\beta =\frac{3}{4}$ and n=3, according to (A8), we have

$$a\left(3,1;\beta \right)={\left(\frac{3}{4}\right)}^{3}=a\left(3,2;\beta \right)>a\left(3,3;\beta \right)=\frac{1}{4}\left(\frac{3}{4}\right)\left(\frac{5}{4}\right),$$

while for n=4, by formulas (26), (30), (S5), and (27) we obtain

$$a\left(4,1;\beta \right)={\left(\frac{3}{4}\right)}^{4}<a\left(4,2;\beta \right)=2{\left(\frac{3}{4}\right)}^{4}<a\left(4,3;\beta \right)=\left(\frac{1}{4}\right){\left(\frac{3}{4}\right)}^{2}\left(\frac{23}{4}\right)>\left(4,4;\beta \right)=\left(\frac{1}{4}\right)\left(\frac{3}{4}\right)\left(\frac{5}{4}\right)\left(\frac{9}{4}\right),$$

which means that the monotonicity described in case (*iii*) of Proposition 6 is true; in fact, it is true for n≥4. Two other examples are given in Appendix A.5, where for β=0.9 and β=0.95 the first six and eight rows and columns, respectively, of a two-dimensional lower triangular matrix composed of coefficients $a\left(n,k;\beta \right)$ are given. For β=0.9, a quick review of Table A1 shows that the sequence $\left\{a\left(n,k;\beta \right)\right\}$ is strictly decreasing for n=2,3,4, while for any fixed n≥5 it is strictly increasing for 1≤k≤n−1 and such that $a\left(n,n-1;\beta \right)>a\left(n,n;\beta \right)$. In turn, for β=0.95, the sequence $\left\{a\left(n,k;\beta \right)\right\}$ is also strictly decreasing for n=2,3,4; for n=5,6 it first decreases because $a\left(n,1;\beta \right)>a\left(n,2;\beta \right)$, then it increases for 2≤k≤n−1 and finally $a\left(n,n-1;\beta \right)>a\left(n,n;\beta \right)$; see Table A2. Only for n≥7 does the property described in the item (*iii*) of Proposition 6 hold. From a practical point of view, as confirmed by the numerical studies conducted and presented below, the Post–Widder approximations of high orders are of particular interest. Therefore, based on numerical studies, the following property was formulated.

**Property** **1.** *Let* $\frac{3}{4}\leq \beta <1$*. Then, for fixed model order* n *such that*

(31) $$n>\frac{1+\sqrt{\frac{1+7\beta }{1-\beta }}}{2},$$

 *the sequence* $\left\{a\left(n,k;\beta \right)\right\}$ *of coefficients* $a\left(n,k;\beta \right)$ *defined by Equations (25)–(27) is monotonically (strictly) increasing for* 1≤k≤n−1 *and such that* $a\left(n,n-1;\beta \right)>a\left(n,n;\beta \right)$.

The above result means that for $\beta =\frac{3}{4}$ this monotonicity property is satisfied for any n≥4. It is satisfied for any n≥4, also when $\frac{3}{4}\leq \beta <\frac{6}{7}$, for any n≥5 when $\frac{6}{7}\leq \beta <\frac{10}{11}$, and for any n≥6 whenever $\frac{10}{11}\leq \beta <\frac{15}{16}$. Generally, inequality (31) holds for an arbitrary $n\geq n_{min}$ when

$$\frac{\left(n_{min}-1\right)\left(n_{min}-2\right)}{\left(n_{min}-1\right)\left(n_{min}-2\right)+2}\leq \beta <\frac{n_{min}\left(n_{min}-1\right)}{n_{min}\left(n_{min}-1\right)+2}.$$

Other properties of the double-indexed coefficients $a\left(n,k;\beta \right)$ are detailed in Supplementary Materials S1. The coefficients $a\left(n,k;\beta \right)$.

The model $H_{n}\left(\tau \right)$ given by (28) can be expressed as a product of the n-th order polynomial of the argument ${\left(\frac{n\tau }{\tau_{r}}\right)}^{\beta }$ and the stretched exponential function $G\left(n\tau \right)$ as follows

(32) $$H_{n}\left(\tau \right)=\frac{1}{\left(n-1\right)!}\left[\sum_{k=1}^{n}a\left(n,k;\beta \right){\left(\frac{n\tau }{\tau_{r}}\right)}^{\left(n-k+1\right)\beta }\right]G\left(n\tau \right).$$

The convergence in the Post–Widder inversion formula (9) implies that

(33) $$H_{n}\left(\tau \right)\to H\left(\tau \right)$$

as n→∞ [56], which means that the sequence of the relaxation spectrum models $H_{n}\left(\tau \right)$ given by finite series in (32) converge to the relaxation time spectrum $H\left(\tau \right)$ given by infinite series in (4).

### 3.2. Monotonicity, Positivity and Asymptotic Properties of the Relaxation Spectrum Model

In view of the positivity of the coefficients $a\left(n,k;\beta \right)$, Equation (29), the n-th order Post–Widder approximate model $H_{n}\left(\tau \right)$ given by (28) is positive definite, i.e., $H_{n}\left(\tau \right)>0$, for any τ>0. The KWW relaxation modulus $G\left(t\right)$ is a multiple-differentiable function of t, whence also $H_{n}\left(\tau \right)$ is a multiple-differentiable function of τ. The asymptotic properties of $H_{n}\left(\tau \right)$ are stated by the following theorem proved in Appendix A.6.

**Theorem** **7.** *Let* n≥1 *and* τ>0*. Then, the* n*-th order Post–Widder approximate model* $H_{n}\left(\tau \right)$ *given by Equation (28) satisfies the following ‘zero’ asymptotic properties*

(34) $$\underset{\tau \to 0^{+}}{lim}\;H_{n}\left(\tau \right)=0\;\;\;\mathrm{and}\;\;\;\underset{\tau \to \infty }{lim}\;H_{n}\left(\tau \right)=0.$$

The next theorem asserts some properties of the maximum of the model $H_{n}\left(\tau \right)$; the outline of the proof is moved into Appendix A.7.

**Theorem** **8.** *Let* n≥1 *and* τ>0*. Then, the* n*-th order Post–Widder approximate model* $H_{n}\left(\tau \right)$ *given by Equation (28) has a maximum* $\tau_{n}^{*}$ *defined by*

(35) $$\underset{\tau >0}{\max}\;H_{n}\left(\tau \right)=H_{n}\left(\tau_{n}^{*}\right)$$

*such that* $\tau_{1}^{*}=\tau_{r}$ *for* n=1 *and*

(36) $$\tau_{r}n^{-1}<\tau_{n}^{*}<\tau_{r}n^{\frac{1}{\beta }-1}$$

*for* n>1*, which satisfies the stationary point equation of polynomial-type*

(37) $$-\beta a\left(n,1;\beta \right){\left(\frac{n\tau }{\tau_{r}}\right)}^{n\beta }+\sum_{k=1}^{n-1}\left[na\left(n,k;\beta \right)-a\left(n+1,k+1;\beta \right)\right]{\left(\frac{n\tau }{\tau_{r}}\right)}^{\left(n-k\right)\beta }+\beta a\left(n,n;\beta \right)=0.$$

By introducing an auxiliary variable $x={\left(\frac{n\tau }{\tau_{r}}\right)}^{\beta }$, Equation (37) can be rewritten as a standard polynomial equation

$$-\beta a\left(n,1;\beta \right)x^{n}+\sum_{k=1}^{n-1}\left[na\left(n,k;\beta \right)-a\left(n+1,k+1;\beta \right)\right]x^{n-k}+\beta a\left(n,n;\beta \right)=0,$$

where the free term $\beta a\left(n,n;\beta \right)$ is positive, the coefficient at the highest power is negative, and the signs of $\left[na\left(n,k;\beta \right)-a\left(n+1,k+1;\beta \right)\right]$ depend, in particular, on the value of β.

### 3.3. Short Background of Algebraic Computations

We will briefly discuss the computational issues of the formula (32). However, there exist direct algebraic formulas for the real positive coefficients $a\left(n,k;\beta \right)$; as detailed in Supplementary Materials, these coefficients can be conveniently computed directly from the recurrence two-summands formula (25). For computation of the falling factorial ${\left(\beta \right)}_{k}$ in (27), the gamma function $\Gamma \left(n\right)$ can be used according to the known expression

(38) $${\left(p\right)}_{i}=\frac{\Gamma \left(p+1\right)}{\Gamma \left(p-i+1\right)}.$$

Alternatively, the failing factorial identity (A4) can be applied.

For a given value of the model order n and stretching exponent β, the coefficients $a\left(n,k;\beta \right)$ constitute the n×n dimensional lower triangular matrix array $A_{n}\left(\beta \right)$, with the first column, according to (26), composed of the successive integer powers of β, and the main diagonal composed of the absolute value of ${\left(\beta \right)}_{n}$ in view of (27). If, for a given β, we set n→n+1, only n+1 additional coefficients $a\left(n+1,k;\beta \right)$ must be computed for 1≤k≤n+1, according to the recurrence rule (25).

### 3.4. Model Quality Indices

Suppose the KWW relaxation modulus $G\left(t\right)$ described by (1) is given, i.e., the parameters $G_{0}$, $\tau_{r}$ and β are known. Let $T=\left[\tau_{min},\tau_{max}\right]$ be the interval of the relaxation times applied for computation of the model $H_{n}\left(\tau \right)$ given by (32), where $\tau_{min}>0$ and $\tau_{max}<\infty$. Let $\tau_{j}\in T$ be the sampling times, j=1,… ,N, where N is the assumed number of the points at which the spectrum model $H_{n}\left(\tau \right)$ is sampled.

The determining of an accurate relaxation spectrum model consists of computing a $H_{n}\left(\tau \right)$ that provides a good approximation of the real spectrum $H\left(\tau \right)$ (4). The model accuracy index is as follows

(39) $$Q\left(n\right)=\frac{1}{N}\sum_{j=1}^{N}{\left[\frac{H_{n}\left(\tau_{j}\right)-H\left(\tau_{j}\right)}{H\left(\tau_{j}\right)}\right]}^{2}=\frac{1}{N}\sum_{j=1}^{N}{\left[1-\frac{H_{n}\left(\tau_{j}\right)}{H\left(\tau_{j}\right)}\right]}^{2}.$$

In view of (33), $Q\left(n\right)\to 0$, as n→∞. However, the real spectrum $H\left(\tau \right)$ is generally unknown, so the model quality index useful in calculations cannot be directly related to it. Therefore, to check whether the spectrum model $H_{n}\left(\tau \right)$ gives satisfactory approximation, the mean relative index of the discrepancy between successive models

(40) $$J\left(n\right)=\frac{1}{N}\sum_{j=1}^{N}{\left[\frac{H_{n}\left(\tau_{j}\right)-H_{n+1}\left(\tau_{j}\right)}{H_{n+1}\left(\tau_{j}\right)}\right]}^{2}=\frac{1}{N}\sum_{j=1}^{N}{\left[1-\frac{H_{n}\left(\tau_{j}\right)}{H_{n+1}\left(\tau_{j}\right)}\right]}^{2}$$

is used. Due to (33), $J\left(n\right)\to 0$ for n→∞ too. The relations between these indices are specified by the proposition proved in Appendix A.8.

**Proposition** **9.** *Let* n≥1, 0<β<1 *and the sampling times* $\tau_{j}\in T$*,* j=1,… ,N*. Then, for any* n *the inequalities hold:*

(41) $$J\left(n\right)\leq M_{n+1}^{2}Q\left(n\right)+M_{n+1}^{2}Q\left(n+1\right)+2M_{n+1}^{2}\sqrt{Q\left(n\right)Q\left(n+1\right)},$$

(42) $$Q\left(n\right)\leq \frac{1}{m_{n+1}^{2}}J\left(n\right)+2\left|\frac{1}{m_{n+1}^{2}}-\frac{1}{M_{n+1}}\right|\sqrt{J\left(n\right)}+\left(\frac{1}{m_{n+1}^{2}}+1-\frac{2}{M_{n+1}}\right),$$

*where positive constants* $m_{n+1}$ *and* $M_{n+1}$ *are such that the following lower and upper bounds hold for any* τ∈T

(43) $$m_{n+1}\leq \frac{H\left(\tau \right)}{H_{n+1}\left(\tau \right)}\leq M_{n+1}.$$

The existence of $m_{n+1}$ and $M_{n+1}$ is demonstrated in the proposition’s proof. When $Q\left(n+1\right)\leq Q\left(n\right)$, then inequality (41) takes an especially simple form

(44) $$J\left(n\right)\leq 4M_{n+1}^{2}Q\left(n\right).$$

If the lower bound $m_{n+1}>1$, a less restrictive $m_{n+1}\leq 1$ can be taken. Then, the last term in the upper bound in (42) is nonnegative. In view of the convergence described by (33), the bounds $m_{n+1}\to 1$ and $M_{n+1}\to 1$ as n→∞. The inequalities (41), (42), and (44) mean that the speeds of the convergences of $Q\left(n\right)\to 0$ and $J\left(n\right)\to 0$, as n→∞, are of the same order. Both indices were computed during numerical studies when the real spectra were known. However, in the general case when the relaxation spectrum is unknown, only the index $J\left(n\right)$ can be used. Then, in view of inequality (42), a bounded $J\left(n\right)$ implies a bounded index $Q\left(n\right)$.

### 3.5. Determination of the Relaxation Spectrum Approximate Model

Let us assume that the KWW relaxation modulus $G\left(t\right)$ given by Equation (1) is known. The determining of the exact model of the relaxation spectrum involves computing such a $H_{n}\left(\tau \right)$, described by (32), that well approximates the real spectrum $H\left(\tau \right)$. Since $H\left(\tau \right)$ is unknown, only the successive model’s discrepancy index $J\left(n\right)$ (40) can be used during the computational process to check whether the model $H_{n}\left(\tau \right)$ is a satisfactory approximation to $H\left(\tau \right)$. The convergence expressed in (33) holds; however, the sequence of models $H_{n}\left(\tau \right)$ slowly converges to the real spectrum $H\left(\tau \right)$. This is a known property of the Post–Widder approximations [76], confirmed by the numerical studies presented below. Therefore, it is convenient to increase in the successive iterations the order of approximation n by more than one. Based on the results of the numerical studies carried out for the coefficients β, for which the relaxation spectra are described by the known concise analytical formulas (6)–(8), an empirical adaptive rule was derived for the selection of the subsequent n that guarantees a fast convergence of the sequence $\left\{H_{n}\left(\tau \right)\right\}$ to the spectrum $H\left(\tau \right)$. The approximation order n is increased from $\bar{n}$ to $n=\bar{n}+p$, where the step p depends on the ‘speed’ at which index $J\left(\bar{n}\right)$ decreases according to the following switching principle:

(45) $$n=\left\{\begin{array}{c} \bar{n}+10\;\;\;\;\;\;\;\;\;\;\;\;\;\;\;\;\;\;\;\;\;\;\;\;J\left(\bar{n}\right)>10\varepsilon \\ \bar{n}+5\;\;\;\;\;\;\;\;\;\;\;\;\;\;\;\;5\varepsilon <J\left(\bar{n}\right)\leq 10\varepsilon \\ \bar{n}+3\;\;\;\;\;\;\;\;\;\;\;\;\;\;\;2.5\varepsilon <J\left(\bar{n}\right)\leq 5\varepsilon \\ \bar{n}+2\;\;\;\;\;\;\;\;\;1.25\varepsilon <J\left(\bar{n}\right)\leq 2.5\varepsilon \\ \bar{n}+1\;\;\;\;\;\;\;\;\;\;\;\;\;\;\;\varepsilon <J\left(\bar{n}\right)\leq 1.25\varepsilon \end{array}\right.,$$

where ε is a pre-assumed small positive number. Thus, the approximation order n is increased according to the scheme n→n+1 only when index $J\left(n\right)$ is close to the pre-assumed accuracy ε. If $J\left(n\right)\leq \varepsilon$, then in view of the inequality (42), the mean relative error of the real spectrum approximation

(46) $$Q\left(n\right)\leq \frac{1}{m_{n+1}^{2}}\varepsilon +2\left|\frac{1}{m_{n+1}^{2}}-\frac{1}{M_{n+1}}\right|\sqrt{\varepsilon }+\left(\frac{1}{m_{n+1}^{2}}+1-\frac{2}{M_{n+1}}\right).$$

Since, in general, $m_{n+1}<1$, the upper bound in (46) is greater than ε. The estimations (41) and (42) are derived using the lower and upper bounds expressed in (43). During the numerical studies, estimations more rigorous than the above were obtained, depending on the parameter β. In particular in the three tested examples $J\left(n\right)<Q\left(n\right)$, even though the estimate in (44) is larger than $4Q\left(n\right)$.

### 3.6. Algorithm

The Post–Widder approximate relaxation spectrum model can be computed in the following steps.

1. Take the initial value n≥1 of the Post–Widder approximation order and choose the relaxation times $\tau_{j}>0$ for sampling the relaxation spectrum model, j=1,… ,N.
2. For n and n+1 compute spectrum models $H_{n}\left(\tau \right)$ and $H_{n+1}\left(\tau \right)$ using the formula given by Equation (32).
3. Compute the mean relative index $J\left(n\right)$ according to (40) and examine if $J\left(n\right)\leq \varepsilon$, where ε is a small positive number. If yes, go to step 6. Otherwise, put $\bar{n}=n$ and go to step 4.
4. Choose new $n=\bar{n}+p$, selecting the step p according to the rule (45). If p=1, go to step 5. Otherwise, go to step 2.
5. Put $H_{n}\left(\tau \right)=H_{\bar{n}+1}\left(\tau \right)$, compute n+1 order Post–Widder relaxation spectrum model $H_{n+1}\left(\tau \right)$ described by Equation (32) and go to step 3.
6. Stop the procedure, taking $H_{n+1}\left(\tau \right)$ as the relaxation spectrum approximate model.

A few examples of the scheme applicability both for the known and unknown real spectra $H\left(\tau \right)$ are presented in the next section. The calculation scheme presented above will be referred to as the Algorithm.

### 3.7. Identification of the KWW Relaxation Spectrum Model

In this paper it was assumed that the KWW model (1) of the relaxation modulus is known. Its parameters $G_{0}$, $\tau_{r}$ and β are therefore known and can be used to determine the relaxation spectrum model $H_{n}\left(\tau \right)$ according to the presented algorithm. If only measurement data are available, e.g., relaxation modulus measurements collected experimentally in a relaxation test, a two-stage approach should be used. In the first identification stage, the KWW relaxation modulus model should be determined using an appropriate method for identifying this model. Due to the stretched exponential form of the KWW model (1), the problem of its identification is an ill-posed difficult task [77]. Therefore, it is necessary to use special methods for KWW model identification, for example, optimal least-squares approximation combined with Tikhonov regularization [77], as presented in [78], where the guarantee model approximation rule was used to select the regularization parameter stabilizing the problem solution. The identification scheme presented in [78] provides robustness on measurement noises and a strongly consistent estimate of the optimal model, which is independent on the sampling-times used in the rheological experiment. Other known methods for the KWW model identification are: a scheme of the KWW model recovery from the stress–strain experiment data using moments of the measured stress and the corresponding known applied strain rate proposed by Husain and Anderssen [79], a least-squares scheme based on ‘‘artificially’’ created experimental observations and using the Levenberg–Marquardt method to minimize the identification index developed by Sellier et al. [80], and a genetic algorithm used by Yang et al. [81] to fit a model to experiment data optimally in terms of the mean sum of the absolute model error. Recently, Lévy et al. [24] applied the nonlinear least-squares method to the KWW model identification problem transformed into the problem of determining a Debye-type vicarial model. Other KWW model identification methods used by other researchers, in [14,16,31,40,82], can also be applied, as long as the noise-robustness is guaranteed.

Then, in the second identification stage, an arbitrarily high-order Post–Widder approximation of the relaxation spectrum is determined using the approach developed here.

### 3.8. KWW Model Perturbations

The KWW relaxation spectrum model $H_{n}\left(\tau \right)$ determined according to the developed Algorithm depends on the KWW model parameters $G_{0}$, $\tau_{r}$ and β, whose noise-robustness is already guaranteed by the selection of an appropriate scheme for model (1) identification.

Assume that the relaxation modulus model $G\left(t\right)$ (1) is burdened with additive perturbations $\sigma \left(t\right)$, i.e., that the disturbed modulus model

(47) $$\bar{G}\left(t\right)=G\left(t\right)+\sigma \left(t\right)=G_{0}e^{-{\left(\frac{t}{\tau_{r}}\right)}^{\beta }}+\sigma \left(t\right)$$

is used for the spectrum model determination; according to Equation (32) we have

(48) $${\bar{H}}_{n}\left(\tau \right)=\frac{1}{\left(n-1\right)!}\left[\sum_{k=1}^{n}a\left(n,k;\beta \right){\left(\frac{n\tau }{\tau_{r}}\right)}^{\left(n-k+1\right)\beta }\right]\tilde{G}\left(n\tau \right)=H_{n}\left(\tau \right)+\delta H_{n}\left(\tau \right),$$

where the discrepance between the models ${\tilde{H}}_{n}\left(\tau \right)$ and $H_{n}\left(\tau \right)$ is as follows

(49) $$\delta H_{n}\left(\tau \right)={\bar{H}}_{n}\left(\tau \right)-H_{n}\left(\tau \right)=\frac{1}{\left(n-1\right)!}\left[\sum_{k=1}^{n}a\left(n,k;\beta \right){\left(\frac{n\tau }{\tau_{r}}\right)}^{\left(n-k+1\right)\beta }\right]\sigma \left(\tau \right).$$

Assume that there exists $\bar{\sigma }>0$ such that

(50) $$\underset{t>0}{\sup}\left|\sigma \left(t\right)\right|\leq \bar{\sigma },$$

which means that the perturbation $\sigma \left(t\right)$ is uniformly bounded for t>0. Then, by (49) and (29), for any τ>0 and any n≥1 we have

$$\left|\delta H_{n}\left(\tau \right)\right|\leq \frac{1}{\left(n-1\right)!}\left[\sum_{k=1}^{n}a\left(n,k;\beta \right){\left(\frac{n\tau }{\tau_{r}}\right)}^{\left(n-k+1\right)\beta }\right]\bar{\sigma }$$

and

(51) $$\underset{\tau \to 0^{+}}{\lim}\;\left|\delta H_{n}\left(\tau \right)\right|=0.$$

If the relaxation times applied for computation of the model $H_{n}\left(\tau \right)$ are limited to a compact set $T=\left[\tau_{min},\tau_{max}\right]$, then, by (49), the perturbation of the spectrum model $\delta H_{n}\left(\tau \right)$ is uniformly bounded on T, provided that the boundedness property (50) holds.

If the perturbation $\sigma \left(t\right)$ preserves the stretched-exponential-type character of the relaxation modulus, i.e., there exist $\sigma_{0}>0$, 0<θ<1 and ρ>0 such that for all t>0,

$$\left|\sigma \left(t\right)\right|\leq \sigma_{0}e^{-\rho t^{\theta }},$$

then, in view of (49), we obtain the upper-bound

$$\left|\delta H_{n}\left(\tau \right)\right|\leq \frac{1}{\left(n-1\right)!}\left[\sum_{k=1}^{n}a\left(n,k;\beta \right){\left(\frac{n\tau }{\tau_{r}}\right)}^{\left(n-k+1\right)\beta }\right]\sigma_{0}e^{-\rho t^{\theta }},$$

whence the next asymptotic property directly follows

(52) $$\underset{\tau \to \infty }{\lim}\;\left|\delta H_{n}\left(\tau \right)\right|=0.$$

Properties (51) and (52) mean that the ‘zero’ asymptotic properties, specified for the model $H_{n}\left(\tau \right)$ by Theorem 7, are preserved for model ${\bar{H}}_{n}\left(\tau \right)$ (48) determined based on the perturbed model $\bar{G}\left(t\right)$ (47), while guaranteeing that the perturbations remains exponentially bounded. If, additionally, the exponent θ=β and $\rho ={\left(1/\tau_{r}\right)}^{\beta }$, then the spectrum model perturbation $\delta H_{n}\left(\tau \right)$ (49) is maximal for the maximum of the model $H_{n}\left(\tau \right)$ and it is attained for the relaxation time $\tau_{n}^{*}$ defined and specified in Proposition 9.

To summarize, due to the linear dependence of the KWW relaxation spectrum model $H_{n}\left(\tau \right)$ on the relaxation modulus, the perturbation $\delta H_{n}\left(\tau \right)$ (49) of the spectrum model depends linearly on the model (1) perturbation, and the disturbed spectrum model ${\bar{H}}_{n}\left(\tau \right)$ preserves the zero asymptotic properties of the KWW spectrum as long as the perturbation of model (1) preserves its exponential character. After presenting the numerical studies of the Algorithm, we will investigate the behavior of the KWW spectrum model in the presence of perturbations of individual model parameters, using both analytical and numerical tools.

## 4. Results of Simulations for the KWW Spectra

In this section, first, the proposed approach is used to determine models of the KWW spectra described by equations (6) for $\beta =\frac{1}{2}$, (7) for $\beta =\frac{1}{3}$, and (8) when $\beta =\frac{2}{3}$, because the effectiveness of the Post–Widder approach can be easily verified when the real spectrum is exactly known. In 1993, Böhmer et al. [83] presented data concerning stretched exponential relaxation from about 70 amorphous polymeric glass formers (supercooled liquids and disordered crystals). In particular, the exponent β=0.5 has been reported by Plazek and Ngai [84] for poly(methylphenylsiloxane) at the glass temperature $T_{g}=200\;K$ and has been experimentally obtained for sorbitol, dehydroabietic acid, BBKDE, 1,4-cis-polyisoprene, and silicate flint glass [83] (Table I). The coefficients β near 0.5 have been found for: toluene (25%)-β=0.52, BCDE-β=0.51, and polyisobutylene-β=0.55. Recently, the coefficient β=0.504 was reported in [10] for Corning Jade^TM^ glass at temperature 1031 K. Chen et al. [85] applied the KWW model to cross-linked polystyrene at temperature 110 °C with the following parameters: $G_{0}=0.78\;\mathrm{MPa}$, β=0.59 and $\tau_{r}=1.08\;s$ [85] (Table 4). For the purpose of numerical tests of the Algorithm, β=0.59 was replaced here by β=0.5.

Sidebottom et al. [11] described the decay of the alpha-relaxation of ion-conducting glasses using the KWW model with stretching exponents being around 1/3. Recently, the stretching exponent β=0.33 was reported by Farshbaf and Aghjeh [21] for polyester-based PU vitrimer (V-PU) synthesized via nonstoichiometric copolymerization of polycaprolactone (PCL), hexamethylene diisocyanate, and butanediol (BDO) under Sn(Oct)_2_ catalysis, cross-linked via different isocyanate-derived structures tested for intermediate relaxation times at temperature 190 °C. The coefficient β=0.312 was determined by Shirakawa et al. [31] for stress relaxation in bovine femoral cortical bone samples exposed to KOH for 6 h. In turn, the stretching exponent β=0.68 was reported by Farshbaf and Aghjeh [21] for polyester-based PU vitrimer (V-PU) studied for long-scale relaxation times at temperature 160 °C. The coefficient near β=2/3, exactly β=0.632, was reported in [10] for SG80 glass at temperature 800 K.

Since the parameters $G_{0}$ and $\tau_{r}$ do not affect the effectiveness of the scheme, the same parameters, $G_{0}=0.78\;\mathrm{MPa}$ and $\tau_{r}=1.08\;s$, are also used while modeling the KWW spectra for stretching parameters β other than 0.5.

In view of the stretched exponential character of the modulus $G\left(n\tau \right)$, the intervals $T=\left[\tau_{min},\tau_{max}\right]$ applied in the numerical studies cover the range from a few to even dozens of decades of the relaxation times depending on the parameter β, which are summarized in Table A3. Therefore, the relaxation times $\tau_{j}$ used for model determination were sampled according to the exponential rule:

(53) $$\tau_{j}=\tau_{min}\cdot e^{\Delta \tau \cdot \left(j-1\right)},\;\;\Delta \tau =\log\left(\tau_{max}/\tau_{min}\right)/\left(N-1\right),$$

where j=1,…,N and logτ denotes the natural logarithm of τ. Such exponential sampling rules are applied in the numerical studies presented below; however, other sampling schemes can also be applied for the choice of the relaxation times $\tau_{j}$, depending on the needs and planned use of the model. N=50000 sampling points are applied in the numerical studies to guarantee a good estimation of the model quality indices, which for the exponential sampling rule (53) means from three to forty-one points per decade; for details see Table A3. The data given in this table can be used to select the time interval T for a specific value of the stretching exponent β.

For any tested parameter β the Post–Widder approximate models $H_{n}\left(\tau \right)$ are determined for any integer n=1,…,145. The mean relative indices $J\left(n\right)$ and $Q\left(n\right)$ introduced in Equations (40) and (39) were computed. Also, the relaxation times $\tau_{n}^{*}$ and the maxima $H_{n}\left(\tau_{n}^{*}\right)$ defined in (35) were determined and compared with the optimal relaxation times $\tau^{*}$ maximizing the real spectra according to

(54) $$\underset{\tau >0}{\max}\;H\left(\tau \right)=H\left(\tau^{*}\right).$$

The discrepancy between $\tau_{n}^{*}$ and $\tau^{*}$ and the maxima $H_{n}\left(\tau_{n}^{*}\right)$ and $H\left(\tau^{*}\right)$ are expressed by square relative errors

(55) $$ERR\tau^{*}\left(n\right)={\left(\frac{\tau_{n}^{*}-\tau^{*}}{\tau^{*}}\right)}^{2},$$

(56) $$ERRH^{*}\left(n\right)={\left[\frac{H_{n}\left(\tau_{n}^{*}\right)-H\left(\tau^{*}\right)}{H\left(\tau^{*}\right)}\right]}^{2},$$

which characterize the accuracy of reproducing the extreme properties of the real spectra; both the errors were determined in percentages. All the indices are summarized in detailed tables given in the Supplementary Materials.

The Post–Widder relaxation spectra approximate models $H_{n}\left(\tau \right)$ were also determined using the proposed Algorithm for such stretching parameters β for which the compact analytical form of the real spectrum $H\left(\tau \right)$ is unknown. The discrepancy index $J\left(n\right)$ and extreme parameters $\tau_{n}^{*}$ and $H_{n}\left(\tau_{n}^{*}\right)$ were determined and given in Tables S4–S6 in Supplementary Materials. Instead of the indices $Q\left(n\right)$, $ERR\tau^{*}\left(n\right)$ and $ERRH^{*}\left(n\right)$, related to the real spectrum $H\left(\tau \right)$, their analogs were defined by the relation $H_{n}\left(\tau \right)$ to the biggest order model $H_{145}\left(\tau \right)$ as follows

(57) $$Q_{145}\left(n\right)=\frac{1}{N}\sum_{j=1}^{N}{\left[\frac{H_{n}\left(\tau_{j}\right)-H_{145}\left(\tau_{j}\right)}{H_{145}\left(\tau_{j}\right)}\right]}^{2},$$

(58) $$ERR\tau_{145}^{*}\left(n\right)={\left(\frac{\tau_{n}^{*}-\tau_{145}^{*}}{\tau_{145}^{*}}\right)}^{2},$$

(59) $$ERRH_{145}^{*}\left(n\right)={\left[\frac{H_{n}\left(\tau_{n}^{*}\right)-H_{145}\left(\tau_{145}^{*}\right)}{H_{145}\left(\tau_{145}^{*}\right)}\right]}^{2}.$$

For the final models generated by the Algorithm, these indices, expressed in percentages, are given in Table A4.

For the first values of β discussed below, tables with the results of the algorithm’s operation are given in the text of the paper in Appendix A.9, and the remaining ones were moved to the Supplementary Materials. The models’ $H_{n}\left(\tau \right)$ convergence to the real spectrum $H\left(\tau \right)$ or to the model $H_{145}\left(\tau \right)$ is illustrated by the figures, in which the spectra are depicted for the entire time interval T; logarithmic scales are used for the time axes and linear ones for the spectra axes. The small subfigures illustrate the course of the models $H_{n}\left(\tau \right)$ in the near neighborhood of the spectra and their models’ maxima. Since, for fixed β and ε, many of successive models $H_{n}\left(\tau \right)$ generated by the Algorithm practically visually coincide with each other and with the real spectrum, i.e., the curves merge when depicted for the entire range of relaxation times T, in the main figure only selected models, unusual for n=1,11 and the final model, are presented. Furthermore, only selected models will be included in small subfigures if the number of the Algorithm iterations is too large to maintain readability when all the generated models are depicted.

The real spectra $H\left(\tau \right)$ and models $H_{n}\left(\tau \right)$ were simulated in Matlab R2023b, The Mathworks, Inc., Natick, MA, USA; *gamma* function is applied for computing the failing factorials according to (38); the functions *besselk* and *whittakerW* were used for the modeling of spectra given by (7) and (8), respectively.

### 4.1. Stretching Exponent $\beta =\frac{1}{3}$

For the spectrum $H\left(\tau \right)$ (7) with the parameters $\beta =\frac{1}{3}$, $G_{0}=0.78\;\mathrm{MPa}$, and $\tau_{r}=1.08\;s$, following the course of $H\left(\tau \right)$, the relaxation times interval $T=\left[10^{-9},10^{3}\right]\;s$ of twelve decades was taken for the experiment simulations. Thus, N=50000 sampling points means that over 4166 points were applied per one relaxation times decade. The results are summarized in Table S1 in the Supplementary Materials.

The index $J\left(n\right)$ (40) of the successive models discrepancy is smaller than 0.1% already for n=4, is smaller than $10^{-2}$% for n≥9, and for n≥18 it falls below $10^{-3}$%. At the same time, index $Q\left(n\right)$ (39) of the real spectrum $H\left(\tau \right)$ approximation is lower than 1% for n≥13, but it drops below 0.1% only for n≥41. For 13≤n≤40, index $Q\left(n\right)$ falls within the range 0.1–1%, so it is satisfactory.

The application of $\varepsilon >4.2\times 10^{-3}\%$ stops the computational scheme in only two iterations for the model order n=11 (compare Table S1). Then, the discrepancy index $J\left(11\right)=4.17393\times 10^{-3}\%$, which results in the real spectrum approximation error $Q\left(11\right)=1.390260\%$. Therefore, four values of $\varepsilon \leq 10^{-3}\%$ were used; the Algorithm was applied and the calculation process is summarized in Table 1. The determined models $H_{n}\left(\tau \right)$ are evaluated in Table A4, where the number of the scheme iterations I, the order n of determined models, the indices $J\left(n\right)$ and $Q\left(n\right)$, and errors $ERR\tau^{*}\left(n\right)$ and $ERRH^{*}\left(n\right)$ of the real spectrum maximum $\left(\tau^{*},H\left(\tau^{*}\right)\right)$ approximation are given; the extrema $\left(\tau_{n}^{*},H_{n}\left(\tau_{n}^{*}\right)\right)$ of the resulting models $H_{n}\left(\tau \right)$ can be found in Table S1.

**Figure 1:**
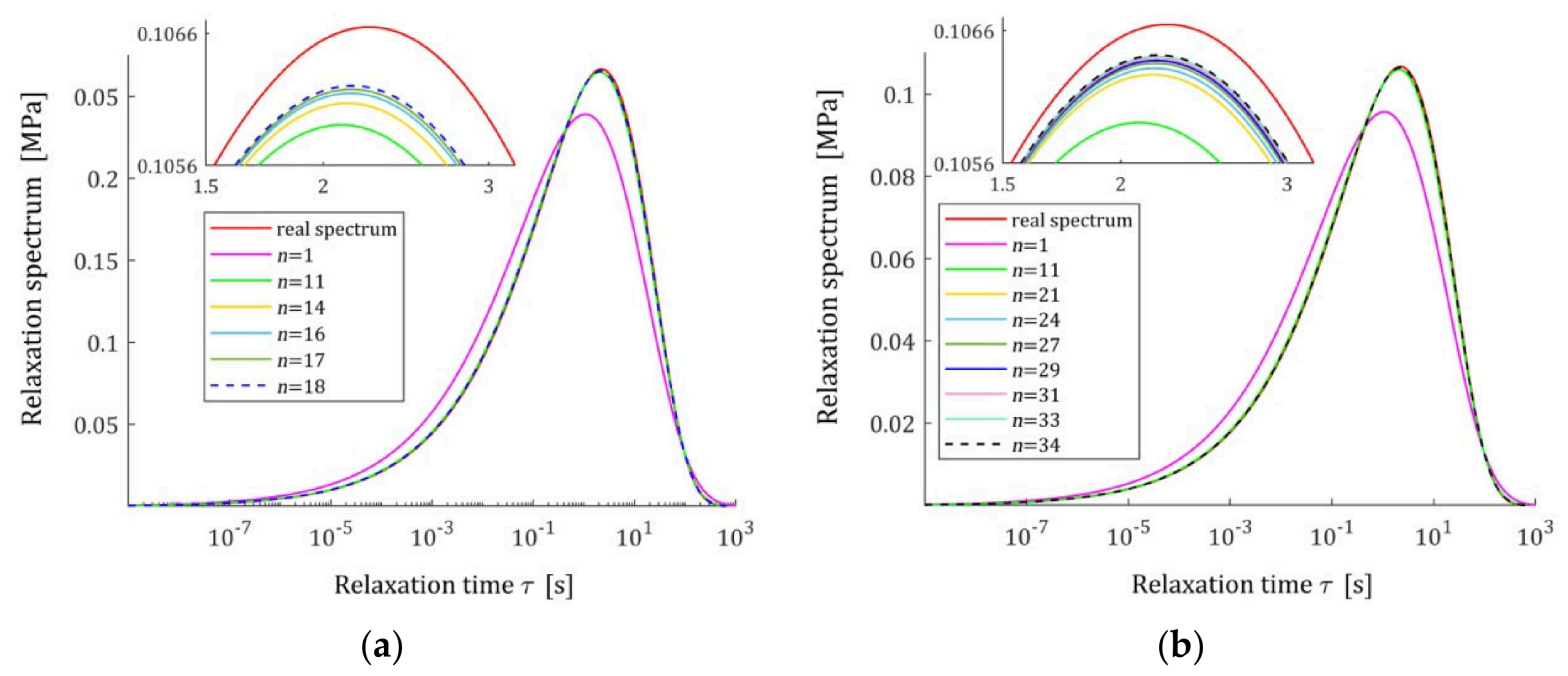
The KWW spectrum $H\left(\tau \right)$ (7) (solid red line) of the parameters: $\beta =\frac{1}{3}$, $G_{0}=0.78\;\mathrm{MPa}$, and $\tau_{r}=1.08\;s$ and the approximate models $H_{n}\left(\tau \right)$ (32) of the order n iterated by the Algorithm for the accuracies ε of the successive models $H_{n}\left(\tau \right)$ and $H_{n+1}\left(\tau \right)$ discrepancy; (**a**) $\varepsilon =10^{-3}\%$, (**b**) $\varepsilon =10^{-4}\%$.

For $\varepsilon =10^{-3}\%$ and $\varepsilon =10^{-4}\%$ the process of determining the successive approximate models $H_{n}\left(\tau \right)$ is illustrated by Figure 1, where the red curve represents the real spectrum. The Alfrey and Doty model $H_{1}\left(\tau \right)$ (11) is depicted by a magenta color curve; the model accuracy index $Q\left(1\right)=196.7\%$ is unacceptable, as expected. For the Schwarzl and Staverman model $H_{2}\left(\tau \right)$ (12) we have $Q\left(2\right)=48.1\%$, which also does not provide a satisfactory approximation of the real spectrum. In the small subfigures all the models iterated for given ε are depicted in the neighborhood of the maxima of the real spectrum and models, while in the main figure only selected models for n=1,11 and the final models for n=18/34 are presented because for n>11 the successive models practically merge with each other and with the real spectrum, which is obvious considering the values of the index $J\left(n\right)$.

We see that for $\varepsilon \leq 10^{-3}\%$ the accuracy of the real spectrum approximation $Q\left(n\right)<0.51\%$, the indices of the real maximum approximation $ERR\tau^{*}\left(n\right)<0.161\%$ and $ERRH^{*}\left(n\right)<1.74\times 10^{-3}\%$, and the number of algorithm iterations not exceeding 11 confirms the effectiveness of the Algorithm.

### 4.2. Stretching Exponent $\beta =\frac{1}{2}$

The real spectrum $H\left(\tau \right)$ (6), corresponding to β=0.5, with parameters $G_{0}=0.78\;\mathrm{MPa}$ and $\tau_{r}=1.08\;s$ was considered. The spectrum $H\left(\tau \right)$ attains the maximum $H\left(\tau^{*}\right)=0.188737\;\mathrm{MPa}$, being independent on $\tau_{r}$, at the relaxation time $\tau^{*}=2\tau_{r}=2.16\;s$.

Following the previous studies [48], the relaxation times interval $T=\left[10^{-6},10^{2}\right]\;s$ was taken, which for N=50000 sampling points $\tau_{j}$ selected according to (53) means 6250 sampling points $\tau_{j}$ per one relaxation time decade. The values of indices $J\left(n\right)$ and $Q\left(n\right)$, the relaxation times $\tau_{n}^{*}$ and the maxima $H_{n}\left(\tau_{n}^{*}\right)$ with the respective square errors $ERR\tau^{*}\left(n\right)$ and $ERRH^{*}\left(n\right)$ of the real maximum $\tau^{*},H\left(\tau^{*}\right)$ approximation, determined for the approximation orders 1≤n≤145, are summarized in Table S2 in the Supplementary Materials. Model accuracy index $Q\left(n\right)$ drops below 1% for n≥27, below 0.5% for n≥38, and below 0.1% only for n≥84. The discrepancy index $J\left(n\right)$ is then of the order of 5.5 × 10^−4^%, which means that the next iterations of the Algorithm only slightly change the course of the subsequent models $H_{n}\left(\tau \right)$.

**Figure 2:**
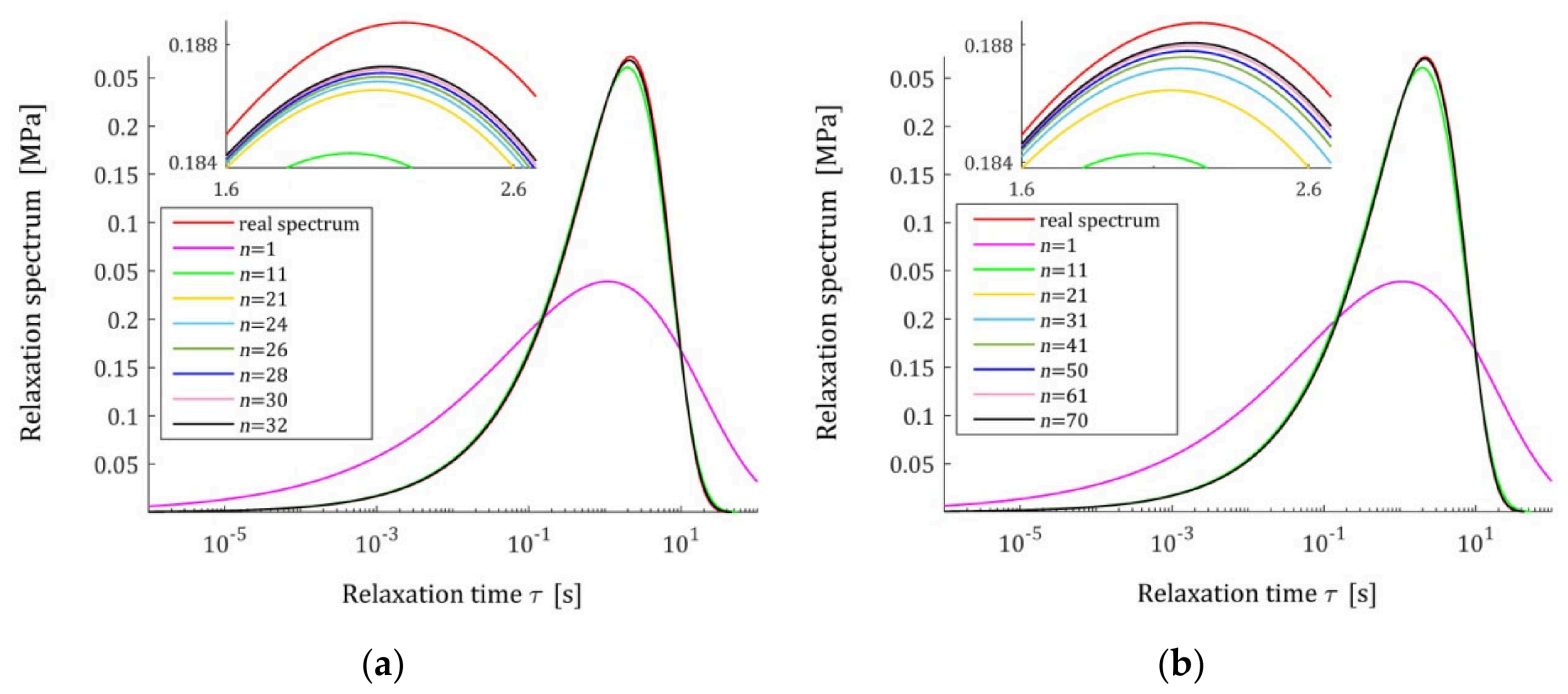
The KWW spectrum $H\left(\tau \right)$ (6) (solid red line) of the parameters β=0.5, $G_{0}=0.78\;\mathrm{MPa}$, and $\tau_{r}=1.08\;s$ and the approximate models $H_{n}\left(\tau \right)$ (32) of the order n iterated by the Algorithm for the accuracies ε of the successive models $H_{n}\left(\tau \right)$ and $H_{n+1}\left(\tau \right)$ discrepancy: (**a**) $\varepsilon =10^{-2}\%$, (**b**) $\varepsilon =10^{-3}\%$.

Next, the Algorithm was applied for four $\varepsilon \leq 10^{-2}\%$; the processes of generating subsequent spectrum models $H_{n}\left(\tau \right)$ are presented in Table A5, and the quality of the resulting models is evaluated in Table A4. The Algorithm generates model $H_{87}\left(\tau \right)$ of accuracy $Q\left(87\right)=9.2\times 10^{-2}\%$ only for $\varepsilon =5\times 10^{-4}\%$; however, for $\varepsilon =10^{-3}\%$ the model accuracy $Q\left(70\right)=0.143\%$ is also satisfactory, and the estimation of the real spectrum maximum by obtained model $H_{70}\left(\tau \right)$ is characterized by errors less than $10^{-2}$% (compare Table A4). Also the 20 iterations needed to obtain model $H_{70}\left(\tau \right)$ are satisfactory. Figure 2 graphically illustrates the process of determining the models $H_{32}\left(\tau \right)$ and $H_{70}\left(\tau \right)$ by the Algorithm; for $\varepsilon =10^{-2}\%$ all the iterated models were depicted in the small subfigure, while for $\varepsilon =10^{-3}\%$ only 7 out of 20 models generated by the Algorithm were placed. Note that here the Alfrey and Doty model $H_{1}\left(\tau \right)$ (11), depicted by the magenta curve, gives especially poor real spectrum approximation.

### 4.3. Stretching Exponent $\beta =\frac{2}{3}$

Following the course of the spectrum $H\left(\tau \right)$ (8) with $G_{0}=0.78\;\mathrm{MPa}$ and $\tau_{r}=1.08\;s$, the relaxation times interval $T=\left[10^{-4},10\right]$ was assumed, which for N=50000 sampling points means 10,000 points per decade. The results of simulations for 1≤n≤145 are summarized in Table S3; the indices $J\left(n\right)$, $Q\left(n\right)$, $ERR\tau^{*}\left(n\right)$, $ERRH^{*}\left(n\right)$ and model maxima $\tau_{n}^{*}$, $H_{n}\left(\tau_{n}^{*}\right)$ are collected. The model accuracy index $Q\left(n\right)$ is smaller than 1% for n≥20, it drops below 0.5% for n≥29, and it is not greater than 0.1% for n≥68. For n≥68, the errors of the real spectrum maximum approximation are smaller than 5 × 10^−2^%; simultaneously, the discrepancy index $J\left(n\right)$ is of the order of 5.4 × 10^−4^%, i.e., the next iterations of the Algorithm only slightly change the course of the subsequent models $H_{n}\left(\tau \right)$.

**Figure 3:**
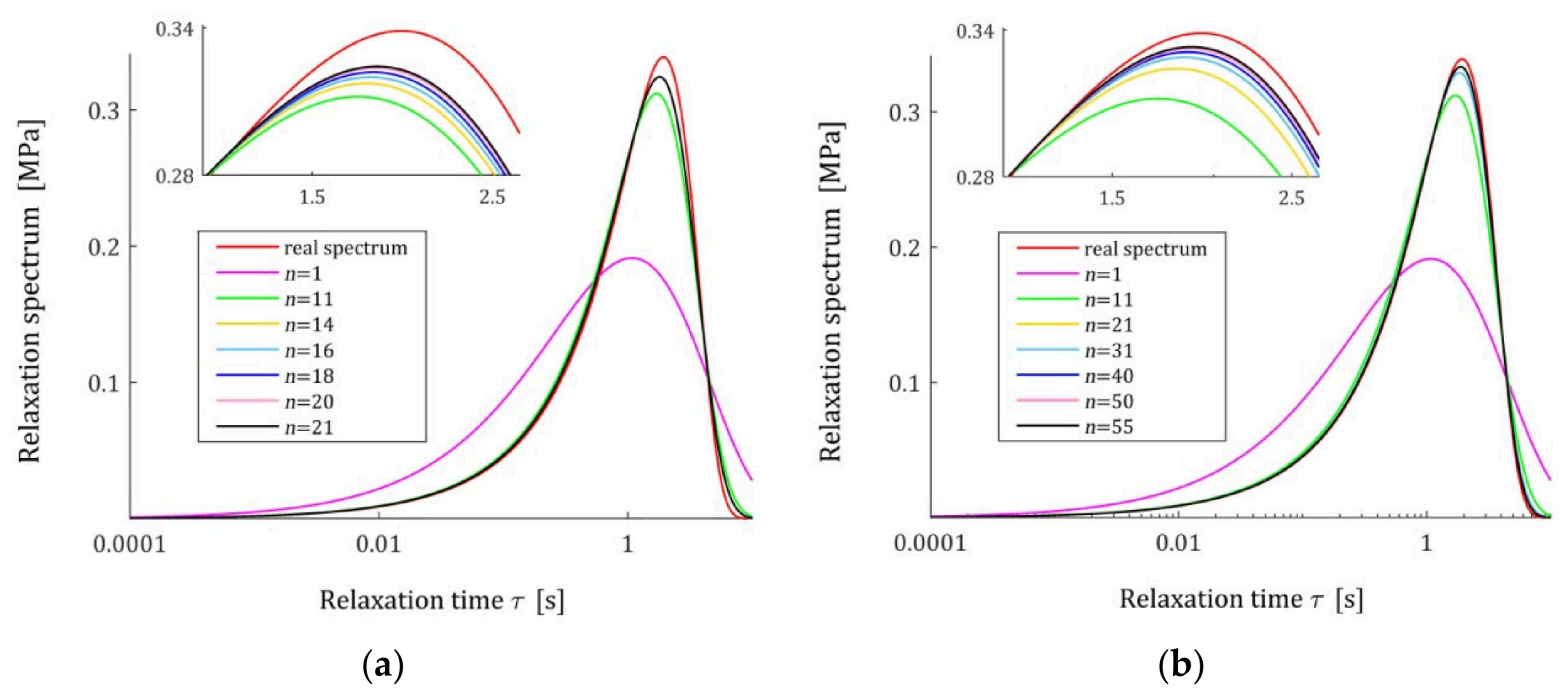
The KWW spectrum $H\left(\tau \right)$ (8) (solid red line) of the parameters $\beta =\frac{2}{3}$, $G_{0}=0.78\;\mathrm{MPa}$, and $\tau_{r}=1.08\;s$ and the approximate models $H_{n}\left(\tau \right)$ (32) of the order n iterated by the Algorithm for the accuracies ε of the successive models $H_{n}\left(\tau \right)$ and $H_{n+1}\left(\tau \right)$ discrepancy: (**a**) $\varepsilon =10^{-2}\%$, (**b**) $\varepsilon =10^{-3}\%$.

For selected $\varepsilon \leq 10^{-2}\%$, the Algorithm computations are summarized in Table A6. Depending on ε, from 7 to 21 iterations were necessary to determine the resulting models $H_{n}\left(\tau \right)$ evaluated in Table A4. For $\varepsilon =10^{-2}\%$, index $Q\left(21\right)=0.86\%$, while for $\varepsilon =10^{-3}\%$, the accuracy $Q\left(55\right)=0.16\%$ means that the determined model $H_{55}\left(\tau \right)$ guarantees a good approximation of the real spectrum. The process of generating the models $H_{21}\left(\tau \right)$ and $H_{55}\left(\tau \right)$ for $\varepsilon =10^{-2}\%$ and $\varepsilon =10^{-3}\%$, respectively, is illustrated in Figure 3.

### 4.4. Application of the Algorithm for Other β

During the numerical studies and the Algorithm application for stretching parameters β, for which the compact analytical form of the spectrum $H\left(\tau \right)$ is unknown, as previously, N=50000 sampling points were used to accurately evaluate index $J\left(n\right)$; the corresponding relaxation times intervals T are characterized in Table A3. Index $J\left(n\right)$ and the extreme parameters $\tau_{n}^{*}$, $H_{n}\left(\tau_{n}^{*}\right)$ of the spectra models $H_{n}\left(\tau \right)$ were determined for the approximation orders n=1,…,145. These detailed data are given in Tables S4, S7 and S10 in Supplementary Materials; however, to limit the size of the tables, they are given only for n=1,…,100 for β=0.1,0.2,0.45, 0.55 and for n=1,…,125 for β=0.75, 0.85, when these indices change significantly, and for n=140,…,145, where the changes in indices are small and do not affect the course of the algorithm iterations.

Parameter β=0.201 was reported in [86] for a high temperature epoxy during the cure for the degree of cure 0.8. Recently, the stretching exponent β=0.22 was determined by Farshbaf and Aghjeh [21] for a polyester-based PU vitrimer (V-PU) tested for intermediate relaxation times at temperature 170 °C. The stretching exponent β=0.75 was accepted by Farshbaf and Aghjeh [21] for polyester-based PU vitrimer (V-PU) at temperature 180 °C, while β=0.84 was determined by Farshbaf and Aghjeh [21] for a polyether-based vitrimer (V-PUG) tested at temperature 160 °C

For each considered β, the Algorithm was applied and the processes of calculations are presented in Tables S5, S6, S8, S9, S11 and S12 in the Supplementary Materials. Data on the Algorithm’s operation and the designated models $H_{n}\left(\tau \right)$ are presented in Table A4; in particular the indices $Q_{145}\left(n\right)$, $ERR\tau_{145}^{*}\left(n\right)$ and $ERRH_{145}^{*}\left(n\right)$, introduced by Equations (57), (58) and (59), respectively, and related to the model $H_{145}\left(\tau \right)$ are given.

#### 4.4.1. Stretching Exponents β=0.1 and β=0.2

For the spectra $H\left(\tau \right)$ of parameters β=0.1 and β=0.2, with the common $G_{0}=0.78\;\mathrm{MPa}$ and $\tau_{r}=1.08\;s$, very wide intervals T were applied—see Table A3—covering, respectively, forty-one and twenty relaxation time decades, which for N=50000 sampling points means, respectively, over 1219 and 2500 points applied per decade. The results of the simulations are summarized in Table S3. For this small stretching, exponents index $J\left(n\right)$ decreases very fast with increasing approximation order n and is smaller than $10^{-3}\%$ already for n≥5 for β=0.1 and for n≥7, when β=0.2. For β=0.1, the analysis of the data from Table S4 indicates that, for the pre-assumed $\varepsilon >2.59\times 10^{-5}\%$, the application of the Algorithm results in only two iterations; the computations stop for the model order n=11 and give the index $J\left(11\right)=2.583\times 10^{-5}\%$. Similarly, for β=0.2 and the pre-assumed $\varepsilon >1.24\times 10^{-4}\%$ the Algorithm computations also stop after two iterations for n=11 and the index $J\left(11\right)=1.24\times 10^{-4}\%$; compare data in the first rows of Table S4. The quality of the determined model $H_{11}\left(\tau \right)$ is evaluated in Table A4, by using the indices $Q_{145}\left(n\right)$, $ERR\tau_{145}^{*}\left(n\right)$ and $ERRH_{145}^{*}\left(n\right)$ related to model $H_{145}\left(\tau \right)$. The model accuracy index $Q_{145}\left(11\right)<7\times 10^{-3}\%$ is also satisfactory; however, the index $ERR\tau_{145}^{*}\left(11\right)$, equal to 0.21% and 0.26% for β=0.1 and β=0.2, respectively, can be improved.

**Figure 4:**
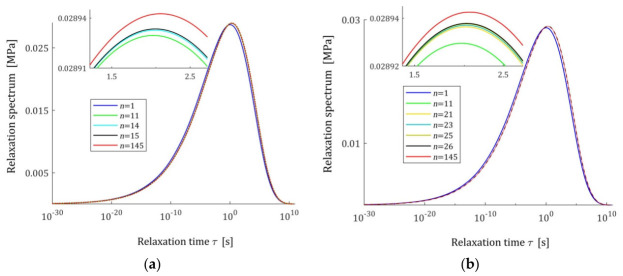
For the KWW spectrum $H\left(\tau \right)$ (4) of the parameters $G_{0}=0.78\;\mathrm{MPa}$, β=0.1 and $\tau_{r}=1.08\;s$, the approximate models $H_{n}\left(\tau \right)$ (32) of the order n iterated by the Algorithm for the accuracies ε of the successive models $H_{n}\left(\tau \right)$ and $H_{n+1}\left(\tau \right)$ discrepancy: (**a**) $\varepsilon =10^{-5}\%$, (**b**) $\varepsilon =10^{-6}\%$.

**Figure 5:**
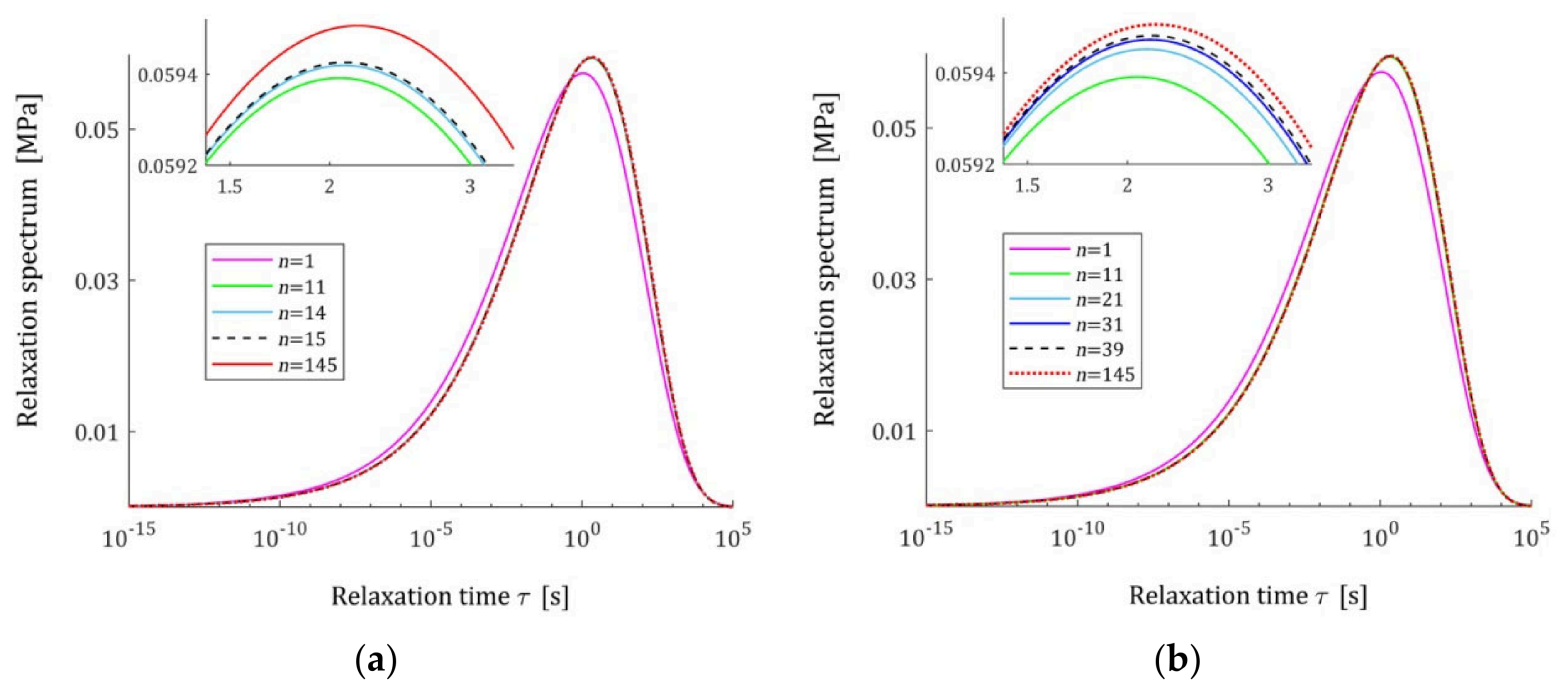
For the KWW spectrum $H\left(\tau \right)$ of the parameters $G_{0}=0.78\;\mathrm{MPa}$, β=0.2 and $\tau_{r}=1.08\;s$, the approximate models $H_{n}\left(\tau \right)$ (32) of the order n iterated by the Algorithm for the accuracies ε of the successive models $H_{n}\left(\tau \right)$ and $H_{n+1}\left(\tau \right)$ discrepancy: (**a**) $\varepsilon =10^{-5}\%$, (**b**) $\varepsilon =10^{-6}\%$.

For both exponents β the Algorithm is applied assuming $\varepsilon =10^{-5},5\times 10^{-6},\;10^{-6}\%$; computational processes are recapitulated in Tables S5 and S6; successive iterated spectra for $\varepsilon =10^{-5},10^{-6}\%$ are depicted in Figure 4 and Figure 5; the determined models are evaluated in Table A4. In Figure 4 and Figure 5, the plots of models $H_{145}\left(\tau \right)$ are shown in red. Models $H_{n}\left(\tau \right)$ for n≥11 and the model $H_{145}\left(\tau \right)$ visually practically coincide in these figures; however, the small subfigures demonstrate small differences between them in the near neighborhood of their maxima. Reducing ε increases the number of Algorithm iterations, and the order of the determined model also grows. A better estimation of the real spectrum maximum than for $H_{11}\left(\tau \right)$ is demonstrated by small subfigures in Figure 4 and Figure 5 and decreased indices $ERR\tau_{145}^{*}\left(n\right)$ and $ERRH_{145}^{*}\left(n\right)$. Summarizing, the parameter $\varepsilon =10^{-5}\%$ can be recommended for small stretching exponents as resulting in satisfactory spectrum $H_{145}\left(\tau \right)$ approximation.

#### 4.4.2. Stretching Exponents β=0.45 and β=0.55

**Figure 6:**
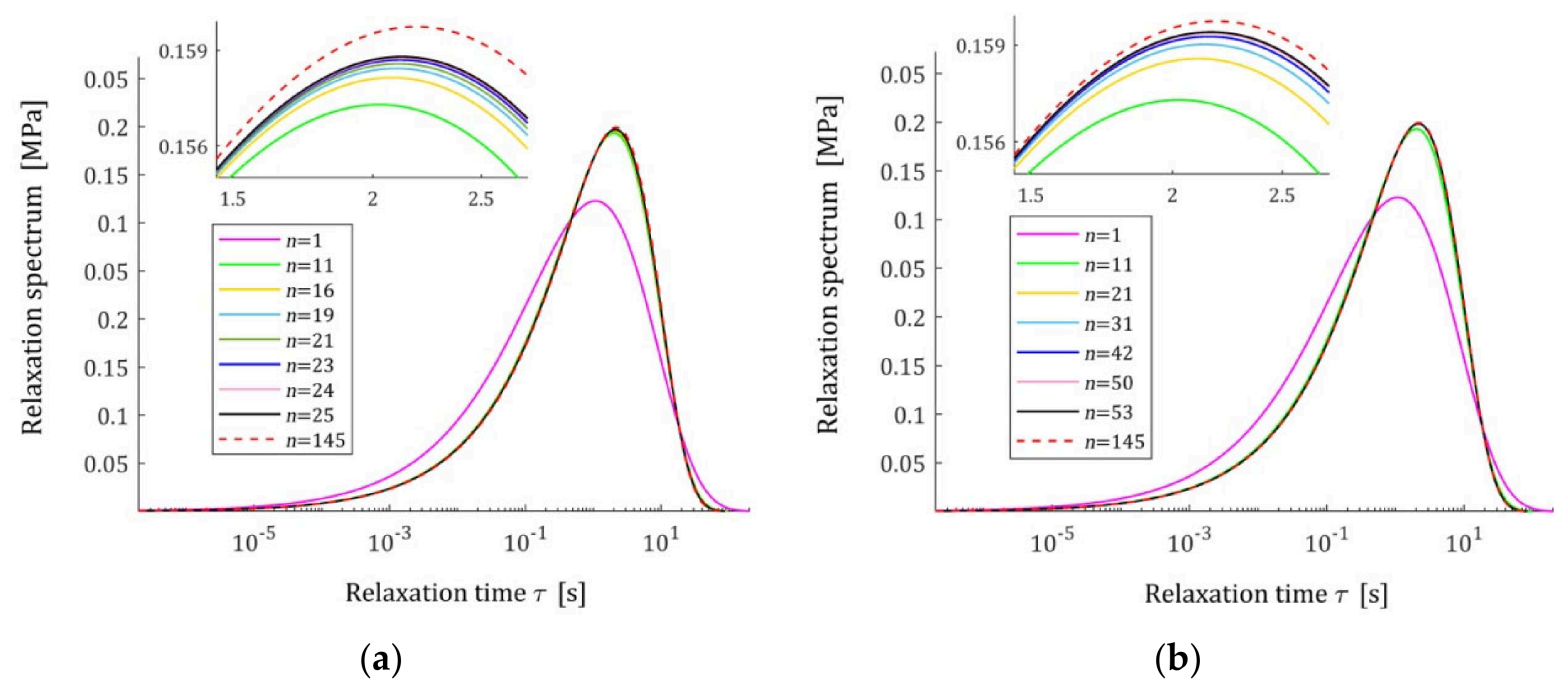
For the KWW spectrum $H\left(\tau \right)$ (4) of the parameters $G_{0}=0.78\;\mathrm{MPa}$, β=0.45, and $\tau_{r}=1.08\;s$, the approximate models $H_{n}\left(\tau \right)$ (32) of the order n iterated by the Algorithm for the accuracies ε of the successive models $H_{n}\left(\tau \right)$ and $H_{n+1}\left(\tau \right)$ discrepancy: (**a**) $\varepsilon =10^{-2}\%$ and (**b**) $\varepsilon =10^{-3}\%$.

**Figure 7:**
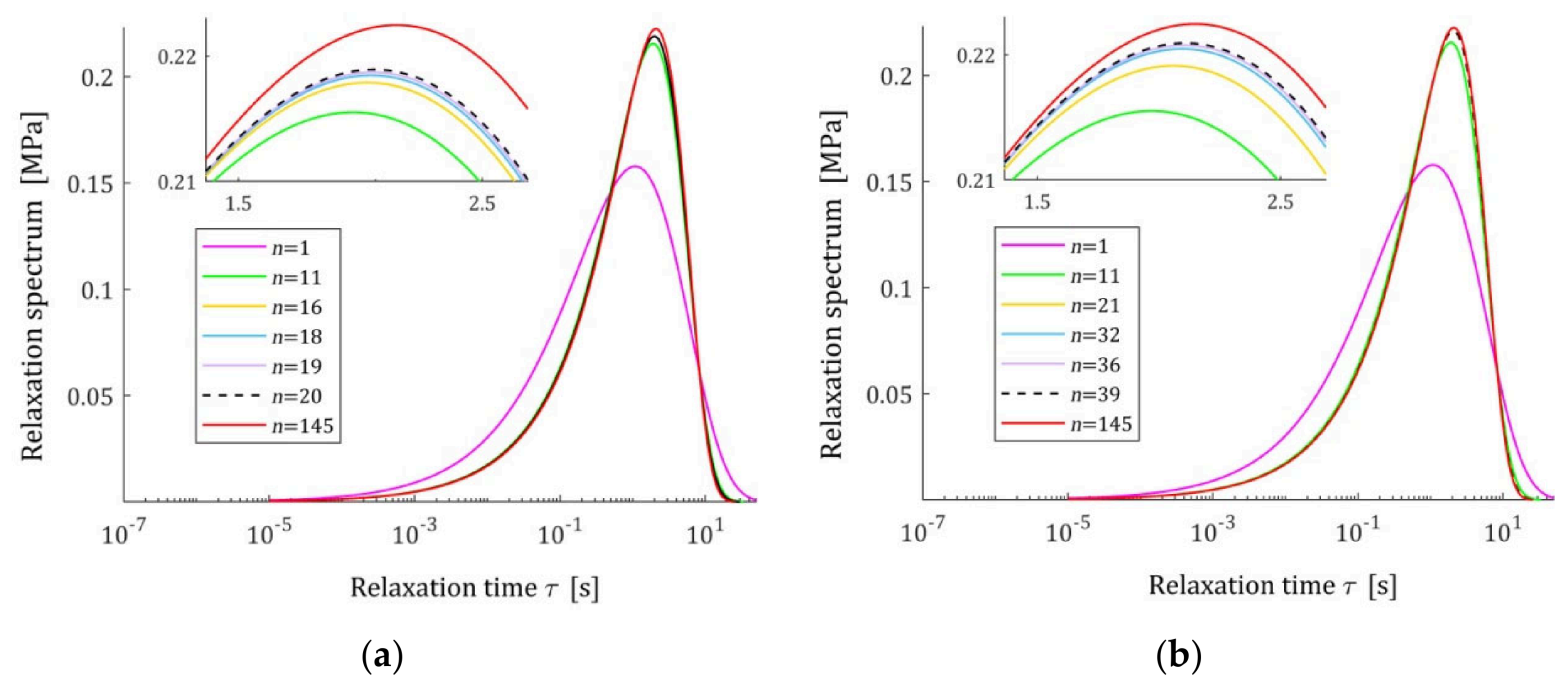
For the KWW spectrum $H\left(\tau \right)$ (4) of the parameters $G_{0}=0.78\;\mathrm{MPa}$, β=0.55, and $\tau_{r}=1.08\;s$, the approximate models $H_{n}\left(\tau \right)$ (32) of the order n iterated by the Algorithm for the accuracies ε of the successive models $H_{n}\left(\tau \right)$ and $H_{n+1}\left(\tau \right)$ discrepancy: (**a**) $\varepsilon =10^{-2}\%$ and (**b**) $\varepsilon =10^{-3}\%$.

The results of simulations given in Table S7 demonstrate that the index $J\left(n\right)$ decreases with increasing n  slower than for $\beta \leq \frac{1}{3}$ and similarly as for β=0.5. Therefore, to examine the Algorithm effectiveness, the same accuracies ε were assumed as for β=0.5. The processes of computations are recapitulated in Tables S8 and S9 and illustrated in Figure 6 and Figure 7. The determined models are evaluated in Table A4.

For β=0.55 and the models discrepancy accuracy $\varepsilon =10^{-2}\%$, index $Q_{145}\left(n\right)$, greater than 1.58%, is too large and more than 7.58 times larger than for β=0.45 for the same accuracy ε. This is due to a noticeably worse approximation of the spectrum maximum, which is visible in Figure 7a. For $\varepsilon =10^{-3}\%$, the approximation of the model $H_{145}\left(\tau \right)$ by the determined models $H_{53}\left(\tau \right)$ and $H_{39}\left(\tau \right)$ is fine for both stretching exponents. For β=0.55, the index $Q_{145}\left(39\right)=0.3\%$ is satisfactory, which is confirmed by the course of the models in Figure 7b. Further reduction of ε results in an almost perfect approximation of the model $H_{145}\left(\tau \right)$, which is illustrated in Figure S1 in Supplementary Materials for models $H_{48}\left(\tau \right)$ and $H_{74}\left(\tau \right)$ determined for $\varepsilon =5\times 10^{-4}\%$ and $\varepsilon =10^{-4}\%$, respectively. Especially, for $\varepsilon =10^{-4}\%$, the index $Q_{145}\left(74\right)=3.75\times 10^{-2}\%$ means a very accurate approximation of $H_{145}\left(\tau \right)$.

#### 4.4.3. Stretching Exponents β=0.75 and β=0.85

**Figure 8:**
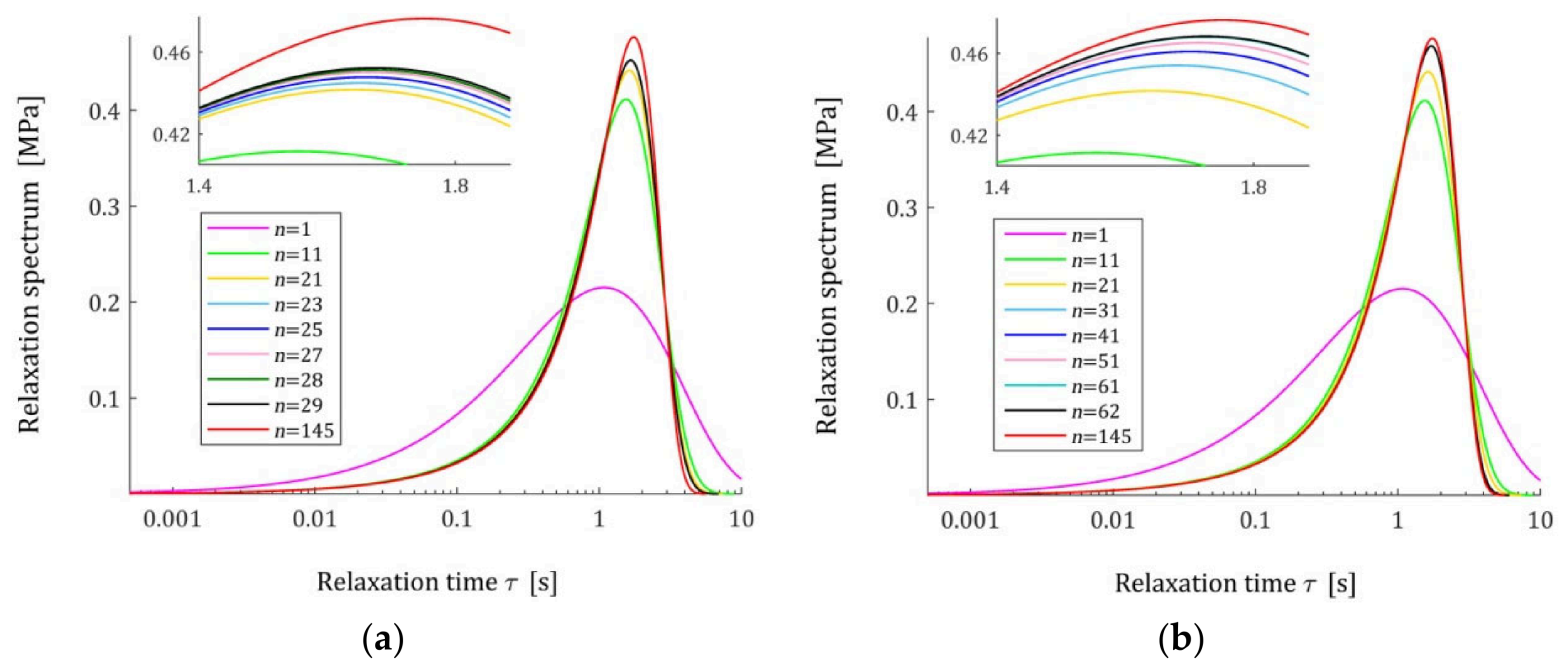
For the KWW spectrum $H\left(\tau \right)$ (4) of the parameters $G_{0}=0.78\;\mathrm{MPa}$, β=0.75, and $\tau_{r}=1.08\;s$, the approximate models $H_{n}\left(\tau \right)$ (32) of the order n iterated by the Algorithm for the accuracies ε of the successive models $H_{n}\left(\tau \right)$ and $H_{n+1}\left(\tau \right)$ discrepancy; (**a**) $\varepsilon =10^{-2}\%$ and (**b**) $\varepsilon =10^{-3}\%$.

**Figure 9:**
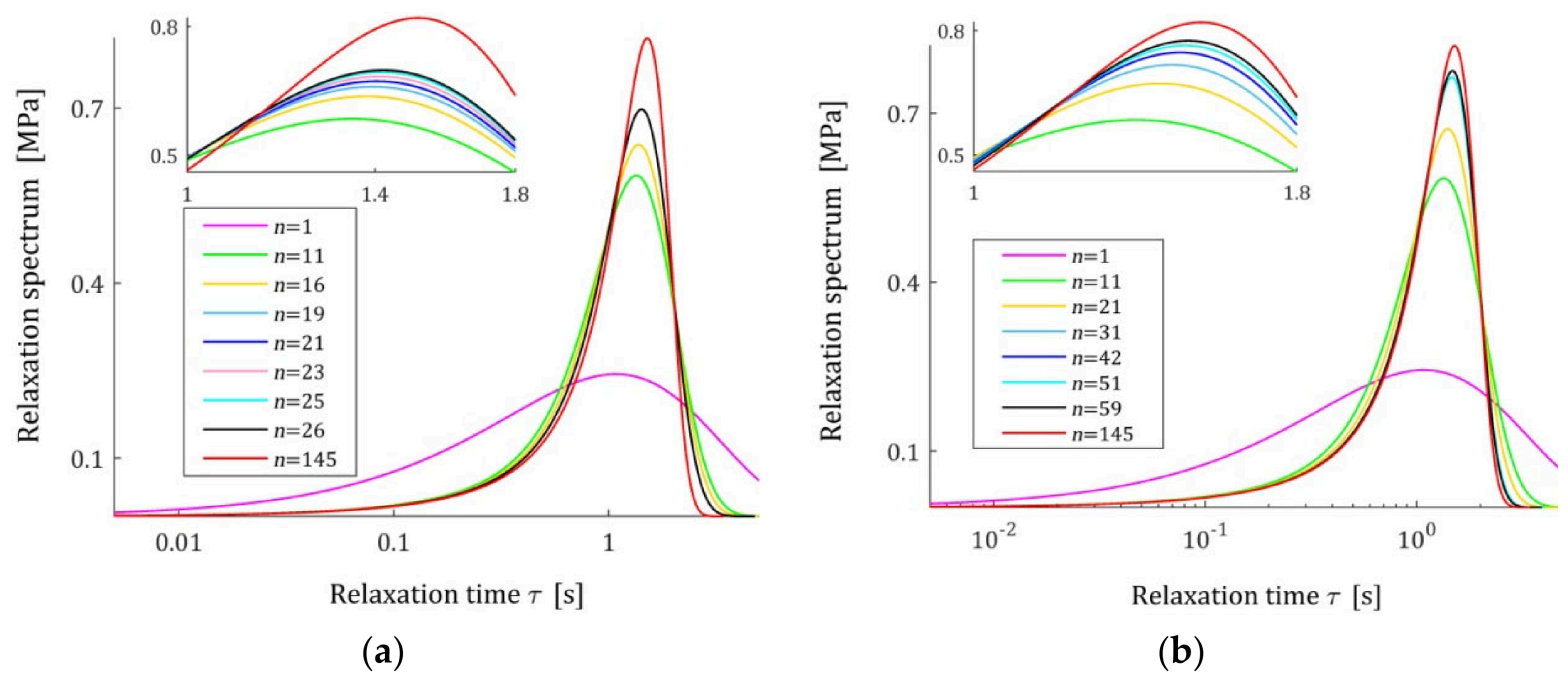
For the KWW spectrum $H\left(\tau \right)$ (4) of the parameters $G_{0}=0.78\;\mathrm{MPa}$, β=0.85, and $\tau_{r}=1.08\;s$, the approximate models $H_{n}\left(\tau \right)$ (32) of the order n iterated by the Algorithm for the accuracies ε of the successive models $H_{n}\left(\tau \right)$ and $H_{n+1}\left(\tau \right)$ discrepancy; (**a**) $\varepsilon =10^{-2}\%$ and (**b**) $\varepsilon =10^{-3}\%$.

By the inspection of the data summarized in Table S10 we see that the index $J\left(n\right)$ decreases slower than for smaller β; therefore, more iterations are needed for the Algorithm to generate the final model, especially for very small ε. The computational processes are summarized in Tables S11 and S12 for the same ε that were previously applied for β=0.55 and illustrated in Figure 8 and Figure 9. Models $H_{29}\left(\tau \right)$ and $H_{26}\left(\tau \right)$, generated for $\varepsilon =10^{-2}\%$, are unsatisfactory; compare Figure 8a and Figure 9a and the indices $Q_{145}\left(n\right)$ in Table A4. Models $H_{62}\left(\tau \right)$ and $H_{59}\left(\tau \right)$, obtained for $\varepsilon =10^{-3}\%$, guarantee much better approximation of $H_{145}\left(\tau \right)$; see Figure 8b and Figure 9b. However, the indices $Q_{145}\left(62\right)=2.22\%$ and $Q_{145}\left(59\right)=1.99\%$ are not satisfactory. Further reducing ε below $10^{-3}\%$ improves the index $Q_{145}\left(n\right)$ and the indices of the maximum estimation. Detailed information on the generated successive approximations for $\varepsilon =5\times 10^{-4}\%$ and $\varepsilon =10^{-4}\%$ is given in the last two columns of Tables S11 and S12 and illustrated in Figures S2 and S3.

In particular, for β=0.75 and $\varepsilon =5\times 10^{-4}\%$, the model accuracy index $Q_{145}\left(76\right)=0.878\%$ noticeably improves $Q_{145}\left(62\right)=2.22\%$. For β=0.75 and $\varepsilon =5\times 10^{-4}\%$, the index $Q_{145}\left(74\right)=0.81\%$ is more than twice as good as $Q_{145}\left(59\right)=1.99\%$. Similarly, the estimation of the spectra maxima improves, particularly for β=0.75, as can be seen by comparing Figure 8b and Figure S2a. For $\varepsilon =10^{-4}\%$ the spectra $H_{123}\left(\tau \right)$ and $H_{124}\left(\tau \right)$ practically coincide with $H_{145}\left(\tau \right)$, which is well illustrated by small subfigures of Figures S2b and S3b, where the vertical axes ranges are twice as small as in the previous figures for the corresponding β. However, the orders of models $H_{123}\left(\tau \right)$ and $H_{124}\left(\tau \right)$ may turn out to be too large from a practical point of view, whence both $\varepsilon =10^{-3}\%$ and, above all, $\varepsilon =5\times 10^{-4}\%$ are sufficient to obtain a good estimate of the spectrum.

### 4.5. Sensitivity to Perturbations of the KWW Model Parameterss

The n-th order Post–Widder approximate model $H_{n}\left(\tau \right)$ given by Equations (29) or (32), depends on the KWW relaxation modulus model parameters $G_{0}$, $\tau_{r}$ and β. The impact of their perturbations is analyzed; the fundamental sensitivity analysis technique utilizing partial differentiation is applied. To analyze the influence of perturbations of the stretching exponent, the differential approach was combined with numerical studies.

Since the coefficients $a\left(n,k;\beta \right)$ defined by Equations (25)–(27) depend only on the stretching exponent, i.e., are independent of $\tau_{r}$ and $G_{0}$, by two-sided differentiation of Equation (32) with respect to the initial shear modulus $G_{0}$, in view of (1), we have

$$\frac{\partial H_{n}\left(\tau \right)}{\partial G_{0}}=\frac{1}{\left(n-1\right)!}\left[\sum_{k=1}^{n}a\left(n,k;\beta \right){\left(\frac{n\tau }{\tau_{r}}\right)}^{\left(n-k+1\right)\beta }\right]e^{-{\left(\frac{n\tau }{\tau_{r}}\right)}^{\beta }}=\frac{H_{n}\left(\tau \right)}{G_{0}}.$$

The middle formula means that the sensitivity to $G_{0}$ perturbations does not depend on the value of this parameter. In turn, the right formula means that since this sensitivity preserves the monotonicity of the model $H_{n}\left(\tau \right)$, it is the largest for the relaxation time $\tau_{n}^{*}$ for which the relaxation spectrum reaches the maximum, in accordance with Theorem 8, and that it vanishes into zero for $\tau \to 0^{+}$ and τ→∞, in accordance with Theorem 7, i.e., it is negligibly small for small and large relaxation times τ.

Both the models (1) and (32) depend on the fraction $\tau /\tau_{r}$, and not on the relaxation times τ and $\tau_{r}$ separately. Therefore, the next equation holds

(60) $$\frac{\partial H_{n}\left(\tau \right)}{\partial \tau_{r}}=-\frac{\tau }{\tau_{r}}\frac{dH_{n}\left(\tau \right)}{d\tau },$$

which, by Equation (A16) describing the spectrum model derivative with respect to relaxation time τ, can be expressed as follows

$$\begin{array}{c} \frac{\partial H_{n}\left(\tau \right)}{\partial \tau_{r}}=-\frac{\beta }{\tau_{r}\left(n-1\right)!}\left[\sum_{k=1}^{n}a\left(n,k;\beta \right)\left(n-k+1\right){\left(\frac{n\tau }{\tau_{r}}\right)}^{\left(n-k+1\right)\beta }\right]G\left(n\tau \right)+\\ \frac{\beta }{\tau_{r}\left(n-1\right)!}\left[\sum_{k=1}^{n}a\left(n,k;\beta \right){\left(\frac{n\tau }{\tau_{r}}\right)}^{\left(n-k+2\right)\beta }\right]G\left(n\tau \right). \end{array}$$

Thus, the sensitivity index $\frac{\partial H_{n}\left(\tau \right)}{\partial \tau_{r}}$ vanishes into zero for $\tau \to 0^{+}$ and τ→∞, i.e., asymptotically, the perturbations of this parameter do not affect the spectrum model. Additionally, according to the formula (60), the perturbations of $\tau_{r}$ does not affect the model $H_{n}\left(\tau \right)$ at its maximum, since for the maximum $\tau_{n}^{*}$ defined by Equation (35) the right-hand side of (60) is equal to zero. Bearing in mind the monotonicity of the model $H_{n}\left(\tau \right)$, confirmed above by the numerical studies, based on (60), we see that for $\tau <\tau_{n}^{*}$ a positive perturbation of the parameter $\tau_{r}$ decreases the value of the model $H_{n}\left(\tau \right)$, while for $\tau >\tau_{n}^{*}$ the same perturbation of $\tau_{r}$ increases the value of the $H_{n}\left(\tau \right)$. This means that with a positive perturbation of $\tau_{r}$, the maximum of the perturbed model is reached for the relaxation time $\tau >\tau_{n}^{*}$, and the original $H_{n}\left(\tau \right)$ and perturbed ${\bar{H}}_{n}\left(\tau \right)$ models intersect for time $\tau_{n}^{*}$.

Finally, we investigate the sensitivity of the model $H_{n}\left(\tau \right)$ to perturbations of the stretching exponent. In Appendix A.10 the following formula is derived

(61) $$\frac{\partial H_{n}\left(\tau \right)}{\partial \beta }=\frac{1}{\left(n-1\right)!}\left[\sum_{k=1}^{n}\frac{\partial a\left(n,k;\beta \right)}{\partial \beta }{\left(\frac{n\tau }{\tau_{r}}\right)}^{\left(n-k+1\right)\beta }\right]G\left(n\tau \right)+\frac{n}{\beta }\left[H_{n}\left(\tau \right)-H_{n+1}\left(\frac{n}{n+1}\tau \right)\right]\log\left(\frac{n\tau }{\tau_{r}}\right),$$

where the partial derivatives $\frac{\partial a\left(n,k;\beta \right)}{\partial \beta }$ are defined by the equations

(62) $$\frac{\partial a\left(n+1,k;\beta \right)}{\partial \beta }=\left[n-\beta \left(n-k+2\right)\right]\frac{\partial a\left(n,k-1;\beta \right)}{\partial \beta }-\left(n-k+2\right)a\left(n,k-1;\beta \right)+a\left(n,k;\beta \right)+\beta \frac{\partial a\left(n,k;\beta \right)}{\partial \beta }$$

for 2≤k≤n and the boundary conditions with respect to k:

(63) $$\frac{\partial a\left(n,1;\beta \right)}{\partial \beta }=n\beta^{n-1},$$

(64) $$\frac{\partial a\left(n,n;\beta \right)}{\partial \beta }={\left(-1\right)}^{n+1}{\left(\beta \right)}_{n}\sum_{k=1}^{n}\frac{1}{\beta -k+1}.$$

Considering the stretched exponential nature of the relaxation modulus in formulas (32), (A22) and in the first term of the right-hand side of (61), it is easy to check that the influence of β-perturbations on the relaxation spectrum model is negligible when $\tau \to 0^{+}$ and τ→∞. Generally, analytical sensitivity analysis of the relaxation spectrum model to β perturbations based on Equation (61) is significantly more difficult than for the other two parameters. Therefore, numerical studies were carried out, and for β considered in previous studies, the influence of the parameter perturbations on the determined model was examined.

**Figure 10:**
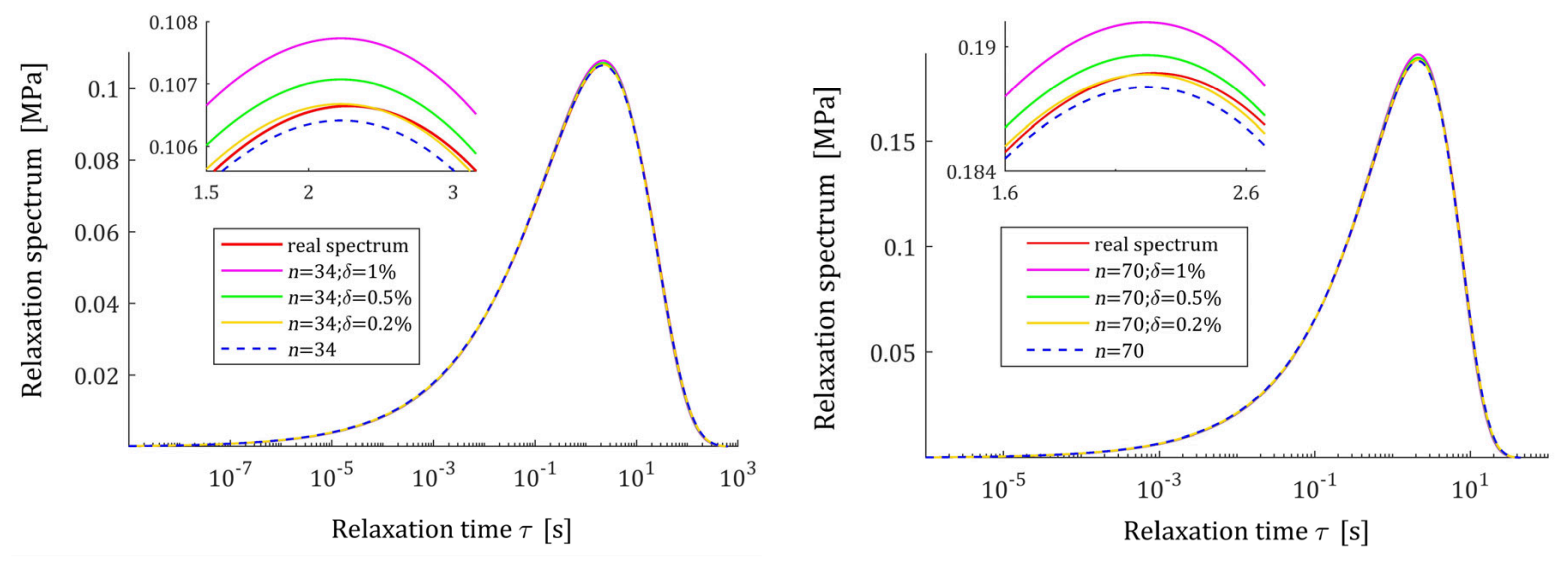
The KWW spectrum $H\left(\tau \right)$ (7) (solid red line) of the parameters: $G_{0}=0.78\;\mathrm{MPa}$, $\tau_{r}=1.08\;s$ and the original $H_{n}\left(\tau \right)$ (32) and perturbed ${\bar{H}}_{n}\left(\tau \right)$ models determined for 0.2%, 0.5% and 1% perturbations of the stretching exponents: (**a**) $\beta =\frac{1}{3}$, (**b**) $\beta =\frac{1}{2}$.

**Figure 11:**
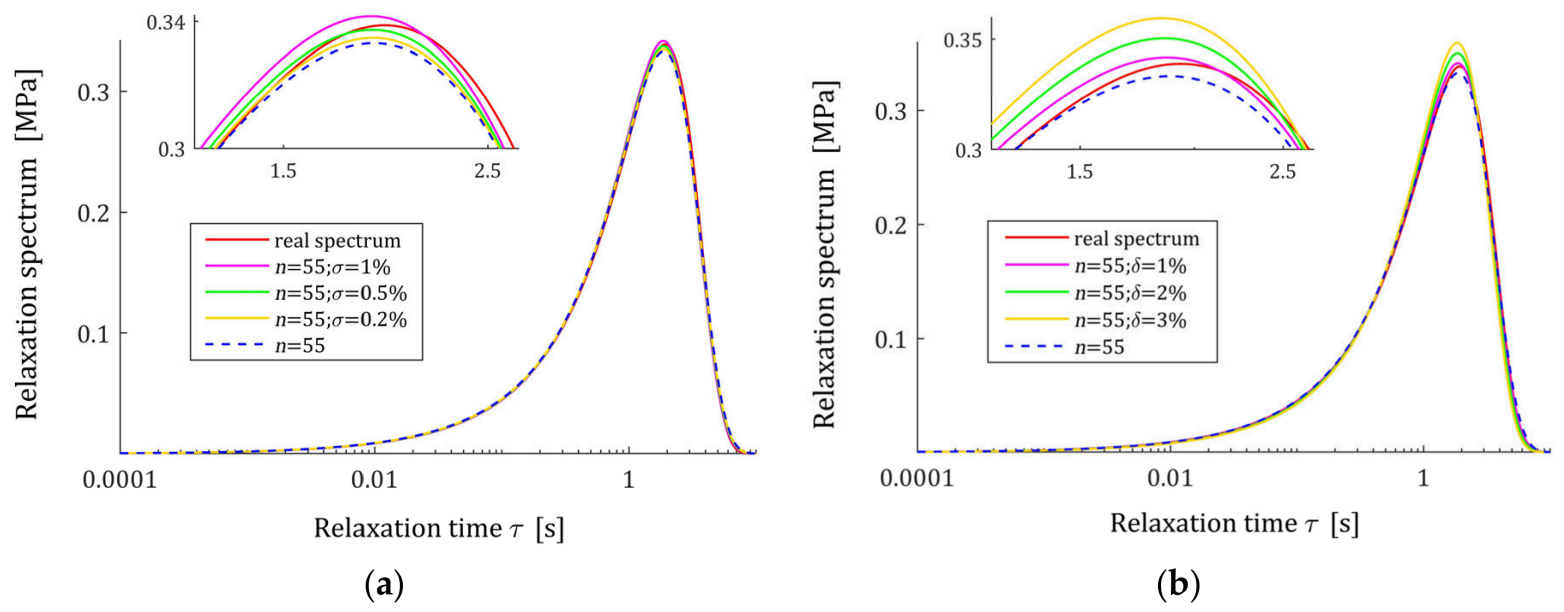
The KWW spectrum $H\left(\tau \right)$ (7) (solid red line) of the parameters: $G_{0}=0.78\;\mathrm{MPa}$, $\tau_{r}=1.08\;s$, and $\beta =\frac{2}{3}$ and the original $H_{n}\left(\tau \right)$ (32) and perturbed ${\bar{H}}_{n}\left(\tau \right)$ models determined for: (**a**) 0.2%, 0.5% and 1% perturbations, (**b**) 1%, 2% and 3% perturbations.

For numerical studies, 0.2%, 0.5% and 1% perturbations of the stretching exponents β are assumed. Other simulation parameters applied before were retained; this applies the parameters $G_{0}=0.78\;\mathrm{MPa}$ and $\tau_{r}=1.08\;s$, the relaxation times intervals T and the number of sampling points N=50000. The perturbed model ${\bar{H}}_{n}\left(\tau \right)$ was compared with the original non-perturbed model $H_{n}\left(\tau \right)$ of the same order determined in the main studies. For $\beta =\frac{1}{3},\;\frac{1}{2},\frac{2}{3}\;$, the figures including the spectra ${\bar{H}}_{n}\left(\tau \right)$ and $H_{n}\left(\tau \right)$ are given below in Figure 10 and Figure 11; for the remaining stretching exponents these figures are provided in the Supplementary Materials as Figures S4–S6. For $\beta =\frac{2}{3}$, except perturbations 0.2%, 0.5% and 1%, strong perturbations 2% and 3% were applied; the respective models are depicted in Figure 11b.

However, according to the Post–Widder convergence property described by Equation (33), the sequence of perturbed models ${\bar{H}}_{n}\left(\tau \right)$ converges to the perturbed model $\bar{H}\left(\tau \right)$ defined by respective parameters; here, non-perturbed $G_{0}=0.78\;\mathrm{MPa}$, $\tau_{r}=1.08\;s$ and perturbed stretching exponent β. Therefore, the question of the influence of the model parameter perturbation on the models $H_{n}\left(\tau \right)$ and ${\bar{H}}_{n}\left(\tau \right)$ discrepancy, is, in fact, the question of the KWW spectrum $H\left(\tau \right)$ sensitivity on its parameter perturbation, because, as previously shown, the Algorithm provides an arbitrarily accurate approximation of $H\left(\tau \right)$ by the model $H_{n}\left(\tau \right)$ if a sufficiently small accuracy ε is assumed. The above studies illustrate that for the stretching exponent perturbations not greater than 1%, the discrepancy between models $H_{n}\left(\tau \right)$ and ${\bar{H}}_{n}\left(\tau \right)$ is acceptably small.

## 5. Discussion

Both the indices applied, $J\left(n\right)$ and $Q\left(n\right)$, monotonically decrease with the increasing order of the Post–Widder approximation; however, the stretching exponent β noticeably affects their values and convergence speeds. For example, the mean relative index $Q\left(n\right)$ of the real spectrum $H\left(\tau \right)$ approximation takes a satisfactory value, i.e., drops below 0.5%, already for the model order n=19 for $\beta =\frac{1}{3}$ and for n=30 for $\beta =\frac{2}{3}$, while for $\beta =\frac{1}{2}$ the model order n=38 it is necessary to achieve a comparable approximation accuracy. In turn, index $J\left(n\right)$ of the discrepancy between the successive models does not exceed 10^−4^% for the smallest β=0.1 already for n=8, for $\beta =\frac{1}{3}$ when n≥34, for $\beta =\frac{2}{3}$ when n≥120, and for β=0.85 when n≥124, while for $\beta =\frac{1}{2}$ the biggest model order n=139 is necessary. In the neighborhood of $\beta =\frac{1}{2}$ the order of the model guaranteeing the assumed accuracy level is the highest. Thus, the method ensures that the faster the convergence of the generated sequence of models, the smaller the stretching exponent is. Simultaneously, for the smaller stretching exponents the accuracy of the final model is better when the same level of the discrepancy of subsequent models is assumed in the Algorithm switching and stopping rules.

The results of the numerical studies provide practical guidance on how to select, for a given stretching exponent β, the relaxation times interval T and the level of the successive model discrepancy ε to obtain an accurate fit of the relaxation spectrum model to the real spectrum over the entire range of relaxation times of a given process. The data from Table A3 suggest how to select the relaxation times interval T. The width of the relaxation time intervals T decreases exponentially with the increase in the stretching exponent β. For large β=0.85 it is 13.67 times smaller than for small β=0.1. The lower bound $\tau_{min}$ of the interval T exponentially increases, while the upper bound $\tau_{max}$ exponentially decreases with an increasing parameter β. The selection of an appropriate T affects the values of the index $J\left(n\right)$ (40), whence also the number of the scheme iterations depends on T. A too-wide time interval T overestimates the value of the index $J\left(n\right)$ due to the division by values close to zero at the ends of the time interval.

The efficiency of the Algorithm, for a given β, also depends on the selection of the model discrepancy accuracy ε. A too-large ε means only a few iterations of the algorithm and a low model order, but it does not ensure its accuracy. In turn, a too-small ε implies an accurate model, but the number of iterations and, most importantly, the order of the model may be impractically large. Inspection of the data from Table A2, Table A3 and Table A4, Table S1–S12, and in particular the results summarized in Table A4, leads to the conclusion that for stretching exponents greater than 0.2, to ensure an excellent accuracy of the real spectrum approximation, it is sufficient to assume the level ε of the successive model discrepancy of the order of 10^−3^–10^−4^%; however, for β≤0.2 it is advisable to assume this level of the order of 10^−5^%. For all the stretching exponents, the determined models perfectly reproduce the maximum of the real characteristics; the respective relative approximation errors drop below 0.14%; however, they are mostly of the order 10^−2^–10^−4^%.

However, the KWW model generally describes stretched exponential behavior with stretching exponent in the range 0<β<1; the relaxation in some materials can be accurately described using compressed exponential functions, with parameter β greater than one. For example, Sharma [14], studying transitory relaxation behavior in various Ge-Se glass compositions, observed exponential compressed relaxation characterized by parameters 2.7<β<3.5. The assumption that β <1 is not actively used to prove the validity of the main theorems of this paper, i.e., Theorems 1–4 also hold for β >1. However, the results concerning the mathematical properties of the KWW spectrum models, i.e., its monotonicity and extremal properties were derived under the assumption that β <1. Therefore, the application of the proposed approach to modeling the spectrum of the compressed exponential processes requires verification of these properties assuming that β >1.

## 6. Conclusions

The exact formulas describing the KWW relaxation spectrum are known in the literature, but they have inconvenient infinite power series or complex integral forms. The relaxation spectrum is uniquely determined by the inverse Laplace transform of the relaxation modulus from the positive real axis according to the Post–Widder inverse Laplace rule that, generally, needs the values of the relaxation modulus and its derivatives of the high order to compute the spectrum. In this paper the main result concerns the designation of the analytical formulas describing the n-th order Post–Widder approximate inversion of the KWW relaxation modulus for the relaxation spectrum modeling. The specific properties of the KWW relaxation function were used to derive a formula that describes the high-order Post–Widder approximation of the relaxation time spectrum directly in terms of the relaxation modulus, without any—either analytical or numerical—differentiation of the modulus. The derived alternative recursive formula describing the approximate model of the KWW spectrum also has a simple algebraic form, which does not require the differentiation inherent in the Post–Widder inversion rule. Therefore, the stretched exponential form of the KWW model is consequential here.

As a result, a sequence of new models of the KWW spectrum was derived. The complete computational algorithm of the spectrum model determination is presented. When determining the KWW relaxation spectrum using the algorithm, the basic question is: “how large should the order of the Post–Widder approximation be?” The answer to this question is provided by selecting the level of the discrepancy between the successive models. The order of the determined model increases with the stretching exponent; however, the adaptive switching rule applied by the algorithm for the selection of the successively iterated models ensures that there is no significant increase in the number of iterations necessary to determine them. Extensive numerical studies performed for the KWW spectra of nine stretching exponents, covering relaxation times from 3 to 41 decades, demonstrated that the spectrum model reproduces the real KWW spectrum very accurately when the order of the Post–Widder approximation was from 15 to 62, depending on the stretching exponent. Based on the analysis of the influence of the stretching exponent on the scheme effectiveness, the guidelines were formulated for selecting the level of the discrepancy between the subsequent models for the algorithm’s switching and stopping rules.

In summary, it has been proven that an arbitrarily accurate model of the KWW relaxation time spectrum can be recovered directly from the KWW relaxation modulus without any modulus differentiation. This model is given by the product of the modulus and a finite power series of the relaxation times defined by real positive double-indexed coefficients introduced by the simple recurrence rule.

Since, due to the Post–Widder theorem, the Algorithm provides an arbitrarily close approximation of $H\left(\tau \right)$ by the model $H_{n}\left(\tau \right)$ whenever a sufficiently small accuracy ε is assumed, the proposed method for KWW spectrum determination guarantees a better approximation of the real relaxation spectrum than other known relaxation spectrum identification methods whenever the relaxation processes in the material under consideration are adequately modeled by the KWW function. This property is natural, as the method proposed here addresses only the materials of the stretched exponential type and is based on its specificity. Other known methods of the relaxation spectra identification have a wider range of applicability, sometimes very wide, but they do not produce such good results for the KWW model.

Although the paper considers the rheological stretched relaxation process, the proposed approach can also be applied to modeling dielectric relaxation processes and other processes described by stretched exponential functions. The extension of the proposed approach to modeling the spectrum of the compressed exponential relaxation processes, when β >1, may be the subject of further research.

## Figures and Tables

**Table 1:** The processes of determining the approximate models $H_{n}\left(\tau \right)$ (32) of the real spectrum $H\left(\tau \right)$ (7) of the parameters $G_{0}=0.78\;\mathrm{MPa}$, $\beta =\frac{1}{3}$, and $\tau_{r}=1.08\;s$ by using the Algorithm for pre-assumed accuracies ε of the successive models $H_{n}\left(\tau \right)$ and $H_{n+1}\left(\tau \right)$ discrepancy; n—order of the Post–Widder approximation, $J\left(n\right)$ (40)—mean relative index of $H_{n}\left(\tau \right)$ and $H_{n+1}\left(\tau \right)$ discrepancy.

$\varepsilon =10^{-3}\%$		$\varepsilon =5\times 10^{-4}\%$		$\varepsilon =10^{-4}\%$		$\varepsilon =5\times 10^{-5}\%$	
n	$J\left(n\right)$ [%]	n	$J\left(n\right)$ [%]	n	$J\left(n\right)$ [%]	n	$J\left(n\right)$ [%]
1	2.192060	1	2.192060	1	2.192060	1	2.192060
11	4.17393 × 10^−3^	11	4.17393 × 10^−3^	11	4.17393 × 10^−3^	11	4.17393 × 10^−3^
14	1.93405 × 10^−3^	16	1.24671 × 10^−3^	21	4.96499 × 10^−4^	21	4.96499 × 10^−4^
16	1.24671 × 10^−3^	18	8.40224 × 10^−4^	24	3.12196 × 10^−4^	26	2.35593 × 10^−4^
17	1.01840 × 10^−3^	20	5.87122 × 10^−4^	27	2.06134 × 10^−4^	29	1.59839 × 10^−4^
18	8.40224 × 10^−4^	21	4.96499 × 10^−4^	29	1.59839 × 10^−4^	32	1.12264 × 10^−4^
				31	1.25859 × 10^−4^	34	9.01668 × 10^−5^
				33	1.00458 × 10^−4^	36	7.32545 × 10^−5^
				34	9.01668 × 10^−5^	38	6.01318 × 10^−5^
						39	5.46744 × 10^−5^
						40	4.98229 × 10^−5^

## Data Availability

The original contributions presented in this study are included in the article and Supplementary Materials. Further inquiries can be directed to the author.

## Supplementary Materials

The following supporting information can be downloaded at: https://www.mdpi.com/article/10.3390/ma19153256/s1, S1. The coefficients $a\left(n,k;\beta \right)$; Table S1: For the real relaxation spectrum $H\left(\tau \right)$ (7) of the parameters $G_{0}=0.78\;\mathrm{MPa}$, β=1/3, $\tau_{r}=1.08\;s$ and its Post–Widder n-the order approximate models $H_{n}\left(\tau \right)$ (32) the quality indices: the mean relative index $J\left(n\right)$ (40) of the discrepancy between the models $H_{n}\left(\tau \right)$ and $H_{n+1}\left(\tau \right)$, the mean relative index $Q\left(n\right)$ (39) of the real spectrum $H\left(\tau \right)$ approximation, the relaxation times $\tau_{n}^{*}$ and the spectra models maxima $H_{n}\left(\tau_{n}^{*}\right)$ defined in Equation (35) with the square relative errors $ERR\tau^{*}\left(n\right)$ (55) and $ERRH^{*}\left(n\right)$ (56) of the real spectrum maximum $\left(\tau^{*},H\left(\tau^{*}\right)\right)$ approximation; $\tau^{*}=2.238448\;s$, $H\left(\tau^{*}\right)=0.106647\;\mathrm{MPa}$; Table S2: For the real relaxation spectrum $H\left(\tau \right)$ (6) of the parameters $G_{0}=0.78\;\mathrm{MPa}$, β=0.5, $\tau_{r}=1.08\;s$ and its Post–Widder n-the order approximate models $H_{n}\left(\tau \right)$ (32) the quality indices: the mean relative index $J\left(n\right)$ (40) of the discrepancy between the models $H_{n}\left(\tau \right)$ and $H_{n+1}\left(\tau \right)$, the mean relative index $Q\left(n\right)$ (39) of the real spectrum $H\left(\tau \right)$ approximation, the relaxation times $\tau_{n}^{*}$ and the spectra models maxima $H_{n}\left(\tau_{n}^{*}\right)$ defined in Equation (35) with the square relative errors $ERR\tau^{*}\left(n\right)$ (55) and $ERRH^{*}\left(n\right)$ (56) of the real spectrum maximum $\left(\tau^{*},H\left(\tau^{*}\right)\right)$ approximation; $\tau^{*}=2.16\;s$, $H\left(\tau^{*}\right)=0.188737\;\mathrm{MPa}$; Table S3: For the real relaxation spectrum $H\left(\tau \right)$ (8) of the parameters $G_{0}=0.78\;\mathrm{MPa}$, β=2/3, $\tau_{r}=1.08\;s$ and its Post–Widder n-the order approximate models $H_{n}\left(\tau \right)$ (32) the quality indices: the mean relative index $J\left(n\right)$ (40) of the discrepancy between the models $H_{n}\left(\tau \right)$ and $H_{n+1}\left(\tau \right)$, the mean relative index $Q\left(n\right)$ (39) of the real spectrum $H\left(\tau \right)$ approximation, the relaxation times $\tau_{n}^{*}$ and the spectra models maxima $H_{n}\left(\tau_{n}^{*}\right)$ defined in Equation (35) with the square relative errors $ERR\tau^{*}\left(n\right)$ (55) and $ERRH^{*}\left(n\right)$ (56) of the real spectrum maximum $\left(\tau^{*},H\left(\tau^{*}\right)\right)$ approximation; $\tau^{*}=1.931459\;s$, $H\left(\tau^{*}\right)=0.338774\;\mathrm{MPa}$; Table S4: For the real relaxation spectrum $H\left(\tau \right)$ (4) of the parameters $G_{0}=0.78\;\mathrm{MPa}$, $\tau_{r}=1.08\;s$, β=0.1 and β=0.2 and its Post–Widder n-the order approximate models $H_{n}\left(\tau \right)$ (32) the quality indices: the mean relative index $J\left(n\right)$ (40) of the discrepancy between the models $H_{n}\left(\tau \right)$ and $H_{n+1}\left(\tau \right)$, the relaxation times $\tau_{n}^{*}$ and the spectra models maxima $H_{n}\left(\tau_{n}^{*}\right)$ defined in Equation (35); Table S5: The processes of determining the approximate model $H_{n}\left(\tau \right)$ of the real relaxation spectrum $H\left(\tau \right)$ (4) with the parameters $G_{0}=0.78\;\mathrm{MPa}$, β=0.1, and $\tau_{r}=1.08\;s$ by using the Algorithm for pre-assumed accuracies ε of the successive models $H_{n}\left(\tau \right)$ and $H_{n+1}\left(\tau \right)$ discrepancy; n—order of the Post–Widder approximation, $J\left(n\right)$ (40)—mean relative index of $H_{n}\left(\tau \right)$ and $H_{n+1}\left(\tau \right)$ discrepancy; Table S6: The processes of determining the approximate model $H_{n}\left(\tau \right)$ of the real relaxation spectrum $H\left(\tau \right)$ (4) with the parameters $G_{0}=0.78\;\mathrm{MPa}$, $\tau_{r}=1.08\;s$, and β=0.2 by using the Algorithm for pre-assumed accuracies ε of the successive models $H_{n}\left(\tau \right)$ and $H_{n+1}\left(\tau \right)$ discrepancy; n—order of the Post–Widder approximation, $J\left(n\right)$ (40)—mean relative index of $H_{n}\left(\tau \right)$ and $H_{n+1}\left(\tau \right)$ discrepancy; Table S7: For the real relaxation spectrum $H\left(\tau \right)$ (4) of the parameters $G_{0}=0.78\;\mathrm{MPa}$, $\tau_{r}=1.08\;s$, β=0.45, and β=0.55 and its Post–Widder n-the order approximate models $H_{n}\left(\tau \right)$ (32) the quality indices: the mean relative index $J\left(n\right)$ (40) of the models $H_{n}\left(\tau \right)$ and $H_{n+1}\left(\tau \right)$ discrepancy, the relaxation times $\tau_{n}^{*}$ and the spectra models maxima $H_{n}\left(\tau_{n}^{*}\right)$ defined in Equation (35); Table S8: The processes of determining the approximate model $H_{n}\left(\tau \right)$ of the real relaxation spectrum $H\left(\tau \right)$ (4) with the parameters $G_{0}=0.78\;\mathrm{MPa}$, $\tau_{r}=1.08\;s$, and β=0.45 by using the Algorithm for pre-assumed accuracies ε of the successive models $H_{n}\left(\tau \right)$ and $H_{n+1}\left(\tau \right)$ discrepancy; n—order of the Post–Widder approximation, $J\left(n\right)$ (40)—mean relative index of s $H_{n}\left(\tau \right)$ and $H_{n+1}\left(\tau \right)$ discrepancy; Table S9: The processes of determining the approximate model $H_{n}\left(\tau \right)$ of the real relaxation spectrum $H\left(\tau \right)$ (4) with the parameters $G_{0}=0.78\;\mathrm{MPa}$, $\tau_{r}=1.08\;s$, and β=0.55 by using the Algorithm for pre-assumed accuracies ε of the successive models $H_{n}\left(\tau \right)$ and $H_{n+1}\left(\tau \right)$ discrepancy; n—order of the Post–Widder approximation, $J\left(n\right)$ (40)—mean relative index of $H_{n}\left(\tau \right)$ and $H_{n+1}\left(\tau \right)$ discrepancy; Table S10: For the real relaxation spectrum $H\left(\tau \right)$ (4) of the parameters $G_{0}=0.78\;\mathrm{MPa}$, $\tau_{r}=1.08\;s$, β=0.75, and β=0.85 and its Post–Widder n-the order approximate models $H_{n}\left(\tau \right)$ (32) the quality indices: the mean relative index $J\left(n\right)$ (40) of the discrepancy between the models $H_{n}\left(\tau \right)$ and $H_{n+1}\left(\tau \right)$, the relaxation times $\tau_{n}^{*}$ and the spectra models maxima $H_{n}\left(\tau_{n}^{*}\right)$ defined in Equation (35); Table S11: The process of determining the approximate model $H_{n}\left(\tau \right)$ of the real relaxation spectrum $H\left(\tau \right)$ (4) with the parameters $G_{0}=0.78\;\mathrm{MPa}$, β=0.75, and $\tau_{r}=1.08\;s$ by using the Algorithm for pre-assumed accuracies ε of the successive models $H_{n}\left(\tau \right)$ and $H_{n+1}\left(\tau \right)$ discrepancy; n—order of the Post–Widder approximation, $J\left(n\right)$ (40)—mean relative index of $H_{n}\left(\tau \right)$ and $H_{n+1}\left(\tau \right)$ discrepancy; Table S12: The processes of determining the approximate model $H_{n}\left(\tau \right)$ of the real relaxation spectrum $H\left(\tau \right)$ (4) with the parameters $G_{0}=0.78\;\mathrm{MPa}$, β=0.85, and $\tau_{r}=1.08\;s$ by using the Algorithm for pre-assumed accuracies ε of the successive models $H_{n}\left(\tau \right)$ and $H_{n+1}\left(\tau \right)$ discrepancy; n—order of the Post–Widder approximation, $J\left(n\right)$ (40)—mean relative index of $H_{n}\left(\tau \right)$ and $H_{n+1}\left(\tau \right)$ discrepancy; Figure S1: For the KWW spectrum $H\left(\tau \right)$ (4) of the parameters $G_{0}=0.78\;\mathrm{MPa}$, β=0.55, and $\tau_{r}=1.08\;s$, the approximate models $H_{n}\left(\tau \right)$ (32) of the order n iterated by the Algorithm for the accuracies ε of the successive models $H_{n}\left(\tau \right)$ and $H_{n+1}\left(\tau \right)$ discrepancy; (a) $\varepsilon =5\times 10^{-4}\%$ and (b) $\varepsilon =10^{-4}\%$; Figure S2: For the KWW spectrum $H\left(\tau \right)$ (4) of the parameters $G_{0}=0.78\;\mathrm{MPa}$, β=0.75, and $\tau_{r}=1.08\;s$, the approximate models $H_{n}\left(\tau \right)$ (32) of the order n iterated by the Algorithm for the accuracies ε of the successive models $H_{n}\left(\tau \right)$ and $H_{n+1}\left(\tau \right)$ discrepancy; (a) $\varepsilon =5\times 10^{-4}\%$ and (b) $\varepsilon =10^{-4}\%$; Figure S3: For the KWW spectrum $H\left(\tau \right)$ (4) of the parameters $G_{0}=0.78\;\mathrm{MPa}$, β=0.85, and $\tau_{r}=1.08\;s$, the approximate models $H_{n}\left(\tau \right)$ (32) of the order n iterated by the Algorithm for the accuracies ε of the successive models $H_{n}\left(\tau \right)$ and $H_{n+1}\left(\tau \right)$ discrepancy; (a) $\varepsilon =5\times 10^{-4}\%$ and (b) $\varepsilon =10^{-4}\%$; Figure S4: The KWW spectrum $H\left(\tau \right)$ (7) (solid red line) of the parameters: $G_{0}=0.78\;\mathrm{MPa}$, $\tau_{r}=1.08\;s$ and the original $H_{n}\left(\tau \right)$ (32) and perturbed ${\bar{H}}_{n}\left(\tau \right)$ models determined for 0.2%, 0.5% and 1% perturbations of the stretching exponents: (a) β=0.1, (b) β=0.2; Figure S5: The KWW spectrum $H\left(\tau \right)$ (7) (solid red line) of the parameters: $G_{0}=0.78\;\mathrm{MPa}$, $\tau_{r}=1.08\;s$ and the original $H_{n}\left(\tau \right)$ (32) and perturbed ${\bar{H}}_{n}\left(\tau \right)$ models determined for 0.2%, 0.5% and 1% perturbations of the stretching exponents: (a) β=0.45, (b) β=0.55; Figure S6: The KWW spectrum $H\left(\tau \right)$ (7) (solid red line) of the parameters: $G_{0}=0.78\;\mathrm{MPa}$, $\tau_{r}=1.08\;s$ and the original $H_{n}\left(\tau \right)$ (32) and perturbed ${\bar{H}}_{n}\left(\tau \right)$ models determined for 0.2%, 0.5% and 1% perturbations of the stretching exponents: (a) β=0.75, (b) β=0.85.

## Appendix A

### Appendix A.1. Proof of Theorem 1

Mathematical induction is used. In the base case of the proof by induction, for n=1, Equation (15) reduces to

$$\frac{dG\left(t\right)}{dt}=-\tau_{r}^{-\beta }\left(\begin{array}{c} 0\\ 0 \end{array}\right)\beta G\left(t\right)t^{\beta -1},$$

and, in view of $\left(\begin{array}{c} 0\\ 0 \end{array}\right)=1$, is equivalent to (14).

Via the induction hypothesis, the n-th order derivative $\frac{d^{n}G\left(t\right)}{dt^{n}}$ is given by (15). By two-sided differentiation of (15) with respect to t we obtain

$$\frac{d^{n+1}G\left(t\right)}{dt^{n+1}}=-\tau_{r}^{-\beta }\sum_{k=1}^{n}\left(\begin{array}{c} n-1\\ k-1 \end{array}\right){\left(\beta \right)}_{k}\frac{d^{n-k+1}G\left(t\right)}{dt^{n-k+1}}t^{\beta -k}-\tau_{r}^{-\beta }\sum_{k=1}^{n}\left(\begin{array}{c} n-1\\ k-1 \end{array}\right){\left(\beta \right)}_{k}\left(\beta -k\right)\frac{d^{n-k}G\left(t\right)}{dt^{n-k}}t^{\beta -k-1},$$

which can be expressed as

$$\frac{d^{n+1}G\left(t\right)}{dt^{n+1}}=-\tau_{r}^{-\beta }\sum_{k=1}^{n}\left(\begin{array}{c} n-1\\ k-1 \end{array}\right){\left(\beta \right)}_{k}\frac{d^{n-k+1}G\left(t\right)}{dt^{n-k+1}}t^{\beta -k}-\tau_{r}^{-\beta }\sum_{k=1}^{n}\left(\begin{array}{c} n-1\\ k-1 \end{array}\right){\left(\beta \right)}_{k+1}\frac{d^{n+1-\left(k+1\right)}G\left(t\right)}{dt^{n+1-\left(k+1\right)}}t^{\beta -\left(k+1\right)},$$

whence, by shifting the limits of summation in the second sum on the right-hand side, we obtain

$$\frac{d^{n+1}G\left(t\right)}{dt^{n+1}}=-\tau_{r}^{-\beta }\sum_{k=1}^{n}\left(\begin{array}{c} n-1\\ k-1 \end{array}\right){\left(\beta \right)}_{k}\frac{d^{n-k+1}G\left(t\right)}{dt^{n-k+1}}t^{\beta -k}-\tau_{r}^{-\beta }\sum_{k=2}^{n+1}\left(\begin{array}{c} n-1\\ k-2 \end{array}\right){\left(\beta \right)}_{k}\frac{d^{n+1-k}G\left(t\right)}{dt^{n+1-k}}t^{\beta -k},$$

and next, after merging the sums for k from 2 to n, we can represent this equation in the form

(A1) $$\begin{array}{c} \frac{d^{n+1}G\left(t\right)}{dt^{n+1}}=-\tau_{r}^{-\beta }\left(\begin{array}{c} n-1\\ 0 \end{array}\right)\beta \frac{d^{n}G\left(t\right)}{dt^{n}}t^{\beta -1}-\tau_{r}^{-\beta }\sum_{k=2}^{n}\left[\left(\begin{array}{c} n-1\\ k-1 \end{array}\right)+\left(\begin{array}{c} n-1\\ k-2 \end{array}\right)\right]{\left(\beta \right)}_{k}\frac{d^{n-k+1}G\left(t\right)}{dt^{n-k+1}}t^{\beta -k}-\\ \tau_{r}^{-\beta }\left(\begin{array}{c} n-1\\ n-1 \end{array}\right){\left(\beta \right)}_{n+1}G\left(t\right)t^{\beta -n-1}. \end{array}$$

Since

$$\left(\begin{array}{c} n-1\\ 0 \end{array}\right)=1=\left(\begin{array}{c} n\\ 0 \end{array}\right)\;\mathrm{and}\;\left(\begin{array}{c} n-1\\ n-1 \end{array}\right)=1=\left(\begin{array}{c} n\\ n \end{array}\right),$$

using the known recursive formula of the binomial coefficient (Pascal’s identity) [87]:

$$\left(\begin{array}{c} n\\ k-1 \end{array}\right)=\left(\begin{array}{c} n-1\\ k-1 \end{array}\right)+\left(\begin{array}{c} n-1\\ k-2 \end{array}\right),$$

we can express the right-hand side of (A1) in a compact form as follows

$$\frac{d^{n+1}G\left(t\right)}{dt^{n+1}}=-\tau_{r}^{-\beta }\sum_{k=1}^{n+1}\left(\begin{array}{c} n\\ k-1 \end{array}\right){\left(\beta \right)}_{k}\frac{d^{n+1-k}G\left(t\right)}{dt^{n-k+1}}t^{\beta -k},$$

which is the rule (15) for n+1. The theorem is proved.

### Appendix A.2. Proof of Theorem 3

Mathematical induction is used. In the base case, for n=1, the first derivative computed according to (24) is as follows

$$\frac{dG\left(t\right)}{dt}=-\left(\frac{1}{\tau_{r}}\right)a\left(1,1;\beta \right){\left(\frac{t}{\tau_{r}}\right)}^{\left(\beta -1\right)}G\left(t\right),$$

and, since in view of (26) $a\left(1,1;\beta \right)=\beta$, this formula is identical with that given by (14).

Let us express Equation (24) equivalently as follows

(A2) $$\frac{d^{n}G\left(t\right)}{dt^{n}}={\left(-1\right)}^{n}{\left(\frac{1}{\tau_{r}}\right)}^{n}\left[\sum_{k=1}^{n}a\left(n,k;\beta \right){\left(\frac{t}{\tau_{r}}\right)}^{\left(n-k+1\right)\beta -n}\right]G\left(t\right),$$

Via the induction hypothesis, the n-th order derivative $\frac{d^{n}G\left(t\right)}{dt^{n}}$ is given by Equation (A2). By two-sided differentiation of (A2), including (14), we obtain

$$\begin{array}{c} \frac{d^{n+1}G\left(t\right)}{dt^{n+1}}={\left(-1\right)}^{n}{\left(\frac{1}{\tau_{r}}\right)}^{n+1}\left[\sum_{k=1}^{n}a\left(n,k;\beta \right)\left[\left(n-k+1\right)\beta -n\right]{\left(\frac{t}{\tau_{r}}\right)}^{\left(n-k+1\right)\beta -n-1}\right]G\left(t\right)-\\ {\left(-1\right)}^{n}{\left(\frac{1}{\tau_{r}}\right)}^{n+1}\left[\sum_{k=1}^{n}\beta a\left(n,k;\beta \right){\left(\frac{t}{\tau_{r}}\right)}^{\left(n-k+2\right)\beta -n-1}\right]G\left(t\right), \end{array}$$

whence, by shifting the index k in the first sum by one according to the scheme k+1→k, we have

$$\begin{array}{c} \frac{d^{n+1}G\left(t\right)}{dt^{n+1}}={\left(-1\right)}^{n}{\left(\frac{1}{\tau_{r}}\right)}^{n+1}\left[\sum_{k=2}^{n+1}a\left(n,k-1;\beta \right)\left[\left(n-k+2\right)\beta -n\right]{\left(\frac{t}{\tau_{r}}\right)}^{\left(n-k+2\right)\beta -n-1}\right]G\left(t\right)-\\ {\left(-1\right)}^{n}{\left(\frac{1}{\tau_{r}}\right)}^{n+1}\left[\sum_{k=1}^{n}\beta a\left(n,k;\beta \right){\left(\frac{t}{\tau_{r}}\right)}^{\left(n-k+2\right)\beta -n-1}\right]G\left(t\right). \end{array}$$

By splitting the sums into two terms and merging the two sums from 2 to n, the above equation can be expressed as

(A3) $$\begin{array}{c} \frac{d^{n+1}G\left(t\right)}{dt^{n+1}}={\left(-1\right)}^{n}{\left(\frac{1}{\tau_{r}}\right)}^{n+1}a\left(n,n;\beta \right)\left(\beta -n\right){\left(\frac{t}{\tau_{r}}\right)}^{\beta -n-1}G\left(t\right)+{\left(-1\right)}^{n}{\left(\frac{1}{\tau_{r}}\right)}^{n+1}\{\sum_{k=2}^{n}[a(n,k-\\ 1;\beta )\left[\left(n-k+2\right)\beta -n\right]-\beta a\left(n,k;\beta \right)]{\left(\frac{t}{\tau_{r}}\right)}^{-k\beta }\}{\left(\frac{t}{\tau_{r}}\right)}^{\left(n+2\right)\beta -n-1}G\left(t\right)-\\ {\left(-1\right)}^{n}{\left(\frac{1}{\tau_{r}}\right)}^{n+1}\beta a\left(n,1;\beta \right){\left(\frac{t}{\tau_{r}}\right)}^{\left(n+1\right)\beta -n-1}G\left(t\right). \end{array}$$

Having in mind the failing factorial identity

(A4) $${\left(p\right)}_{i+1}={\left(p\right)}_{i}\left(p-i\right),$$

in view of (27), we have

(A5) $$a\left(n,n;\beta \right)\left(\beta -n\right)={\left(-1\right)}^{n+1}{\left(\beta \right)}_{n}\left(\beta -n\right)={\left(-1\right)}^{n+1}{\left(\beta \right)}_{n+1}=-a\left(n+1,n+1;\beta \right).$$

In view of (26), we have

(A6) $$\beta a\left(n,1;\beta \right)=a\left(n+1,1;\beta \right).$$

Thus, applying (A5) and (25), Equation (A3) takes the form

$$\begin{array}{c} \frac{d^{n+1}G\left(t\right)}{dt^{n+1}}={\left(-1\right)}^{n+1}{\left(\frac{1}{\tau_{r}}\right)}^{n+1}a\left(n+1,n+1;\beta \right){\left(\frac{t}{\tau_{r}}\right)}^{\beta -n-1}G\left(t\right)+{\left(-1\right)}^{n+1}{\left(\frac{1}{\tau_{r}}\right)}^{n+1}\sum_{k=2}^{n}a(n+\\ 1,k){\left(\frac{t}{\tau_{r}}\right)}^{-k\beta }{\left(\frac{t}{\tau_{r}}\right)}^{\left(n+2\right)\beta -n-1}G\left(t\right)+{\left(-1\right)}^{n+1}{\left(\frac{1}{\tau_{r}}\right)}^{n+1}a\left(n+1,1;\beta \right){\left(\frac{t}{\tau_{r}}\right)}^{\left(n+1\right)\beta -n-1}G\left(t\right), \end{array}$$

and, by grouping the components of the right hand side, can be rewritten in compact form as follows

$$\frac{d^{n+1}G\left(t\right)}{dt^{n+1}}={\left(-1\right)}^{n+1}{\left(\frac{1}{\tau_{r}}\right)}^{n+1}\sum_{k=1}^{n+1}a\left(n+1,k\right){\left(\frac{t}{\tau_{r}}\right)}^{-\left(k-1\right)\beta }{\left(\frac{t}{\tau_{r}}\right)}^{\left(n+1\right)\left(\beta -1\right)}G\left(t\right),$$

which is Equation (24) for n+1. The theorem is proved.

### Appendix A.3. Proof of Proposition 5

Mathematical induction with respect to n is used. In the base case, for n=1, the inequality (29) follows directly from the boundary condition (26). Assume, as the induction hypothesis, that the inequality (29) holds for given n≥1. Since for the 2≤k≤n inequality $n-\beta \left(n-k+2\right)>0$ is valid, according the recurrence rule (25) we have

$$a\left(n+1,k\right)=\left[n-\beta \left(n-k+2\right)\right]a\left(n,k-1\right)+\beta a\left(n,k\right)>0.$$

Thus, inequality (29) holds for n+1 and 2≤k≤n. From (26), we immediately conclude that inequality (29) holds for n+1 and k=1, while, in view of (27), the property expressed for 0<β<1 in Equation (17) implies that

$$a\left(n+1,n+1\right)={\left(-1\right)}^{n+2}{\left(\beta \right)}_{n+1}>0.$$

Thus the inequality (29) holds for any 1≤k≤n+1, which completes the proof.

### Appendix A.4. Proof of Proposition 6

The three cases (*i*)–(*iii*) will be considered separately; again mathematical induction with respect to n is used.

First, note that in the base case, for n=2, according the formulas (26) and (27):

(A7) $$a\left(2,1;\beta \right)=\beta^{2},\;\;\;a\left(2,2;\beta \right)=\beta \left(1-\beta \right).$$

In turn, for n=3, according (26), (27) and formula (30):

(A8) $$\begin{array}{c} a\left(3,1;\beta \right)=\beta^{3},\;,a\left(3,2;\beta \right)=3\left(1-\beta \right)\beta^{2},\;a\left(3,3;\beta \right)={\left(\beta \right)}_{3}=\\ \beta \left(\beta -1\right)\left(\beta -2\right). \end{array}$$

According to the recurrence rule (25), for an arbitrary n≥2, 0<β<1 and any 1≤k≤n−1 we have

(A9) $$a\left(n+1,k+1;\beta \right)=\left[n-\beta \left(n-k+1\right)\right]a\left(n,k;\beta \right)+\beta a\left(n,k+1;\beta \right),$$

which combined with (25) means that

(A10) $$\begin{array}{c} a\left(n+1,k+1;\beta \right)-a\left(n+1,k;\beta \right)=\left[n-\beta \left(n-k+2\right)\right][a\left(n,k;\beta \right)-\\ a\left(n,k-1;\beta \right)]+\beta a\left(n,k+1;\beta \right), \end{array}$$

while the last element of the sequence $\left\{a\left(n+1,k;\beta \right)\right\}$, according to (27) and failing factorial identity (A4), is as follows

(A11) $$a\left(n+1,n+1;\beta \right)={\left(-1\right)}^{n+2}{\left(\beta \right)}_{n+1}=\left(n-\beta \right)a\left(n,n;\beta \right).$$

Ad (*i*). In the base case, for *n* = 2 the monotonicity of the sequence $\left\{a\left(2,k;\beta \right)\right\}$ follows directly from (A7) since $\beta^{2}<\;\beta \left(1-\beta \right)$ for $0<\beta <\frac{1}{2}$.

Assume, as the induction hypothesis, that for given n≥2 the sequence $\left\{a\left(n,k;\beta \right)\right\}$ monotonically (strictly) increases for 1≤k≤n. Then, having in mind property (29), for any 1≤k≤n−1 we see that by (A10) the inequality

(A12) $$a\left(n+1,k+1;\beta \right)-a\left(n+1,k;\beta \right)>0$$

holds since for $0<\beta <\frac{1}{2}$ the inequality $n-\beta \left(n-k+2\right)>0$ is valid for any n≥2 and 1≤k≤n−1.

According to (A9), for k=n−1, we have

(A13) $$a\left(n+1,n;\beta \right)=\left(n-2\beta \right)a\left(n,n-1;\beta \right)+\beta a\left(n,n;\beta \right).$$

Simultaneously, according to the induction hypothesis, $a\left(n,n-1;\beta \right)<a\left(n,n;\beta \right)$, which combined with (A13) and (A11) implies

$$a\left(n+1,n;\beta \right)<\left(n-\beta \right)a\left(n,n;\beta \right)=a\left(n+1,n+1;\beta \right)$$

and proves that the sequence $\left\{a\left(n+1,k;\beta \right)\right\}$ is strictly increasing.

Ad (*ii*). Let us consider, as the base case, n=2 and n=3. For n=2, the sequence $\left\{a\left(2,k;\beta \right)\right\}$ has only two elements and, by (A7), we have $a\left(2,1;\beta \right)=a\left(2,2;\beta \right)=\frac{1}{4}$. For n=3, according to (A8) we have

$$a\left(3,1;\beta \right)={\left(\frac{1}{2}\right)}^{3}<a\left(3,2;\beta \right)=3{\left(\frac{1}{2}\right)}^{3}=a\left(3,3;\beta \right)={\left(\frac{1}{2}\right)}^{2}\left(\frac{3}{2}\right),$$

i.e., the monotonicity property (*ii*) holds for n=2 and n=3.

Assume, as the induction hypothesis, that for given n≥3 the sequence $\left\{a\left(n,k;\beta \right)\right\}$ monotonicity is as described in the case (*ii*), i.e., for any 1≤k≤n−1 inequality $a\left(n,k;\beta \right)>a\left(n,k-1;\beta \right)$ holds, while $a\left(n,n;\beta \right)=a\left(n,n-1;\beta \right)$. Thus, as previously, for 1≤k≤n−1 by (A10) the inequality (A12) holds since for $\beta =\frac{1}{2}$ the inequality $n-\frac{1}{2}\left(n-k+2\right)=\frac{1}{2}\left(n+k-2\right)>0$ is valid. For $\beta =\frac{1}{2}$ and k=n−1, including the induction hypotheses $a\left(n,n;\beta \right)=a\left(n,n-1;\beta \right)$, by Equation (A9) we obtain

$$a\left(n+1,n;\beta \right)=\left(n-1\right)a\left(n,n-1;\beta \right)+\frac{1}{2}a\left(n,n;\beta \right)=\left(n-\frac{1}{2}\right)a\left(n,n;\beta \right),$$

which, in view of (A11), yields equality $a\left(n+1,n;\beta \right)=a\left(n+1,n+1;\beta \right)$ and completes the proof of case (*ii*).

Ad (*iii*). Consider, again, as the base case n=2 and n=3. Since $\frac{1}{2}<\beta <\frac{3}{4}$, for n=2, by (A7) the inequality holds

$$a\left(2,1;\beta \right)=\beta^{2}>\beta \left(1-\beta \right)=a\left(2,2;\beta \right).$$

For n=3 and $\frac{1}{2}<\beta <\frac{3}{4}$, due to (A8), we obtain

$$a\left(3,1;\beta \right)=\beta^{3}<3\left(1-\beta \right)\beta^{2}=a\left(3,2;\beta \right)$$

and, using the formulas described in Equation (A8), we see that for any $\frac{1}{2}<\beta <\frac{3}{4}$ the inequality holds

$$a\left(3,2;\beta \right)=3\left(1-\beta \right)\beta^{2}>\beta \left(\beta -1\right)\left(\beta -2\right)=a\left(3,3;\beta \right).$$

According to the induction hypothesis, for given n≥3 the sequence $\left\{a\left(n,k;\beta \right)\right\}$ monotonically increases for any 1≤k≤n−1, while

(A14) $$a\left(n,n;\beta \right)<a\left(n,n-1;\beta \right).$$

Thus, as previously, the increasing of the sequence $\left\{a\left(n+1,k;\beta \right)\right\}$ for 1≤k≤n follows directly from Equation (A10), in which both summands of the right-hand side are positive, while for the last two elements, by (A9) and (A14), we have

$$a\left(n+1,n;\beta \right)=\left(n-2\beta \right)a\left(n,n-1;\beta \right)+\beta a\left(n,n;\beta \right)>\left(n-\beta \right)a\left(n,n;\beta \right),$$

which, in view of (A11), means that $a\left(n+1,n;\beta \right)>a\left(n+1,n+1;\beta \right)$ and completes the proof.

### Appendix A.5. Some Coefficients $a\left(n,k;\beta \right)$ for Selected β

**Table A1:** Seven first rows and columns of the matrix A composed of the coefficients $a\left(n,k;\beta \right)$ defined by Equations (25)–(27) for the stretching exponent β=0.9.

	k	1	2	3	4	5	6
n							
**1**		0.90	0	0	0	0	0
**2**		0.810	9.0 × 10^−2^	0	0	0	0
**3**		0.7290	0.2430	9.90 × 10^−2^	0	0	0
**4**		0.65610	0.437399	0.38070	0.20790	0	0
**5**		0.590490	0.656099	0.911249	1.024650	0.644489	0
**6**		0.531441	0.885735	1.738665	3.018060	3.858921	2.642409

**Table A2:** Eight first rows and columns of the matrix A composed of the coefficients $a\left(n,k;\beta \right)$ defined by Equations (25)–(27) for the stretching exponent β=0.95.

	k	1	2	3	4	5	6	7	8
n									
**1**		0.95000	0	0	0	0	0	0	0
**2**		0.902499	4.750 × 10^−2^	0	0	0	0	0	0
**3**		0.857375	0.135375	4.9875 × 10^−2^	0	0	0	0	0
**4**		0.814506	0.257213	0.196294	0.102244	0	0	0	0
**5**		0.77378	0.407253	0.482273	0.509348	0.311843	0	0	0
**6**		0.735092	0.580336	0.946863	1.520769	1.875231	1.262966	0	0
**7**		0.698337	0.771846	1.624939	3.527830	6.571892	8.888266	6.377978	0
**8**		0.663420	0.977672	2.547093	7.007554	1.7532 × 10^1^	3.5717 × 10^1^	5.1389 × 10^1^	3.8587 × 10^1^

### Appendix A.6. Proof of Theorem 7

The Formula (32), equivalent to that described by Equation (28), is used. Note that the exponents of the power functions ${\left(\frac{n\tau }{\tau_{r}}\right)}^{\left(n-k+1\right)\beta }$ in (32) are positive. Whence, for any n≥1, the zero asymptotic value when $\tau \to 0^{+}$ immediately follows, as described by the left equality in (34).

To find the limit of $H_{n}\left(\tau \right)$ as τ→∞, we determine the limit

$$\underset{\tau \to \infty }{\lim}\left[{\left(\frac{n\tau }{\tau_{r}}\right)}^{\left(n-k+1\right)\beta }G\left(n\tau \right)\right]=\underset{\tau \to \infty }{\lim}\left[G_{0}{\left(\frac{n\tau }{\tau_{r}}\right)}^{\left(n-k+1\right)\beta }e^{-{\left(\frac{n\tau }{\tau_{r}}\right)}^{\beta }}\right]$$

of the model components, c.f., (32) for an arbitrary 1≤k≤n. Using the substitution $x={\left(\frac{n\tau }{\tau_{r}}\right)}^{\beta }$, we have

(A15) $$\underset{\tau \to \infty }{\lim}\left[{\left(\frac{n\tau }{\tau_{r}}\right)}^{\left(n-k+1\right)\beta }G\left(n\tau \right)\right]=\underset{x\to \infty }{\lim}\left[G_{0}x^{n-k+1}e^{-x}\right]=\underset{x\to \infty }{\lim}\left[\frac{G_{0}x^{n-k+1}}{e^{x}}\right],$$

where both the numerator and denominator of the last limit in (A15) become infinity when x → ∞. Thus, the L’Hospital rule used $\left(n-k+1\right)$ times yields

$$\underset{\tau \to \infty }{\lim}\left[{\left(\frac{n\tau }{\tau_{r}}\right)}^{\left(n-k+1\right)\beta }G\left(n\tau \right)\right]=\underset{x\to \infty }{\lim}\left[\frac{G_{0}\left(n-k+1\right)!}{e^{x}}\right]=0,$$

which, applied to all components in (32), implies the right limit in (34) and completes the proof.

### Appendix A.7. Proof of Theorem 8

The positive definiteness of $H_{n}\left(\tau \right)$, combined with the fact that both the limits in Equation (34) vanish, means that the continuous model $H_{n}\left(\tau \right)$ has at least one local maximum. To find the stationary point equation for the maximization problem defined in (35), the differential approach is used. Based on (32) and having in mind (14), the first derivative is determined

(A16) $$\begin{array}{c} \frac{dH_{n}\left(\tau \right)}{d\tau }=\frac{\beta n}{\tau_{r}\left(n-1\right)!}\left[\sum_{k=1}^{n}a\left(n,k;\beta \right)\left(n-k+1\right){\left(\frac{n\tau }{\tau_{r}}\right)}^{\left(n-k+1\right)\beta -1}\right]G\left(n\tau \right)-\\ \frac{\beta n}{\tau_{r}\left(n-1\right)!}\left[\sum_{k=1}^{n}a\left(n,k;\beta \right){\left(\frac{n\tau }{\tau_{r}}\right)}^{\left(n-k+2\right)\beta -1}\right]G\left(n\tau \right). \end{array}$$

Shifting the summation index in the second sum by setting k−1→k, Equation (A16) takes the form

$$\begin{array}{c} \frac{dH_{n}\left(\tau \right)}{d\tau }=\frac{\beta n}{\tau_{r}\left(n-1\right)!}\left[\sum_{k=1}^{n}a\left(n,k;\beta \right)\left(n-k+1\right){\left(\frac{n\tau }{\tau_{r}}\right)}^{\left(n-k+1\right)\beta -1}\right]G\left(n\tau \right)-\frac{\beta n}{\tau_{r}\left(n-1\right)!}[\sum_{k=1}^{n-1}a(n,k+\\ 1;\beta ){\left(\frac{n\tau }{\tau_{r}}\right)}^{\left(n-k+1\right)\beta -1}]G\left(n\tau \right)-\frac{\beta n}{\tau_{r}\left(n-1\right)!}a\left(n,1;\beta \right){\left(\frac{n\tau }{\tau_{r}}\right)}^{\left(n+1\right)\beta -1}G\left(n\tau \right), \end{array}$$

which can be expressed as

(A17) $$\begin{array}{c} \frac{dH_{n}\left(\tau \right)}{d\tau }=\frac{\beta n}{\tau_{r}\left(n-1\right)!}a\left(n,n;\beta \right){\left(\frac{n\tau }{\tau_{r}}\right)}^{\beta -1}G\left(n\tau \right)-\frac{\beta n}{\tau_{r}\left(n-1\right)!}a\left(n,1;\beta \right){\left(\frac{n\tau }{\tau_{r}}\right)}^{\left(n+1\right)\beta -1}G\left(n\tau \right)+\\ \frac{n}{\tau_{r}\left(n-1\right)!}\sum_{k=1}^{n-1}\left[\beta a\left(n,k;\beta \right)\left(n-k+1\right)-\beta a\left(n,k+1;\beta \right)\right]{\left(\frac{n\tau }{\tau_{r}}\right)}^{\left(n-k+1\right)\beta -1}G\left(n\tau \right) \end{array}$$

By (A9), we have

(A18) $$\beta \left(n-k+1\right)a\left(n,k;\beta \right)-\beta a\left(n,k+1;\beta \right)=na\left(n,k;\beta \right)-a\left(n+1,k+1;\beta \right).$$

Thus, Equation (A17) can be written in a compact form as follows

$$\begin{array}{c} \frac{dH_{n}\left(\tau \right)}{d\tau }=\frac{n}{\tau_{r}\left(n-1\right)!}\left[\beta a\left(n,n;\beta \right)+\sum_{k=1}^{n-1}\left[na\left(n,k;\beta \right)-a\left(n+1,k+1;\beta \right)\right]{\left(\frac{n\tau }{\tau_{r}}\right)}^{\left(n-k\right)\beta }\right]{\left(\frac{n\tau }{\tau_{r}}\right)}^{\beta -1}G\left(n\tau \right)-\\ \frac{n}{\tau_{r}\left(n-1\right)!}\left[\beta a\left(n,1;\beta \right){\left(\frac{n\tau }{\tau_{r}}\right)}^{n\beta }\right]{\left(\frac{n\tau }{\tau_{r}}\right)}^{\beta -1}G\left(n\tau \right), \end{array}$$

whence the stationary point Equation (37) directly results.

To justify the lower and upper bounds in (36) let us return to Equation (A16), which can be written in a compact form as follows

(A19) $$\frac{dH_{n}\left(\tau \right)}{d\tau }=\frac{\beta n}{\tau_{r}\left(n-1\right)!}\sum_{k=1}^{n}\mu \left(\tau ,n,k;\beta \right),$$

where the functions

$$\mu \left(\tau ,n,k;\beta \right)=a\left(n,k;\beta \right)\left[n-k+1-{\left(\frac{n\tau }{\tau_{r}}\right)}^{\beta }\right]{\left(\frac{n\tau }{\tau_{r}}\right)}^{\left(n-k+1\right)\beta -1}G\left(n\tau \right)$$

are positive for τ>0 such that ${\left(\frac{n\tau }{\tau_{r}}\right)}^{\beta }<n-k+1$, and negative whenever ${\left(\frac{n\tau }{\tau_{r}}\right)}^{\beta }>n-k+1$.

For n=1, the sum in (A19) is composed of one element and the function

$$\mu \left(\tau ,1,1;\beta \right)=a\left(1,1;\beta \right)\left[1-{\left(\frac{\tau }{\tau_{r}}\right)}^{\beta }\right]{\left(\frac{\tau }{\tau_{r}}\right)}^{\beta -1}G\left(\tau \right)$$

has a unique root $\tau_{1}^{*}=\tau_{r}$.

For n>1 and k=1,…,n, $\frac{dH_{n}\left(\tau \right)}{d\tau }>0$ whenever ${\left(\frac{n\tau }{\tau_{r}}\right)}^{\beta }<1$ and $\frac{dH_{n}\left(\tau \right)}{d\tau }<0$ for ${\left(\frac{n\tau }{\tau_{r}}\right)}^{\beta }>n$, which is congruent with the asymptotic properties (34), and whence the lower and upper bounds in (36) immediately follows and ends the proof.

### Appendix A.8. Proof of Proposition 9

The proof begins with the derivation of the inequalities in (43). Since the quotient $\frac{H\left(\tau \right)}{H_{n+1}\left(\tau \right)}$ is a continuous function of τ and the set T is compact, the existence of the constants $m_{n+1}$ and $M_{n+1}$ immediately follows from the well-known Weierstrass’s extreme value theorem.

To prove inequality (41), it is enough to express $J\left(n\right)$ (40) as follows

$$J\left(n\right)=\frac{1}{N}\sum_{j=1}^{N}{\left[\frac{H_{n}\left(\tau_{j}\right)-H\left(\tau_{j}\right)}{H_{n+1}\left(\tau_{j}\right)}\right]}^{2}+\frac{1}{N}\sum_{j=1}^{N}{\left[\frac{H_{n+1}\left(\tau_{j}\right)-H\left(\tau_{j}\right)}{H_{n+1}\left(\tau_{j}\right)}\right]}^{2}+\frac{2}{N}\sum_{j=1}^{N}\frac{H_{n}\left(\tau_{j}\right)-H\left(\tau_{j}\right)}{H_{n+1}\left(\tau_{j}\right)}\;\frac{H\left(\tau_{j}\right)-H_{n+1}\left(\tau_{j}\right)}{H_{n+1}\left(\tau_{j}\right)}$$

Thus, having in mind $Q\left(n\right)$ (39) and the upper bound in (43), we have

$$J\left(n\right)\leq M_{n+1}^{2}Q\left(n\right)+M_{n+1}^{2}Q\left(n+1\right)+\frac{2}{N}\left|\sum_{j=1}^{N}\frac{H_{n}\left(\tau_{j}\right)-H\left(\tau_{j}\right)}{H\left(\tau_{j}\right)}\;\frac{H\left(\tau_{j}\right)-H_{n+1}\left(\tau_{j}\right)}{H\left(\tau_{j}\right)}\right|M_{n+1}^{2},$$

whence, by the Schwarz inequality for series,

(A20) $$\left|\sum_{j=1}^{N}x_{j}y_{j}\right|\leq \sqrt{\sum_{j=1}^{N}x_{j}^{2}}\sqrt{\sum_{j=1}^{N}y_{j}^{2}},$$

we finally obtain inequality (41).

Index $Q\left(n\right)$ (39) can be written as

$$Q\left(n\right)=\frac{1}{N}\sum_{j=1}^{N}1+\frac{1}{N}\sum_{j=1}^{N}{\left[\frac{H_{n}\left(\tau_{j}\right)}{H\left(\tau_{j}\right)}\right]}^{2}-\frac{2}{N}\sum_{j=1}^{N}\frac{H_{n}\left(\tau_{j}\right)}{H\left(\tau_{j}\right)}$$

and, in view of the inequalities in (43), is estimated as follows

$$Q\left(n\right)\leq \frac{1}{N}\sum_{j=1}^{N}1+\frac{1}{N}\sum_{j=1}^{N}{\left[\frac{H_{n}\left(\tau_{j}\right)}{H_{n+1}\left(\tau_{j}\right)}\right]}^{2}\frac{1}{m_{n+1}^{2}}-\frac{2}{N}\sum_{j=1}^{N}\frac{H_{n}\left(\tau_{j}\right)}{H_{n+1}\left(\tau_{j}\right)}\frac{1}{M_{n+1}}$$

Having in mind (40), the above inequality can be rewritten as

$$Q\left(n\right)\leq \frac{1}{m_{n+1}^{2}}J\left(n\right)+\frac{2}{N}\left(\frac{1}{m_{n+1}^{2}}-\frac{1}{M_{n+1}}\right)\sum_{j=1}^{N}\left(\frac{H_{n}\left(\tau_{j}\right)}{H_{n+1}\left(\tau_{j}\right)}-1\right)+\left(\frac{1}{m_{n+1}^{2}}+1-\frac{2}{M_{n+1}}\right),$$

whence the next estimation follows

$$Q\left(n\right)\leq \frac{1}{m_{n+1}^{2}}J\left(n\right)+\frac{2}{N}\left|\left(\frac{1}{m_{n+1}^{2}}-\frac{1}{M_{n+1}}\right)\sum_{j=1}^{N}\left(\frac{H_{n}\left(\tau_{j}\right)}{H_{n+1}\left(\tau_{j}\right)}-1\right)\right|+\left(\frac{1}{m_{n+1}^{2}}+1-\frac{2}{M_{n+1}}\right).$$

Applying the Schwarz inequality (A20) and bearing in mind (40), we immediately obtain the inequality (42); the proof is completed.

### Appendix A.9. Results of the Simulation Studies

**Table A3:** Relaxation times intervals $T=\left[\tau_{min},\tau_{max}\right]$ applied in the numerical studies for different stretching exponents β and the numbers L of the sampling points per one relaxation time decade when the exponential sampling rule (53) is applied.

β	$\tau_{min}\;\left[s\right]$	$\tau_{max}\;\left[s\right]$	L	No of Decades
0.1	10^−30^	10^11^	≈1219	41
0.2	10^−15^	10^5^	2500	20
$\frac{1}{3}$	10^−9^	10^3^	≈4166	12
0.45	2 × 10^−7^	2 × 10^2^	≈5555	9
0.5	10^−6^	10^2^	6250	8
0.55	10^−5^	5 × 10	≈7692	6.5
$\frac{2}{3}$	10^−4^	10	10,000	5
0.75	5 × 10^−4^	10	≈11,111	4.5
0.85	5 × 10^−3^	5	≈16,666	3

**Table A4:** For selected stretching exponents β, the Post–Widder approximate models $H_{n}\left(\tau \right)$ (32) of the real relaxation spectrum $H\left(\tau \right)$, described generally by Equation (4), of the parameters $G_{0}=0.78\;\mathrm{MPa}$, $\tau_{r}=1.08\;s$ determined by the Algorithm for pre-assumed accuracies ε of the successive models $H_{n}\left(\tau \right)$ and $H_{n+1}\left(\tau \right)$ discrepancy, the indices: I—number of the scheme iterations, n—order of the resulting Post–Widder approximation, $J\left(n\right)$ (40)—mean relative index of the models $H_{n}\left(\tau \right)$ and $H_{n+1}\left(\tau \right)$ discrepancy, the mean relative indices $Q\left(n\right)$/$\;Q_{145}\left(n\right)$ of the real spectrum $H\left(\tau \right)$/model $H_{145}\left(\tau \right)$ approximation introduced by Equations (39) and (57), errors $ERR\tau^{*}\left(n\right)$ /$ERR\tau_{145}^{*}\left(n\right)$ and $ERRH^{*}\left(n\right)$/$ERRH_{145}^{*}\left(n\right)$ of the real spectrum maximum $\left(\tau^{*},H\left(\tau^{*}\right)\right)$/maximum $\left(\tau_{145}^{*},H_{145}\left(\tau_{145}^{*}\right)\right)$ of model $H_{145}\left(\tau \right)$ approximation defined in (55)/(58) and (56)/(58), respectively.

β	ε [%]	I	n	$J\left(n\right)$ [%]	$Q\left(n\right)/Q_{145}\left(n\right)$ [%]	$ERR\tau^{*}\left(n\right)$/$ERR\tau_{145}^{*}\left(n\right)$ [%]	$ERRH^{*}\left(n\right)$**/**$ERRH_{145}^{*}\left(n\right)$ [%]
0.1	>2.59 × 10^−5^	2	11	2.58321 × 10^−5^	2.20853 × 10^−3^	0.212587	2.03285 × 10^−5^
	1 × 10^−5^	4	15	7.75445 × 10^−6^	1.11372 × 10^−3^	0.111661	1.00266 × 10^−5^
	5 × 10^−6^	4	17	4.75811 × 10^−6^	8.39594 × 10^−4^	0.088558	7.50516 × 10^−6^
	1 × 10^−6^	6	26	8.98084 × 10^−7^	3.09261 × 10^−4^	0.028392	2.71396 × 10^−6^
0.2	>1.24 × 10^−4^	2	11	1.23878 × 10^−4^	7.00852 × 10^−3^	0.261588	3.72581 × 10^−4^
	1 × 10^−5^	4	15	3.81662 × 10^−5^	3.52790 × 10^−3^	0.137337	1.83635 × 10^−4^
	5 × 10^−6^	6	26	4.56986 × 10^−6^	9.77732 × 10^−4^	0.036696	4.96584 × 10^−5^
	1 × 10^−6^	9	39	9.36608 × 10^−7^	3.43814 × 10^−4^	0.014166	1.72684 × 10^−5^
$\frac{1}{3}$	1 × 10^−3^	6	18	8.40224 × 10^−4^	0.507731	0.161979	1.73503 × 10^−3^
	5 × 10^−4^	6	21	4.96499 × 10^−4^	0.371027	0.121997	1.26373 × 10^−3^
	1 × 10^−4^	9	34	9.01668 × 10^−5^	0.139732	4.62349 × 10^−2^	4.72582 × 10^−4^
	5 × 10^−5^	11	40	4.98229 × 10^−5^	0.100631	3.33151 × 10^−2^	3.39792 × 10^−4^
0.45	1 × 10^−2^	8	25	9.47360 × 10^−3^	0.209723	8.64432 × 10^−2^	3.47436 × 10^−3^
	5 × 10^−3^	9	32	4.61930 × 10^−3^	0.114267	4.72088 × 10^−2^	1.86458 × 10^−3^
	1 × 10^−3^	14	53	9.53994 × 10^−4^	2.78762 × 10^−2^	1.14885 × 10^−2^	4.45113 × 10^−4^
	5 × 10^−4^	17	65	4.85486 × 10^−4^	1.40521 × 10^−2^	5.52459 × 10^−3^	2.22998 × 10^−4^
0.5	1 × 10^−2^	8	32	9.97527 × 10^−3^	0.692381	9.40510 × 10^−2^	6.27341 × 10^−3^
	5 × 10^−3^	12	41	4.98560 × 10^−3^	0.419602	5.86520 × 10^−2^	3.79937 × 10^−3^
	1 × 10^−3^	20	70	9.87167 × 10^−4^	0.142712	1.98813 × 10^−2^	1.29201 × 10^−3^
	5 × 10^−4^	25	87	4.89182 × 10^−4^	9.21466 × 10^−2^	1.33501 × 10^−2^	8.34356 × 10^−4^
0.55	1 × 10^−2^	6	20	8.91361 × 10^−3^	1.589096	0.218024	2.53182 × 10^−2^
	5 × 10^−3^	5	24	4.95502 × 10^−3^	1.035920	0.143104	1.64065 × 10^−2^
	1 × 10^−3^	10	39	9.67093 × 10^−4^	0.301463	4.30971 × 10^−2^	4.72563 × 10^−3^
	5 × 10^−4^	13	48	4.67251 × 10^−4^	0.166613	2.43692 × 10^−2^	2.60476 × 10^−3^
	1 × 10^−4^	19	74	9.79161 × 10^−5^	3.75204 × 10^−2^	5.441689 × 10^−3^	5.84430 × 10^−4^
$\frac{2}{3}$	1 × 10^−2^	7	21	9.68290 × 10^−3^	0.857680	0.445516	0.183409
	5 × 10^−3^	10	29	4.74719 × 10^−3^	0.500420	0.246165	9.76560 × 10^−2^
	1 × 10^−3^	18	55	9.63850 × 10^−4^	0.161413	7.30583 × 10^−2^	2.75629 × 10^−2^
	5 × 10^−4^	21	70	4.96015 × 10^−4^	0.103596	4.58481 × 10^−2^	1.70658 × 10^−2^
0.75	1 × 10^−2^	8	29	9.84399 × 10^−3^	29.994581	0.224386	0.255297
	5 × 10^−3^	11	37	4.80132 × 10^−3^	14.190156	0.125368	0.138204
	1 × 10^−3^	17	62	9.66283 × 10^−4^	2.223952	2.65876 × 10^−2^	2.80563 × 10^−2^
	5 × 10^−4^	21	76	4.99561 × 10^−4^	0.878311	1.17396 × 10^−2^	1.21003 × 10^−2^
	1 × 10^−4^	33	123	9.83562 × 10^−5^	7.44088 × 10^−3^	9.79859 × 10^−5^	1.21762 × 10^−4^
0.85	1 × 10^−2^	8	26	9.45211 × 10^−3^	16.473966	0.3617784	2.211981
	5 × 10^−3^	9	33	4.96652 × 10^−3^	10.011439	0.231609	1.368124
	1 × 10^−3^	17	59	9.61371 × 10^−4^	1.9940029	5.32604 × 10^−2^	0.293424
	5 × 10^−4^	21	74	4.90866 × 10^−4^	0.814576	2.31604 × 10^−2^	0.124209
	1 × 10^−4^	35	124	9.90552 × 10^−5^	3.42258 × 10^−3^	9.34388 × 10^−5^	5.67046 × 10^−4^

**Table A5:** The processes of the approximate model $H_{n}\left(\tau \right)$ determination by using the Algorithm for the real spectrum $H\left(\tau \right)$ (6) of the parameters $G_{0}=0.78\;\mathrm{MPa}$, β=0.5 and $\tau_{r}=1.08\;s$ for the pre-assumed accuracies ε of the successive models $H_{n}\left(\tau \right)$ and $H_{n+1}\left(\tau \right)$ discrepancy; n—order of the Post–Widder approximation, $J\left(n\right)$ (40)—mean relative index of $H_{n}\left(\tau \right)$ and $H_{n+1}\left(\tau \right)$ discrepancy.

$\varepsilon =10^{-2}\%$		$\varepsilon =5\times 10^{-3}\%$		$\varepsilon =10^{-3}\%$		$\varepsilon =5\times 10^{-4}\%$	
n	$J\left(n\right)$ [%]	n	$J\left(n\right)$ [%]	n	$J\left(n\right)$ [%]	n	$J\left(n\right)$ [%]
1	7.184564	1	7.184564	1	7.184564	1	7.184564
11	0.126433	11	0.126433	11	0.126433	11	0.126433
21	2.96787 × 10^−2^	21	2.96787 × 10^−2^	21	2.96787 × 10^−2^	21	2.96787 × 10^−2^
24	2.12655 × 10^−2^	26	1.73182 × 10^−2^	31	1.08723 × 10^−2^	31	1.08723 × 10^−2^
26	1.73182 × 10^−2^	29	1.30012 × 10^−2^	41	4.98560 × 10^−3^	41	4.98560 × 10^−3^
28	1.42678 × 10^−2^	32	9.97527 × 10^−3^	44	4.06386 × 10^−3^	46	3.56779 × 10^−3^
30	1.18756 × 10^−2^	34	8.44807 × 10^−3^	47	3.34861 × 10^−3^	51	2.62561 × 10^−3^
32	9.97527 × 10^−3^	36	7.20797 × 10^−3^	50	2.78609 × 10^−3^	56	1.97810 × 10^−3^
		38	6.19152 × 10^−3^	53	2.33835 × 10^−3^	59	1.68540 × 10^−3^
		39	5.75184 × 10^−3^	55	2.08981 × 10^−3^	62	1.44547 × 10^−3^
		40	5.35125 × 10^−3^	57	1.87385 × 10^−3^	65	1.24718 × 10^−3^
		41	4.98560 × 10^−3^	59	1.68540 × 10^−3^	67	1.13385 × 10^−3^
				61	1.52033 × 10^−3^	69	1.03324 × 10^−3^
				63	1.37521 × 10^−3^	71	9.43656 × 10^−4^
				65	1.24718 × 10^−3^	73	8.63660 × 10^−4^
				66	1.18881 × 10^−3^	75	7.92040 × 10^−4^
				67	1.13385 × 10^−3^	77	7.27756 × 10^−4^
				68	1.08207 × 10^−3^	79	6.69918 × 10^−4^
				69	1.03324 × 10^−3^	81	6.17760 × 10^−4^
				70	9.87167 × 10^−4^	82	5.93600 × 10^−4^
						83	5.70620 × 10^−4^
						84	5.48750 × 10^−4^
						85	5.27926 × 10^−4^
						86	5.08089 × 10^−4^

**Table A6:** The processes of determining the approximate model $H_{n}\left(\tau \right)$ of the real relaxation spectrum $H\left(\tau \right)$ (6) of the parameters $G_{0}=0.78\;\mathrm{MPa}$, β=2/3, and $\tau_{r}=1.08\;s$ by using the Algorithm for pre-assumed accuracies ε of the successive models $H_{n}\left(\tau \right)$ and $H_{n+1}\left(\tau \right)$ discrepancy; n—order of the Post–Widder approximation, $J\left(n\right)$ (40)—mean relative index of the models $H_{n}\left(\tau \right)$ and $H_{n+1}\left(\tau \right)$ discrepancy.

$\varepsilon =10^{-2}\%$		$\varepsilon =5\times 10^{-3}$		$\varepsilon =10^{-3}\%$		$\varepsilon =5\times 10^{-4}\;\%$	
n	$J\left(n\right)$ [%]	n	$J\left(n\right)$ [%]	n	$J\left(n\right)$ [%]	n	$J\left(n\right)$ [%]
1	11.86987	1	11.86987	1	11.86987	1	11.86987
11	3.44450 × 10^−2^	11	3.44450 × 10^−2^	11	3.44450 × 10^−2^	11	3.44450 × 10^−2^
14	2.19192 × 10^−2^	16	1.68964 × 10^−2^	21	9.68290 × 10^−3^	21	9.68290 × 10^−3^
16	1.68964 × 10^−2^	19	1.19375 × 10^−2^	26	6.08199 × 10^−3^	31	4.06662 × 10^−3^
18	1.33376 × 10^−2^	21	9.68290 × 10^−3^	31	4.06662 × 10^−3^	36	2.84714 × 10^−3^
20	1.07303 × 10^−2^	23	7.96769 × 10^−3^	34	3.26792 × 10^−3^	41	2.06559 × 10^−3^
21	9.68290 × 10^−3^	25	6.63694 × 10^−3^	37	2.66326 × 10^−3^	44	1.72792 × 10^−3^
		27	5.58719 × 10^−3^	40	2.19707 × 10^−3^	47	1.45862 × 10^−3^
		28	5.14451 × 10^−3^	42	1.94418 × 10^−3^	50	1.24131 × 10^−3^
		29	4.74719 × 10^−3^	44	1.72792 × 10^−3^	52	1.11932 × 10^−3^
				46	1.54191 × 10^−3^	54	1.01239 × 10^−3^
				48	1.38107 × 10^−3^	56	9.18275 × 10^−4^
				50	1.24131 × 10^−3^	58	8.35143 × 10^−4^
				51	1.17828 × 10^−3^	60	7.61454 × 10^−4^
				52	1.11932 × 10^−3^	62	6.95919 × 10^−4^
				53	1.06412 × 10^−3^	64	6.37457 × 10^−4^
				54	1.01239 × 10^−3^	66	5.85152 × 10^−4^
				55	9.63850 × 10^−4^	67	5.61061 × 10^−4^
						68	5.38226 × 10^−4^
						69	5.16569 × 10^−4^
						70	4.96015 × 10^−4^

### Appendix A.10. Derivation of Equation (61)

Since, from Equation (1),

$$\frac{\partial G\left(t\right)}{\partial \beta }=-G\left(t\right){\left(\frac{t}{\tau_{r}}\right)}^{\beta }\log\left(\frac{t}{\tau_{r}}\right),$$

by (32), the partial derivative

(A21) $$\begin{array}{c} \frac{\partial H_{n}\left(\tau \right)}{\partial \beta }=\frac{1}{\left(n-1\right)!}\left[\sum_{k=1}^{n}\frac{\partial a\left(n,k;\beta \right)}{\partial \beta }{\left(\frac{n\tau }{\tau_{r}}\right)}^{\left(n-k+1\right)\beta }\right]G\left(n\tau \right)+\frac{1}{\left(n-1\right)!}[\sum_{k=1}^{n}a\left(n,k;\beta \right)(n-\\ k+1){\left(\frac{n\tau }{\tau_{r}}\right)}^{\left(n-k+1\right)\beta }]\log\left(\frac{n\tau }{\tau_{r}}\right)G\left(n\tau \right)-H_{n}\left(\tau \right){\left(\frac{n\tau }{\tau_{r}}\right)}^{\beta }\log\left(\frac{n\tau }{\tau_{r}}\right). \end{array}$$

where, according to definitional Equations (25)–(27), the partial derivatives $\frac{\partial a\left(n,k;\beta \right)}{\partial \beta }$ are given by Equations (62)–(64). By (A18) and having in mind (32), Equation (A21) can be written as follows

$$\begin{array}{c} \frac{\partial H_{n}\left(\tau \right)}{\partial \beta }=\frac{1}{\left(n-1\right)!}\left[\sum_{k=1}^{n}\frac{\partial a\left(n,k;\beta \right)}{\partial \beta }{\left(\frac{n\tau }{\tau_{r}}\right)}^{\left(n-k+1\right)\beta }\right]G\left(n\tau \right)+\frac{1}{\left(n-1\right)!}[\sum_{k=1}^{n}a(n,k+\\ 1;\beta ){\left(\frac{n\tau }{\tau_{r}}\right)}^{\left(n-k+1\right)\beta }]\log\left(\frac{n\tau }{\tau_{r}}\right)G\left(n\tau \right)-\frac{1}{\beta }\frac{1}{\left(n-1\right)!}[\sum_{k=1}^{n}a(n+1,k+\\ 1;\beta ){\left(\frac{n\tau }{\tau_{r}}\right)}^{\left(n-k+1\right)\beta }]\log\left(\frac{n\tau }{\tau_{r}}\right)G\left(n\tau \right)-H_{n}\left(\tau \right){\left(\frac{n\tau }{\tau_{r}}\right)}^{\beta }\log\left(\frac{n\tau }{\tau_{r}}\right)+\frac{n}{\beta }H_{n}\left(\tau \right)\log\left(\frac{n\tau }{\tau_{r}}\right). \end{array}$$

whence, after shifting the index k in the second and third sums of the right-hand side according to k→k−1, we obtain

$$\begin{array}{c} \frac{\partial H_{n}\left(\tau \right)}{\partial \beta }=\frac{1}{\left(n-1\right)!}\left[\sum_{k=1}^{n}\frac{\partial a\left(n,k;\beta \right)}{\partial \beta }{\left(\frac{n\tau }{\tau_{r}}\right)}^{\left(n-k+1\right)\beta }\right]G\left(n\tau \right)+\\ \frac{1}{\left(n-1\right)!}\left[\sum_{k=1}^{n}a\left(n,k;\beta \right){\left(\frac{n\tau }{\tau_{r}}\right)}^{\left(n-k+1\right)\beta }\right]{\left(\frac{n\tau }{\tau_{r}}\right)}^{\beta }\log\left(\frac{n\tau }{\tau_{r}}\right)G\left(n\tau \right)-\\ \frac{1}{\left(n-1\right)!}a\left(n,1;\beta \right){\left(\frac{n\tau }{\tau_{r}}\right)}^{\left(n+1\right)\beta }\log\left(\frac{n\tau }{\tau_{r}}\right)G\left(n\tau \right)-\frac{1}{\beta }\frac{1}{\left(n-1\right)!}[\sum_{k=1}^{n+1}a(n+\\ 1,k;\beta ){\left(\frac{n\tau }{\tau_{r}}\right)}^{\left(n+1-k+1\right)\beta }]\log\left(\frac{n\tau }{\tau_{r}}\right)G\left(n\tau \right)+\frac{1}{\beta }\frac{1}{\left(n-1\right)!}a(n+\\ 1,1;\beta ){\left(\frac{n\tau }{\tau_{r}}\right)}^{\left(n+1\right)\beta }\log\left(\frac{n\tau }{\tau_{r}}\right)G\left(n\tau \right)-H_{n}\left(\tau \right){\left(\frac{n\tau }{\tau_{r}}\right)}^{\beta }\log\left(\frac{n\tau }{\tau_{r}}\right)+\frac{n}{\beta }H_{n}\left(\tau \right)\log\left(\frac{n\tau }{\tau_{r}}\right), \end{array}$$

which, including Equations (32), (A6), and the next equation resulting directly from (32) for the n+1 order model by lying $\tau =\frac{n}{n+1}\tau$,

(A22) $$H_{n+1}\left(\frac{n}{n+1}\tau \right)=\frac{1}{\left(n\right)!}\left[\sum_{k=1}^{n+1}a\left(n+1,k;\beta \right){\left(\frac{n\tau }{\tau_{r}}\right)}^{\left(n+1-k+1\right)\beta }\right]G\left(n\tau \right),$$

takes the form

$$\begin{array}{c} \frac{\partial H_{n}\left(\tau \right)}{\partial \beta }=\frac{1}{\left(n-1\right)!}\left[\sum_{k=1}^{n}\frac{\partial a\left(n,k;\beta \right)}{\partial \beta }{\left(\frac{n\tau }{\tau_{r}}\right)}^{\left(n-k+1\right)\beta }\right]G\left(n\tau \right)+H_{n}\left(\tau \right){\left(\frac{n\tau }{\tau_{r}}\right)}^{\beta }\log\left(\frac{n\tau }{\tau_{r}}\right)-\\ \frac{n}{\beta }H_{n+1}\left(\frac{n}{n+1}\tau \right)\log\left(\frac{n\tau }{\tau_{r}}\right)-H_{n}\left(\tau \right){\left(\frac{n\tau }{\tau_{r}}\right)}^{\beta }\log\left(\frac{n\tau }{\tau_{r}}\right)+\frac{n}{\beta }H_{n}\left(\tau \right)\log\left(\frac{n\tau }{\tau_{r}}\right), \end{array}$$

whence the desired equation directly results.

## Appendix B

### Symbols, Mathematical Terminology and Acronyms

$a\left(n,k;\beta \right)$	-real positive coefficients introduced by Equations (25)–(27), –
$G\left(t\right)$	-KWW relaxation modulus, Equation (1), Pa
$G_{0}$	-initial shear modulus, Equation (1), Pa
$H\left(\tau \right)$	-continuous spectrum of relaxation times τ, Equation (3), Pa
$H_{145}\left(\tau \right)$	-the biggest order (*n* = 145) calculated approximation of $H\left(\tau \right)$, Pa
$H_{n}\left(\tau \right)$	-*n*-th order Post–Widder model of the spectrum $H\left(\tau \right)$, Equation (10), Pa
${\bar{H}}_{n}\left(\tau \right)$	-${\bar{H}}_{n}\left(\tau \right)\;\mathrm{perturbed}\;\mathrm{model}\;H_{n}\left(\tau \right)$, Equation (48), Pa
$H_{n}\left(\tau_{n}^{*}\right)$	-$\mathrm{maximum}\;\mathrm{of}\;\mathrm{the}\;\mathrm{model}\;H_{n}\left(\tau \right),\;\mathrm{Equation}\;(35),\;\mathrm{Pa}$
$J\left(n\right)$	-$\mathrm{mean}\;\mathrm{relative}\;\mathrm{index}\;\mathrm{on}\;\mathrm{models}\;H_{n}\left(\tau \right)\;\mathrm{and}\;H_{n+1}\left(\tau \right)$ discrepancy, Equation (40), –
$K_{k}\left(x\right)$	-k order modified Bessel function of the second kind, Equation (7), –
n	-order of the Post–Widder approximation
N	-$\mathrm{number}\;\mathrm{of}\;\mathrm{the}\;\mathrm{relaxation}\;\mathrm{spectrum}\;\mathrm{model}\;H_{n}\left(\tau \right)$ sampling points, –
$\left(\begin{array}{c} n\\ k \end{array}\right)$	-binomial coefficient, Equation (15), –
${\left(p\right)}_{i}$	-failing factorial, Equation (16), –
$Q\left(n\right)$	-mean relative error of the real spectrum approximation, Equation (39), –
$Q_{145}\left(n\right)$	-$\mathrm{mean}\;\mathrm{relative}\;\mathrm{error}\;\mathrm{of}\;\mathrm{the}\;\mathrm{model}\;H_{145}\left(\tau \right)$ approximation, Equation (57), –
T	-$\mathrm{interval}\;T=\left[\tau_{min},\tau_{max}\right]$ of the relaxation times applied in Algorithm
$W_{u,v}\left(x\right)$	-Whittaker function, Equation (8), –
β	-stretching exponent of the KWW modulus, Equation (1), –
$\Gamma \left(x\right)$	-Euler’s gamma function
ε	-small positive number pre-assumed for switching rule, Equation (45), and stopping rule in step 3 of the Algorithm, –
$\tau^{*}$	-$\mathrm{relaxation}\;\mathrm{time}\;\mathrm{maximizing}\;\mathrm{spectrum}\;H\left(\tau \right)$ defined in Equation (54), s
$\tau_{n}^{*}$	-$\mathrm{relaxation}\;\mathrm{time}\;\mathrm{maximizing}\;\mathrm{model}\;H_{n}\left(\tau \right)$ defined in Equation (35), s
$\tau_{r}$	-relaxation time of the KWW modulus, Equation (1), s
$\tau_{j}$	-relaxation times applied for sampling the relaxation spectrum model, s
$ERR\tau^{*}\left(n\right)$	-$\mathrm{relative}\;\mathrm{error}\;\mathrm{of}\;\mathrm{the}\;\mathrm{optimal}\;\mathrm{relaxation}\;\mathrm{time}\;\tau^{*}$ approximation, Equation (55), –
$ERRH^{*}\left(n\right)$	-$\mathrm{square}\;\mathrm{relative}\;\mathrm{error}\;\mathrm{of}\;\mathrm{the}\;\mathrm{maximum}\;H\left(\tau^{*}\right)$ approximation, Equation (56), –
$ERR\tau_{145}^{*}\left(n\right)$	-$\mathrm{relative}\;\mathrm{error}\;\mathrm{of}\;\mathrm{the}\;\mathrm{optimal}\;\mathrm{relaxation}\;\mathrm{time}\;\tau_{145}^{*}$ approximation, Equation (58), –
$ERRH_{145}^{*}\left(n\right)$	-$\mathrm{square}\;\mathrm{relative}\;\mathrm{error}\;\mathrm{of}\;\mathrm{the}\;\mathrm{maximum}\;H_{145}\left(\tau_{145}^{*}\right)$ approximation, Equation (59), –
KWW	-Kohlrausch–Williams–Watts
logt	-natural logarithm of t
$\underset{x\in X}{\max}\;f\left(x\right)$	-$\mathrm{find}\;\mathrm{the}\;\mathrm{value}\;\mathrm{of}\;x\in X,\;\mathrm{which}\;\mathrm{maximizes}\;\mathrm{the}\;\mathrm{function}\;f\left(x\right)$, which maximizes the function *f*(*x*)
