# Supplementary material for: The KWW Relaxation Spectrum Determination Using the Post–Widder Inversion Formula
Source: Materials (Basel). 2026 Aug 1;19(15):3256. doi: 10.3390/ma19153256 (PMC13467595; doi:10.3390/ma19153256)
Supplement: Supplementary file 1 [file materials-19-03256-s001.zip › materials-4289665-supplementary.pdf]

materials

MDPI

Supplementary Materials

The KWW Relaxation Spectrum Determination Using the Post–Widder Inversion Formula

Anna Stankiewicz

Department of Technology Fundamentals, Faculty of Production Engineering, University of Life Sciences in Lublin, 20-612 Lublin, Poland; anna.m.stankiewicz@gmail.com

S1. The Coefficients $\boldsymbol{a(n,k;\beta)}$

To study the properties of the coefficients $a(n,k;\beta)$, defined for two integer arguments $n \ge 1$ and $1 \le k \le n$, let us first consider the coefficients $a(n,2;\beta)$. For $k=2$ and any $n \ge 2$, from (25) and (26), we have

$$a(n+1,2;\beta) = n(1-\beta)\beta^n + \beta a(n,2;\beta). \tag{S1}$$

Equation (S1) generates the sequence of coefficients, which having in mind (27) and (16), starts with $a(2,2;\beta) = -(\beta)_2 = -\beta(\beta-1) = (1-\beta)\beta$. By (S1), the next elements of this sequence are as follows:

$$a(3,2;\beta) = 3(1-\beta)\beta^2,$$

$$a(4,2;\beta) = 6(1-\beta)\beta^3,$$

$$a(5,2;\beta) = 10(1-\beta)\beta^4,$$

$$a(6,2;\beta) = 15(1-\beta)\beta^5.$$

Simple direct formula takes the form

$$a(n,2;\beta) = \frac{n(n-1)}{2}(1-\beta)\beta^{n-1}, \tag{S2}$$

valid for any $n \ge 2$. The proof by mathematical induction is elementary here. For the base case $n=2$, the formula (S2) holds. Assuming that (S2) holds for an arbitrary $n \ge 2$, from (S1) we immediately get

$$a(n+1,2;\beta) = n(1-\beta)\beta^n + \beta\frac{n(n-1)}{2}(1-\beta)\beta^{n-1},$$

which can be expressed as

$$a(n+1,2;\beta) = \frac{n(n-1)+2n}{2}(1-\beta)\beta^n = \frac{n(n+1)}{2}(1-\beta)\beta^n,$$

being formula (S2) for $n+1$.

For $k = 3$, and $n \geq 3$, the recurrence equation (25) is as follows

$$a(n+1,3;\beta) = [n-\beta(n-1)]a(n,2;\beta) + \beta a(n,3;\beta),$$

whence, including (S2), we obtain

$$a(n+1,3;\beta) = \frac{n(n-1)}{2}[n-\beta(n-1)](1-\beta)\beta^{n-1} + \beta a(n,3;\beta),$$

which can be expressed as a sequence

$$a(n+1,3;\beta) = \frac{n^2(n-1)}{2}(1-\beta)\beta^{n-1} - \frac{n(n-1)^2}{2}(1-\beta)\beta^n + \beta a(n,3;\beta), \quad \text{(S3)}$$

starting, for $n = 3$, from the element (compare (27)):

$$a(3,3;\beta) = (\beta)_3 = (1-\beta)\beta(2-\beta). \quad \text{(S4)}$$

Thus, the first elements of the sequence (S3) are as follows

$$a(4,3;\beta) = (1-\beta)\beta^2(11-7\beta), \quad \text{(S5)}$$

$$a(5,3;\beta) = (1-\beta)\beta^3(35-25\beta),$$

$$a(6,3;\beta) = (1-\beta)\beta^4(85-65\beta),$$

which suggest the following general rule

$$a(n+1,3;\beta) = (1-\beta)\beta^{n-1}(x_n - y_n\beta), \quad \text{(S6)}$$

where the sequences $\{x_n\}$ and $\{y_n\}$ result from (S3). Assume that equation (S6) holds for given $n \geq 3$. Then, by (S3), for $n+2$ we have

$$a(n+2,3;\beta) = \frac{(n+1)^2 n}{2}(1-\beta)\beta^n - \frac{(n+1)n^2}{2}(1-\beta)\beta^{n+1}$$
$$+\beta(1-\beta)\beta^{n-1}(x_n - y_n\beta),$$

which can be expressed as

$$a(n+2,3;\beta) = (1-\beta)\beta^n\left[x_n + \frac{(n+1)^2 n}{2} - \left(y_n + \frac{(n+1)n^2}{2}\right)\beta\right],$$

whence we immediately conclude that

$$x_{n+1} = x_n + \frac{(n+1)^2 n}{2}, \quad \text{(S7)}$$

$$y_{n+1} = y_n + \frac{(n+1)n^2}{2}, \quad \text{(S8)}$$

starting, for $n = 3$, with $x_3 = 11$ and $y_3 = 7$; compare (S5).

Thus, for $n \geq 3$, by (S7) we have

$$x_{n+1} = \sum_{m=3}^{n}\frac{(m+1)^2 m}{2} + 11,$$

while, by (S8), the sequence $\{y_n\}$ is generated for $n \geq 3$ according to

$$y_{n+1} = \sum_{m=3}^{n}\frac{(m+1)m^2}{2} + 7.$$

The above series can be rewritten as

https://doi.org/10.3390/ma19153256

$$x_{n+1} = \sum_{m=1}^{n} \frac{(m+1)^2 m}{2} + 11 - 2 - 9 = \sum_{m=1}^{n} \frac{(m+1)^2 m}{2}, \quad \text{(S9)}$$

and

$$y_{n+1} = \sum_{m=1}^{n} \frac{(m+1)m^2}{2} + 7 - 1 - 6 = \sum_{m=1}^{n} \frac{(m+1)m^2}{2} = \sum_{m=1}^{n} \frac{m^3}{2} + \sum_{m=1}^{n} \frac{m^2}{2}. \quad \text{(S10)}$$

To find simpler rules describing the above sequences, the following well-known algebraic formulas will be used:

$$\sum_{m=1}^{n} m(m+1)^2 = \frac{1}{12} n(n+1)(n+2)(3n+5), \quad \text{(S11)}$$

$$\sum_{m=1}^{n} m^2 = \frac{1}{6} n(n+1)(2n+1), \quad \text{(S12)}$$

$$\sum_{m=1}^{n} m^3 = \frac{1}{4} n^2 (n+1)^2. \quad \text{(S13)}$$

By (S11), equation (S9) can be expressed as

$$x_{n+1} = \frac{1}{24} n(n+1)(n+2)(3n+5), \quad \text{(S14)}$$

while, in view of (S12) and (S13), y_{n+1} (S10) is as follows

$$y_{n+1} = \frac{1}{8} n^2 (n+1)^2 + \frac{1}{12} n(n+1)(2n+1) = \frac{1}{24} n(n+1)(3n^2+7n+2). \quad \text{(S15)}$$

Combining equations (S4) and (S6), for $n \geq 2$ we have

$$a(n+1,3;\beta) = (1-\beta)\beta^{n-1}(x_n - y_n\beta),$$

where, having in mind (S14), (S15) and (S4), the factors x_n and y_n for $n \geq 2$ are expressed in compact form as follows:

$$x_n = \frac{1}{24}(n-1)n(n+1)(3n+2),$$

$$y_n = \frac{1}{24}(n-1)n(3n^2+n-2).$$

Such compact formulas can be also derived for the coefficient $a(n,k;\beta)$ for the successive $k \geq 4$. However, definitional recurrence rule (25) seems to be more useful for computations, than direct rules.

S2. Results of Numerical Studies for KWW Spectra Described by Compact Analytical Formulas

S2.1. Stretching exponent $\beta = \frac{1}{3}$

Table S1. For the real relaxation spectrum $\mathcal{H}(\tau)$ (7) of the parameters $G_0 = 0.78$ MPa, $\beta = 1/3$, $\tau_r = 1.08$ s and its Post–Widder n-the order approximate models $\mathcal{H}_n(\tau)$ (32) the quality indices: the mean relative index $J(n)$ (40) of the discrepancy between the models $\mathcal{H}_n(\tau)$ and $\mathcal{H}_{n+1}(\tau)$, the mean relative index $Q(n)$ (39) of the real spectrum $\mathcal{H}(\tau)$ approximation, the relaxation times τ_n^* and the spectra models maxima $\mathcal{H}_n(\tau_n^*)$ defined in equation (35) with the square relative errors $ERR\tau^*(n)$ (55) and $ERRH^*(n)$ (56) of the real spectrum maximum $(\tau^*, \mathcal{H}(\tau^*))$ approximation; $\tau^* = 2.238448$ s, $\mathcal{H}(\tau^*) = 0.106647$ MPa.

n	$J(n)$ [%]	$Q(n)$ [%]	τ_n^* [s]	$\mathcal{H}_n(\tau_n^*)$ [MPa]	$ERR\tau^*(n)$ [%]	$ERRH^*(n)$ [%]
1	2.192060	1.96687×10^{2}	1.080291	0.095649	26.769545	1.063556
2	0.391195	48.092131	1.527649	0.101736	10.083217	0.212097
3	0.148359	20.660065	1.734702	0.103582	5.064421	8.26010×10^{-2}
4	7.27433×10^{-2}	11.323549	1.850569	0.104435	3.002602	4.30030×10^{-2}
5	4.07575×10^{-2}	7.108182	1.923560	0.104921	1.978877	2.61917×10^{-2}

https://doi.org/10.3390/ma19153256

6	2.48716×10^{-2}	4.863923	1.974176	0.105233	1.393823	1.75785×10^{-2}
7	1.61307×10^{-2}	3.532429	2.010509	0.105450	1.036913	1.25981×10^{-2}
8	1.09575×10^{-2}	2.679559	2.038479	0.105609	0.798054	9.46530×10^{-3}
9	7.72152×10^{-3}	2.101175	2.059996	0.105732	0.635552	7.36905×10^{-3}
10	5.60660×10^{-3}	1.691227	2.077143	0.105828	0.519281	5.89837×10^{-3}
11	4.17393×10^{-3}	1.390260	2.092119	0.105906	0.427332	4.82730×10^{-3}
12	3.17395×10^{-3}	1.162872	2.103713	0.105971	0.362299	4.02326×10^{-3}
13	2.45800×10^{-3}	0.986934	2.114202	0.106025	0.308084	3.40443×10^{-3}
14	1.93405×10^{-3}	0.848040	2.122397	0.106071	0.268786	2.91803×10^{-3}
15	1.54321×10^{-3}	0.736488	2.130623	0.106111	0.232031	2.52883×10^{-3}
16	1.24671×10^{-3}	0.645555	2.136518	0.106145	0.207352	2.21256×10^{-3}
17	1.01840×10^{-3}	0.570460	2.14243	0.106176	0.183997	1.95209×10^{-3}
18	8.40224×10^{-4}	0.507731	2.148358	0.106203	0.161979	1.73503×10^{-3}
19	6.99494×10^{-4}	0.454798	2.153112	0.106227	0.145334	1.55225×10^{-3}
20	5.87122×10^{-4}	0.409723	2.156685	0.106248	0.133420	1.39688×10^{-3}
21	4.96499×10^{-4}	0.371027	2.160263	0.106268	0.121997	1.26373×10^{-3}
22	4.22752×10^{-4}	0.337560	2.163848	0.106286	0.111067	1.14874×10^{-3}
23	3.62236×10^{-4}	0.308422	2.167438	0.106302	0.100633	1.04875×10^{-3}
24	3.12196×10^{-4}	0.282898	2.171035	0.106316	9.06982×10^{-2}	9.61268×10^{-4}
25	2.70524×10^{-4}	0.260414	2.173435	0.106330	8.43529×10^{-2}	8.84289×10^{-4}
26	2.35593×10^{-4}	0.240507	2.175839	0.106342	7.82310×10^{-2}	8.16199×10^{-4}
27	2.06134×10^{-4}	0.222796	2.178245	0.106354	7.23334×10^{-2}	7.55680×10^{-4}
28	1.81146×10^{-4}	0.206972	2.180654	0.106365	6.66607×10^{-2}	7.01651×10^{-4}
29	1.59839×10^{-4}	0.192774	2.181859	0.106374	6.39090×10^{-2}	6.53212×10^{-4}
30	1.41580×10^{-4}	0.179988	2.184272	0.106384	5.85752×10^{-2}	6.09622×10^{-4}
31	1.25859×10^{-4}	0.168433	2.185480	0.106392	5.59933×10^{-2}	5.70254×10^{-4}
32	1.12264×10^{-4}	0.157955	2.187897	0.10640	5.10001×10^{-2}	5.34580×10^{-4}
33	1.00458×10^{-4}	0.148425	2.189106	0.106408	4.85889×10^{-2}	5.02149×10^{-4}
$\boldsymbol{n}$	$\boldsymbol{J(n)}$ **[%]**	$\boldsymbol{Q(n)}$ **[%]**	$\boldsymbol{\tau_n^*}$ [s]	$\boldsymbol{\mathcal{H}_n(\tau_n^*)}$ [MPa]	$\boldsymbol{ERR\tau^*(n)}$ **[%]**	$\boldsymbol{ERRH^*(n)}$ **[%]**
34	9.01668×10^{-5}	0.139732	2.190316	0.106415	4.62349×10^{-2}	4.72582×10^{-4}
35	8.11617×10^{-5}	0.131781	2.191527	0.106422	4.39380×10^{-2}	4.45553×10^{-4}
36	7.32545×10^{-5}	0.124489	2.192738	0.106428	4.16985×10^{-2}	4.20777×10^{-4}
37	6.62883×10^{-5}	0.117786	2.19395	0.106434	3.95163×10^{-2}	3.98012×10^{-4}
38	6.01318×10^{-5}	0.111610	2.195163	0.10644	3.73916×10^{-2}	3.77045×10^{-4}
39	5.46744×10^{-5}	0.105907	2.196377	0.106445	3.53245×10^{-2}	3.57692×10^{-4}
40	4.98229×10^{-5}	0.100631	2.197591	0.106450	3.33151×10^{-2}	3.39792×10^{-4}
41	4.54984×10^{-5}	9.57386×10^{-2}	2.198806	0.106455	3.13634×10^{-2}	3.23202×10^{-4}
42	4.16334×10^{-5}	9.11946×10^{-2}	2.200021	0.106460	2.94697×10^{-2}	3.07800×10^{-4}
43	3.81706×10^{-5}	8.69666×10^{-2}	2.200021	0.106464	2.94697×10^{-2}	2.93471×10^{-4}
44	3.50608×10^{-5}	8.30259×10^{-2}	2.201237	0.106469	2.76339×10^{-2}	2.80119×10^{-4}
45	3.22616×10^{-5}	7.93470×10^{-2}	2.202454	0.106473	2.58561×10^{-2}	2.67660×10^{-4}
46	2.97364×10^{-5}	7.59073×10^{-2}	2.202454	0.106476	2.58561×10^{-2}	2.56014×10^{-4}
47	2.74538×10^{-5}	7.26865×10^{-2}	2.203672	0.10648	2.41366×10^{-2}	2.45110×10^{-4}
48	2.53862×10^{-5}	6.96663×10^{-2}	2.20489	0.106484	2.24753×10^{-2}	2.34890×10^{-4}
49	2.35097×10^{-5}	6.68305×10^{-2}	2.20489	0.106487	2.24753×10^{-2}	2.25294×10^{-4}
50	2.18036×10^{-5}	6.41644×10^{-2}	2.206109	0.10649	2.08723×10^{-2}	2.16276×10^{-4}
51	2.02496×10^{-5}	6.16547×10^{-2}	2.206109	0.106493	2.08723×10^{-2}	2.07788×10^{-4}
52	1.88317×10^{-5}	5.92893×10^{-2}	2.207328	0.106496	1.93278×10^{-2}	1.99790×10^{-4}
53	1.75358×10^{-5}	5.70575×10^{-2}	2.207328	0.106499	1.93278×10^{-2}	1.92245×10^{-4}
54	1.63496×10^{-5}	5.49494×10^{-2}	2.208548	0.106502	1.78419×10^{-2}	1.85120×10^{-4}
55	1.52620×10^{-5}	5.29559×10^{-2}	2.208548	0.106505	1.78419×10^{-2}	1.78383×10^{-4}
56	1.42635×10^{-5}	5.10690×10^{-2}	2.209769	0.106507	1.64146×10^{-2}	1.72009×10^{-4}

57	1.33453×10^{-5}	4.92812×10^{-2}	2.209769	0.106510	1.64146×10^{-2}	1.65968×10^{-4}
58	1.24999×10^{-5}	4.75856×10^{-2}	2.209769	0.106512	1.64146×10^{-2}	1.60242×10^{-4}
59	1.17204×10^{-5}	4.59760×10^{-2}	2.210991	0.106514	1.50461×10^{-2}	1.54806×10^{-4}
60	1.10008×10^{-5}	4.44467×10^{-2}	2.210991	0.106517	1.50461×10^{-2}	1.49642×10^{-4}
61	1.03356×10^{-5}	4.29925×10^{-2}	2.212213	0.106519	1.37364×10^{-2}	1.44733×10^{-4}
62	9.71990×10^{-6}	4.16085×10^{-2}	2.212213	0.106521	1.37364×10^{-2}	1.40060×10^{-4}
63	9.14943×10^{-6}	4.02902×10^{-2}	2.212213	0.106523	1.37364×10^{-2}	1.35611×10^{-4}
64	8.62022×10^{-6}	3.90336×10^{-2}	2.213436	0.106525	1.24857×10^{-2}	1.31371×10^{-4}
65	8.12874×10^{-6}	3.78349×10^{-2}	2.213436	0.106527	1.24857×10^{-2}	1.27326×10^{-4}
66	7.67181×10^{-6}	3.66905×10^{-2}	2.213436	0.106529	1.24857×10^{-2}	1.23465×10^{-4}
67	7.24655×10^{-6}	3.55973×10^{-2}	2.214659	0.106530	1.12940×10^{-2}	1.19778×10^{-4}
68	6.85036×10^{-6}	3.45523×10^{-2}	2.214659	0.106532	1.12940×10^{-2}	1.16252×10^{-4}
69	6.48090×10^{-6}	3.35526×10^{-2}	2.214659	0.106534	1.12940×10^{-2}	1.12880×10^{-4}
70	6.13601×10^{-6}	3.25956×10^{-2}	2.214659	0.106535	1.12940×10^{-2}	1.09654×10^{-4}
71	5.81378×10^{-6}	3.16790×10^{-2}	2.215883	0.106537	1.01615×10^{-2}	1.06563×10^{-4}
72	5.51243×10^{-6}	3.08005×10^{-2}	2.215883	0.106538	1.01615×10^{-2}	1.03601×10^{-4}
73	5.23036×10^{-6}	2.99581×10^{-2}	2.215883	0.106540	1.01615×10^{-2}	1.00761×10^{-4}
74	4.96612×10^{-6}	2.91498×10^{-2}	2.215883	0.106541	1.01615×10^{-2}	9.80367×10^{-5}
75	4.71835×10^{-6}	2.83737×10^{-2}	2.217108	0.106543	9.08822×10^{-3}	9.54207×10^{-5}
76	4.48585×10^{-6}	2.76282×10^{-2}	2.217108	0.106544	9.08822×10^{-3}	9.29078×10^{-5}
77	4.26751×10^{-6}	2.69117×10^{-2}	2.217108	0.106546	9.08822×10^{-3}	9.04932×10^{-5}
78	4.06229×10^{-6}	2.62228×10^{-2}	2.217108	0.106547	9.08822×10^{-3}	8.81720×10^{-5}
79	3.86926×10^{-6}	2.55599×10^{-2}	2.218334	0.106548	8.07428×10^{-3}	8.59389×10^{-5}
80	3.68757×10^{-6}	2.49219×10^{-2}	2.218334	0.106549	8.07428×10^{-3}	8.37887×10^{-5}
81	3.51642×10^{-6}	2.43074×10^{-2}	2.218334	0.106551	8.07428×10^{-3}	8.17185×10^{-5}
82	3.35509×10^{-6}	2.37154×10^{-2}	2.218334	0.106552	8.07428×10^{-3}	7.97245×10^{-5}
83	3.20290×10^{-6}	2.31448×10^{-2}	2.218334	0.106553	8.07428×10^{-3}	7.78028×10^{-5}
84	3.05926×10^{-6}	2.25945×10^{-2}	2.219560	0.106554	7.11979×10^{-3}	7.59496×10^{-5}
$\boldsymbol{n}$	$\boldsymbol{J(n)}$ **[%]**	$\boldsymbol{Q(n)}$ **[%]**	$\boldsymbol{\tau_n^*}$ [s]	$\boldsymbol{\mathcal{H}_n(\tau_n^*)}$ [MPa]	$\boldsymbol{ERR\tau^*(n)}$ **[%]**	$\boldsymbol{ERRH^*(n)}$ **[%]**
85	2.92357×10^{-6}	2.20636×10^{-2}	2.219560	0.106555	7.11979×10^{-3}	7.41612×10^{-5}
86	2.79533×10^{-6}	2.15512×10^{-2}	2.219560	0.106556	7.11979×10^{-3}	7.24354×10^{-5}
87	2.67405×10^{-6}	2.10565×10^{-2}	2.219560	0.106557	7.11979×10^{-3}	7.07694×10^{-5}
88	2.55927×10^{-6}	2.05786×10^{-2}	2.219560	0.106558	7.11979×10^{-3}	6.91604×10^{-5}
89	2.45059×10^{-6}	2.01168×10^{-2}	2.219560	0.106559	7.11979×10^{-3}	6.76060×10^{-5}
90	2.34762×10^{-6}	1.96703×10^{-2}	2.220787	0.10656	6.22483×10^{-3}	6.61026×10^{-5}
91	2.25000×10^{-6}	1.92385×10^{-2}	2.220787	0.106561	6.22483×10^{-3}	6.46488×10^{-5}
92	2.15740×10^{-6}	1.88209×10^{-2}	2.220787	0.106562	6.22483×10^{-3}	6.32425×10^{-5}
93	2.06952×10^{-6}	1.84166×10^{-2}	2.220787	0.106563	6.22483×10^{-3}	6.18819×10^{-5}
94	1.98607×10^{-6}	1.80253×10^{-2}	2.220787	0.106564	6.22483×10^{-3}	6.05648×10^{-5}
95	1.90679×10^{-6}	1.76463×10^{-2}	2.220787	0.106565	6.22483×10^{-3}	5.92895×10^{-5}
96	1.83142×10^{-6}	1.72791×10^{-2}	2.222015	0.106566	5.38953×10^{-3}	5.80538×10^{-5}
97	1.75974×10^{-6}	1.69232×10^{-2}	2.222015	0.106567	5.38953×10^{-3}	5.68559×10^{-5}
98	1.69154×10^{-6}	1.65783×10^{-2}	2.222015	0.106567	5.38953×10^{-3}	5.56949×10^{-5}
99	1.62661×10^{-6}	1.62438×10^{-2}	2.222015	0.106568	5.38953×10^{-3}	5.45691×10^{-5}
100	1.56476×10^{-6}	1.59193×10^{-2}	2.222015	0.106569	5.38953×10^{-3}	5.34773×10^{-5}
101	1.50583×10^{-6}	1.56044×10^{-2}	2.222015	0.10657	5.38953×10^{-3}	5.24180×10^{-5}
102	1.44964×10^{-6}	1.52988×10^{-2}	2.222015	0.106571	5.38953×10^{-3}	5.13900×10^{-5}
103	1.39606×10^{-6}	1.50020×10^{-2}	2.223243	0.106571	4.61396×10^{-3}	5.03921×10^{-5}
104	1.34492×10^{-6}	1.47139×10^{-2}	2.223243	0.106572	4.61396×10^{-3}	4.94222×10^{-5}
105	1.29611×10^{-6}	1.44339×10^{-2}	2.223243	0.106573	4.61396×10^{-3}	4.84802×10^{-5}
106	1.24949×10^{-6}	1.41619×10^{-2}	2.223243	0.106573	4.61396×10^{-3}	4.75650×10^{-5}
107	1.20495×10^{-6}	1.38974×10^{-2}	2.223243	0.106574	4.61396×10^{-3}	4.66755×10^{-5}

https://doi.org/10.3390/ma19153256

n	$J(n)$ [%]	$Q(n)$ [%]	τ_n^* [s]	$\mathcal{H}_n(\tau_n^*)$ [MPa]	$ERR\tau^*(n)$ [%]	$ERRH^*(n)$ [%]
108	1.16237×10^{-6}	1.36404×10^{-2}	2.223243	0.106575	4.61396×10^{-3}	4.58108×10^{-5}
109	1.12167×10^{-6}	1.33903×10^{-2}	2.223243	0.106576	4.61396×10^{-3}	4.49700×10^{-5}
110	1.08272×10^{-6}	1.31471×10^{-2}	2.223243	0.106576	4.61396×10^{-3}	4.41522×10^{-5}
111	1.04546×10^{-6}	1.29105×10^{-2}	2.223243	0.106577	4.61396×10^{-3}	4.33566×10^{-5}
112	1.00978×10^{-6}	1.26802×10^{-2}	2.224472	0.106577	3.89823×10^{-3}	4.25821×10^{-5}
113	9.75612×10^{-7}	1.24560×10^{-2}	2.224472	0.106578	3.89823×10^{-3}	4.18279×10^{-5}
114	9.42877×10^{-7}	1.22377×10^{-2}	2.224472	0.106579	3.89823×10^{-3}	4.10936×10^{-5}
115	9.11505×10^{-7}	1.20251×10^{-2}	2.224472	0.106579	3.89823×10^{-3}	4.03785×10^{-5}
116	8.81426×10^{-7}	1.18179×10^{-2}	2.224472	0.10658	3.89823×10^{-3}	3.96820×10^{-5}
117	8.52578×10^{-7}	1.16161×10^{-2}	2.224472	0.106580	3.89823×10^{-3}	3.90034×10^{-5}
118	8.24901×10^{-7}	1.14194×10^{-2}	2.224472	0.106581	3.89823×10^{-3}	3.83421×10^{-5}
119	7.98338×10^{-7}	1.12277×10^{-2}	2.224472	0.106582	3.89823×10^{-3}	3.76976×10^{-5}
120	7.72836×10^{-7}	1.10407×10^{-2}	2.224472	0.106582	3.89823×10^{-3}	3.70692×10^{-5}
121	7.48345×10^{-7}	1.08584×10^{-2}	2.224472	0.106583	3.89823×10^{-3}	3.64564×10^{-5}
122	7.24816×10^{-7}	1.06806×10^{-2}	2.225702	0.106583	3.24245×10^{-3}	3.58587×10^{-5}
123	7.02204×10^{-7}	1.05071×10^{-2}	2.225702	0.106584	3.24245×10^{-3}	3.52752×10^{-5}
124	6.80468×10^{-7}	1.03378×10^{-2}	2.225702	0.106584	3.24245×10^{-3}	3.47059×10^{-5}
125	6.59566×10^{-7}	1.01725×10^{-2}	2.225702	0.106585	3.24245×10^{-3}	3.41503×10^{-5}
126	6.39461×10^{-7}	1.00112×10^{-2}	2.225702	0.106585	3.24245×10^{-3}	3.36079×10^{-5}
127	6.20116×10^{-7}	9.85366×10^{-3}	2.225702	0.106586	3.24245×10^{-3}	3.30784×10^{-5}
128	6.01497×10^{-7}	9.69983×10^{-3}	2.225702	0.106586	3.24245×10^{-3}	3.25614×10^{-5}
129	5.83572×10^{-7}	9.54958×10^{-3}	2.225702	0.106587	3.24245×10^{-3}	3.20564×10^{-5}
130	5.66310×10^{-7}	9.40279×10^{-3}	2.225702	0.106587	3.24245×10^{-3}	3.15631×10^{-5}
131	5.49681×10^{-7}	9.25936×10^{-3}	2.225702	0.106588	3.24245×10^{-3}	3.10812×10^{-5}
132	5.33659×10^{-7}	9.11919×10^{-3}	2.225702	0.106588	3.24245×10^{-3}	3.06102×10^{-5}
133	5.18216×10^{-7}	8.98217×10^{-3}	2.225702	0.106588	3.24245×10^{-3}	3.01499×10^{-5}
134	5.03327×10^{-7}	8.84822×10^{-3}	2.226932	0.106589	2.64671×10^{-3}	2.96999×10^{-5}
135	4.88970×10^{-7}	8.71724×10^{-3}	2.226932	0.106589	2.64671×10^{-3}	2.92596×10^{-5}
136	4.75121×10^{-7}	8.58916×10^{-3}	2.226932	0.106590	2.64671×10^{-3}	2.88290×10^{-5}
137	4.61759×10^{-7}	8.46387×10^{-3}	2.226932	0.106590	2.64671×10^{-3}	2.84079×10^{-5}
138	4.48863×10^{-7}	8.34130×10^{-3}	2.226932	0.106591	2.64671×10^{-3}	2.79960×10^{-5}
139	4.36415×10^{-7}	8.22138×10^{-3}	2.226932	0.106591	2.64671×10^{-3}	2.75929×10^{-5}
140	4.24395×10^{-7}	8.10403×10^{-3}	2.226932	0.106591	2.64671×10^{-3}	2.71986×10^{-5}
141	4.12786×10^{-7}	7.98917×10^{-3}	2.226932	0.106592	2.64671×10^{-3}	2.68127×10^{-5}
142	4.01571×10^{-7}	7.87673×10^{-3}	2.226932	0.106592	2.64671×10^{-3}	2.64349×10^{-5}
143	3.90735×10^{-7}	7.76665×10^{-3}	2.226932	0.106593	2.64671×10^{-3}	2.60651×10^{-5}
144	3.80262×10^{-7}	7.65887×10^{-3}	2.226932	0.106593	2.64671×10^{-3}	2.57030×10^{-5}
145	3.70137×10^{-7}	7.55331×10^{-3}	2.226932	0.106593	2.64671×10^{-3}	2.53484×10^{-5}

S2.2. Stretching Exponent $\beta = \frac{1}{2}$

Table S2. For the real relaxation spectrum $\mathcal{H}(\tau)$ (6) of the parameters $G_0 = 0.78$ MPa, $\beta = 0.5$, $\tau_r = 1.08$ s and its Post–Widder n-the order approximate models $\mathcal{H}_n(\tau)$ (32) the quality indices: the mean relative index $J(n)$ (40) of the discrepancy between the models $\mathcal{H}_n(\tau)$ and $\mathcal{H}_{n+1}(\tau)$, the mean relative index $Q(n)$ (39) of the real spectrum $\mathcal{H}(\tau)$ approximation, the relaxation times τ_n^* and the spectra models maxima $\mathcal{H}_n(\tau_n^*)$ defined in equation (35) with the square relative errors $ERR\tau^*(n)$ (55) and $ERRH^*(n)$ (56) of the real spectrum maximum $(\tau^*, \mathcal{H}(\tau^*))$ approximation; $\tau^* = 2.16$ s, $\mathcal{H}(\tau^*) = 0.188737$ MPa.

n	$J(n)$ [%]	$Q(n)$ [%]	τ_n^* [s]	$\mathcal{H}_n(\tau_n^*)$ [MPa]	$ERR\tau^*(n)$ [%]	$ERRH^*(n)$ [%]
1	7.184564	4.70573×10^{2}	1.079943	0.143473	25.00266	5.751677

https://doi.org/10.3390/ma19153256

2	1.952467	1.57167×10^{2}	1.413719	0.163793	11.93707	1.746774
3	1.089226	76.394025	1.593544	0.171876	6.87741	0.798149
4	0.722908	44.514620	1.705315	0.176118	4.431121	0.447030
5	0.516294	28.930792	1.781743	0.178698	3.066669	0.282950
6	0.385235	20.226049	1.836392	0.180419	2.244565	0.194232
7	0.296443	14.897644	1.877437	0.181645	1.711283	0.141212
8	0.233562	11.410572	1.909526	0.182560	1.344674	0.107134
9	0.187538	9.009255	1.935734	0.183267	1.077999	8.39875×10^{-2}
10	0.152965	7.287935	1.956527	0.183831	0.887373	6.75724×10^{-2}
11	0.126433	6.013411	1.973903	0.184290	0.742282	5.55205×10^{-2}
12	0.105705	5.044157	1.988502	0.184671	0.630394	4.64169×10^{-2}
13	8.92612×10^{-2}	4.290357	2.000995	0.184992	0.541893	3.93756×10^{-2}
14	7.60410×10^{-2}	3.692845	2.012084	0.185266	0.468947	3.38191×10^{-2}
15	6.52874×10^{-2}	3.211390	2.020999	0.185503	0.414122	2.93584×10^{-2}
16	5.64490×10^{-2}	2.817882	2.029954	0.185710	0.362483	2.57238×10^{-2}
17	4.91170×10^{-2}	2.492208	2.036696	0.185892	0.325873	2.27235×10^{-2}
18	4.29836×10^{-2}	2.219679	2.043460	0.186053	0.291099	2.02183×10^{-2}
19	3.78138×10^{-2}	1.989364	2.049492	0.186198	0.261746	1.81051×10^{-2}
20	3.34261×10^{-2}	1.793001	2.054784	0.186327	0.237276	1.63063×10^{-2}
21	2.96787×10^{-2}	1.624247	2.060090	0.186444	0.213947	1.47626×10^{-2}
22	2.64594×10^{-2}	1.478173	2.063889	0.186550	0.197989	1.34279×10^{-2}
23	2.36789×10^{-2}	1.350898	2.068456	0.186647	0.179619	1.22662×10^{-2}
24	2.12655×10^{-2}	1.239338	2.072270	0.186735	0.164964	1.12488×10^{-2}
25	1.91611×10^{-2}	1.141014	2.075326	0.186817	0.153671	1.03529×10^{-2}
26	1.73182×10^{-2}	1.053915	2.078387	0.186892	0.142763	9.55985×10^{-3}
27	1.56979×10^{-2}	0.976400	2.081452	0.186961	0.132241	8.85447×10^{-3}
28	1.42678×10^{-2}	0.907115	2.084522	0.187026	0.122107	8.22433×10^{-3}
29	1.30012×10^{-2}	0.844936	2.086827	0.187085	0.114762	7.65910×10^{-3}
$\boldsymbol{n}$	$\boldsymbol{J(n)}$ **[%]**	$\boldsymbol{Q(n)}$ **[%]**	$\boldsymbol{\tau_n^*}$ [s]	$\boldsymbol{\mathcal{H}_n(\tau_n^*)}$ [MPa]	$\boldsymbol{ERR\tau^*(n)}$ **[%]**	$\boldsymbol{ERRH^*(n)}$ **[%]**
30	1.18756×10^{-2}	0.788927	2.089134	0.187141	0.107637	7.15018×10^{-3}
31	1.08723×10^{-2}	0.738297	2.091445	0.187193	0.100733	6.69031×10^{-3}
32	9.97527×10^{-3}	0.692381	2.093758	0.187242	9.40510×10^{-2}	6.27341×10^{-3}
33	9.17103×10^{-3}	0.650612	2.095301	0.187288	8.97196×10^{-2}	5.89428×10^{-3}
34	8.44807×10^{-3}	0.612506	2.097618	0.187331	8.34082×10^{-2}	5.54849×10^{-3}
35	7.79654×10^{-3}	0.577647	2.099164	0.187372	7.93248×10^{-2}	5.23224×10^{-3}
36	7.20797×10^{-3}	0.545678	2.100712	0.187410	7.53409×10^{-2}	4.94228×10^{-3}
37	6.67507×10^{-3}	0.516287	2.102260	0.187447	7.14569×10^{-2}	4.67576×10^{-3}
38	6.19152×10^{-3}	0.489205	2.103810	0.187481	6.76729×10^{-2}	4.43023×10^{-3}
39	5.75184×10^{-3}	0.464197	2.105360	0.187513	6.39891×10^{-2}	4.20353×10^{-3}
40	5.35125×10^{-3}	0.441057	2.106912	0.187544	6.04059×10^{-2}	3.99379×10^{-3}
41	4.98560×10^{-3}	0.419602	2.107689	0.187574	5.86520×10^{-2}	3.79937×10^{-3}
42	4.65123×10^{-3}	0.399675	2.109242	0.187602	5.52199×10^{-2}	3.61879×10^{-3}
43	4.34492×10^{-3}	0.381132	2.110020	0.187628	5.35417×10^{-2}	3.45080×10^{-3}
44	4.06386×10^{-3}	0.363849	2.111575	0.187654	5.02613×10^{-2}	3.29422×10^{-3}
45	3.80555×10^{-3}	0.347714	2.112353	0.187678	4.86591×10^{-2}	3.14807×10^{-3}
46	3.56779×10^{-3}	0.332629	2.113910	0.187701	4.55308×10^{-2}	3.01144×10^{-3}
47	3.34861×10^{-3}	0.318503	2.114689	0.187724	4.40048×10^{-2}	2.88350×10^{-3}
48	3.14627×10^{-3}	0.305258	2.115468	0.187745	4.25042×10^{-2}	2.76354×10^{-3}
49	2.95922×10^{-3}	0.292821	2.116248	0.187765	4.10292×10^{-2}	2.65092×10^{-3}
50	2.78609×10^{-3}	0.281128	2.117028	0.187785	3.95797×10^{-2}	2.54504×10^{-3}
51	2.62561×10^{-3}	0.270122	2.117808	0.187804	3.81557×10^{-2}	2.44538×10^{-3}
52	2.47670×10^{-3}	0.259749	2.118588	0.187822	3.67573×10^{-2}	2.35146×10^{-3}

53	2.33835×10^{-3}	0.249962	2.119369	0.187839	3.53845×10^{-2}	2.26285×10^{-3}
54	2.20965×10^{-3}	0.240717	2.120150	0.187856	3.40373×10^{-2}	2.17915×10^{-3}
55	2.08981×10^{-3}	0.231976	2.120931	0.187872	3.27158×10^{-2}	2.10001×10^{-3}
56	1.97810×10^{-3}	0.223702	2.121712	0.187888	3.14200×10^{-2}	2.02511×10^{-3}
57	1.87385×10^{-3}	0.215862	2.122494	0.187903	3.01499×10^{-2}	1.95414×10^{-3}
58	1.77646×10^{-3}	0.208427	2.123276	0.187917	2.89056×10^{-2}	1.88684×10^{-3}
59	1.68540×10^{-3}	0.201370	2.124059	0.187931	2.76870×10^{-2}	1.82296×10^{-3}
60	1.60018×10^{-3}	0.194665	2.124059	0.187945	2.76870×10^{-2}	1.76226×10^{-3}
61	1.52033×10^{-3}	0.188289	2.124842	0.187958	2.64943×10^{-2}	1.70454×10^{-3}
62	1.44547×10^{-3}	0.182221	2.125625	0.187971	2.53274×10^{-2}	1.64962×10^{-3}
63	1.37521×10^{-3}	0.176441	2.125625	0.187983	2.53274×10^{-2}	1.59731×10^{-3}
64	1.30921×10^{-3}	0.170933	2.126408	0.187995	2.41863×10^{-2}	1.54744×10^{-3}
65	1.24718×10^{-3}	0.165678	2.127191	0.188006	2.30712×10^{-2}	1.49988×10^{-3}
66	1.18881×10^{-3}	0.160661	2.127191	0.188017	2.30712×10^{-2}	1.45448×10^{-3}
67	1.13385×10^{-3}	0.155869	2.127975	0.188028	2.19819×10^{-2}	1.41110×10^{-3}
68	1.08207×10^{-3}	0.151288	2.128759	0.188039	2.09186×10^{-2}	1.36964×10^{-3}
69	1.03324×10^{-3}	0.146906	2.128759	0.188049	2.09186×10^{-2}	1.32998×10^{-3}
70	9.87167×10^{-4}	0.142712	2.129544	0.188059	1.98813×10^{-2}	1.29201×10^{-3}
71	9.43656×10^{-4}	0.138694	2.129544	0.188068	1.98813×10^{-2}	1.25565×10^{-3}
72	9.02540×10^{-4}	0.134844	2.130328	0.188078	1.88700×10^{-2}	1.22080×10^{-3}
73	8.63660×10^{-4}	0.131152	2.130328	0.188087	1.88700×10^{-2}	1.18739×10^{-3}
74	8.26872×10^{-4}	0.127609	2.131113	0.188096	1.78848×10^{-2}	1.15532×10^{-3}
75	7.92040×10^{-4}	0.124208	2.131113	0.188104	1.78848×10^{-2}	1.12454×10^{-3}
76	7.59039×10^{-4}	0.120941	2.131899	0.188113	1.69256×10^{-2}	1.09497×10^{-3}
77	7.27756×10^{-4}	0.117801	2.131899	0.188121	1.69256×10^{-2}	1.06656×10^{-3}
78	6.98082×10^{-4}	0.114782	2.132684	0.188129	1.59925×10^{-2}	1.03923×10^{-3}
79	6.69918×10^{-4}	0.111878	2.132684	0.188136	1.59925×10^{-2}	1.01294×10^{-3}
80	6.43172×10^{-4}	0.109082	2.133470	0.188144	1.50855×10^{-2}	9.87638×10^{-4}
$\boldsymbol{n}$	$\boldsymbol{J(n)}$ **[%]**	$\boldsymbol{Q(n)}$ **[%]**	$\boldsymbol{\tau_n^*}$ [s]	$\boldsymbol{\mathcal{H}_n(\tau_n^*)}$ [MPa]	$\boldsymbol{ERR\tau^*(n)}$ **[%]**	$\boldsymbol{ERRH^*(n)}$ **[%]**
81	6.17760×10^{-4}	0.106390	2.133470	0.188151	1.50855×10^{-2}	9.63268×10^{-4}
82	5.93600×10^{-4}	0.103796	2.133470	0.188159	1.50855×10^{-2}	9.39795×10^{-4}
83	5.70620×10^{-4}	0.101296	2.134256	0.188166	1.42047×10^{-2}	9.17163×10^{-4}
84	5.48750×10^{-4}	9.88847×10^{-2}	2.134256	0.188172	1.42047×10^{-2}	8.95342×10^{-4}
85	5.27926×10^{-4}	9.65588×10^{-2}	2.135043	0.188179	1.33501×10^{-2}	8.74292×10^{-4}
86	5.08089×10^{-4}	9.43140×10^{-2}	2.135043	0.188186	1.33501×10^{-2}	8.53971×10^{-4}
87	4.89182×10^{-4}	9.21466×10^{-2}	2.135043	0.188192	1.33501×10^{-2}	8.34356×10^{-4}
88	4.71152×10^{-4}	9.00530×10^{-2}	2.135830	0.188198	1.25217×10^{-2}	8.15405×10^{-4}
89	4.53952×10^{-4}	8.80299×10^{-2}	2.135830	0.188204	1.25217×10^{-2}	7.97093×10^{-4}
90	4.37536×10^{-4}	8.60742×10^{-2}	2.135830	0.188210	1.25217×10^{-2}	7.79394×10^{-4}
91	4.21860×10^{-4}	8.41829×10^{-2}	2.136617	0.188216	1.17195×10^{-2}	7.62274×10^{-4}
92	4.06885×10^{-4}	8.23533×10^{-2}	2.136617	0.188222	1.17195×10^{-2}	7.45712×10^{-4}
93	3.92574×10^{-4}	8.05827×10^{-2}	2.136617	0.188227	1.17195×10^{-2}	7.29688×10^{-4}
94	3.78890×10^{-4}	7.88686×10^{-2}	2.137404	0.188233	1.09436×10^{-2}	7.14172×10^{-4}
95	3.65802×10^{-4}	7.72085×10^{-2}	2.137404	0.188238	1.09436×10^{-2}	6.99145×10^{-4}
96	3.53277×10^{-4}	7.56004×10^{-2}	2.137404	0.188243	1.09436×10^{-2}	6.84589×10^{-4}
97	3.41288×10^{-4}	7.40419×10^{-2}	2.138192	0.188248	1.01940×10^{-2}	6.70485×10^{-4}
98	3.29807×10^{-4}	7.25311×10^{-2}	2.138192	0.188253	1.01940×10^{-2}	6.56806×10^{-4}
99	3.18807×10^{-4}	7.10661×10^{-2}	2.138192	0.188258	1.01940×10^{-2}	6.43545×10^{-4}
100	3.08265×10^{-4}	6.96450×10^{-2}	2.138192	0.188263	1.01940×10^{-2}	6.30684×10^{-4}
101	2.98158×10^{-4}	6.82661×10^{-2}	2.138979	0.188268	9.47070×10^{-3}	6.18202×10^{-4}
102	2.88464×10^{-4}	6.69277×10^{-2}	2.138979	0.188273	9.47070×10^{-3}	6.06085×10^{-4}
103	2.79163×10^{-4}	6.56284×10^{-2}	2.138979	0.188277	9.47070×10^{-3}	5.94324×10^{-4}

104	2.70237×10^{-4}	6.43664×10^{-2}	2.138979	0.188281	9.47070×10^{-3}	5.82904×10^{-4}
105	2.61667×10^{-4}	6.31406×10^{-2}	2.139768	0.188286	8.77379×10^{-3}	5.71805×10^{-4}
106	2.53435×10^{-4}	6.19494×10^{-2}	2.139768	0.188290	8.77379×10^{-3}	5.61021×10^{-4}
107	2.45527×10^{-4}	6.07916×10^{-2}	2.139768	0.188294	8.77379×10^{-3}	5.50540×10^{-4}
108	2.37927×10^{-4}	5.96659×10^{-2}	2.139768	0.188298	8.77379×10^{-3}	5.40353×10^{-4}
109	2.30620×10^{-4}	5.85712×10^{-2}	2.140556	0.188302	8.10327×10^{-3}	5.30442×10^{-4}
110	2.23593×10^{-4}	5.75064×10^{-2}	2.140556	0.188306	8.10327×10^{-3}	5.20801×10^{-4}
111	2.16833×10^{-4}	5.64703×10^{-2}	2.140556	0.188310	8.10327×10^{-3}	5.11422×10^{-4}
112	2.10328×10^{-4}	5.54620×10^{-2}	2.140556	0.188314	8.10327×10^{-3}	5.02295×10^{-4}
113	2.04066×10^{-4}	5.44804×10^{-2}	2.141345	0.188318	7.45916×10^{-3}	4.93412×10^{-4}
114	1.98037×10^{-4}	5.35247×10^{-2}	2.141345	0.188322	7.45916×10^{-3}	4.84757×10^{-4}
115	1.92230×10^{-4}	5.25939×10^{-2}	2.141345	0.188325	7.45916×10^{-3}	4.76330×10^{-4}
116	1.86636×10^{-4}	5.16871×10^{-2}	2.141345	0.188329	7.45916×10^{-3}	4.68121×10^{-4}
117	1.81244×10^{-4}	5.08036×10^{-2}	2.141345	0.188332	7.45916×10^{-3}	4.60125×10^{-4}
118	1.76047×10^{-4}	4.99426×10^{-2}	2.142134	0.188336	6.84150×10^{-3}	4.52331×10^{-4}
119	1.71036×10^{-4}	4.91033×10^{-2}	2.142134	0.188339	6.84150×10^{-3}	4.44730×10^{-4}
120	1.66203×10^{-4}	4.82849×10^{-2}	2.142134	0.188342	6.84150×10^{-3}	4.37321×10^{-4}
121	1.61540×10^{-4}	4.74868×10^{-2}	2.142134	0.188346	6.84150×10^{-3}	4.30096×10^{-4}
122	1.57040×10^{-4}	4.67084×10^{-2}	2.142134	0.188349	6.84150×10^{-3}	4.23049×10^{-4}
123	1.52696×10^{-4}	4.59489×10^{-2}	2.142134	0.188352	6.84150×10^{-3}	4.16175×10^{-4}
124	1.48503×10^{-4}	4.52078×10^{-2}	2.142923	0.188355	6.25032×10^{-3}	4.09464×10^{-4}
125	1.44453×10^{-4}	4.44845×10^{-2}	2.142923	0.188358	6.25032×10^{-3}	4.02914×10^{-4}
126	1.40540×10^{-4}	4.37784×10^{-2}	2.142923	0.188361	6.25032×10^{-3}	3.96521×10^{-4}
127	1.36760×10^{-4}	4.30890×10^{-2}	2.142923	0.188364	6.25032×10^{-3}	3.90280×10^{-4}
128	1.33107×10^{-4}	4.24157×10^{-2}	2.142923	0.188367	6.25032×10^{-3}	3.84186×10^{-4}
129	1.29576×10^{-4}	4.17581×10^{-2}	2.143713	0.188370	5.68564×10^{-3}	3.78233×10^{-4}
130	1.26161×10^{-4}	4.11157×10^{-2}	2.143713	0.188373	5.68564×10^{-3}	3.72415×10^{-4}
131	1.22858×10^{-4}	4.04879×10^{-2}	2.143713	0.188376	5.68564×10^{-3}	3.66731×10^{-4}
$\boldsymbol{n}$	$\boldsymbol{J(n)}$ **[%]**	$\boldsymbol{Q(n)}$ **[%]**	$\boldsymbol{\tau_n^*}$ [s]	$\boldsymbol{\mathcal{H}_n(\tau_n^*)}$ [MPa]	$\boldsymbol{ERR\tau^*(n)}$ **[%]**	$\boldsymbol{ERRH^*(n)}$ **[%]**
132	1.19664×10^{-4}	3.98745×10^{-2}	2.143713	0.188378	5.68564×10^{-3}	3.61176×10^{-4}
133	1.16573×10^{-4}	3.92748×10^{-2}	2.143713	0.188381	5.68564×10^{-3}	3.55747×10^{-4}
134	1.13581×10^{-4}	3.86886×10^{-2}	2.143713	0.188384	5.68564×10^{-3}	3.50441×10^{-4}
135	1.10686×10^{-4}	3.81155×10^{-2}	2.143713	0.188386	5.68564×10^{-3}	3.45252×10^{-4}
136	1.07882×10^{-4}	3.75549×10^{-2}	2.144503	0.188389	5.14750×10^{-3}	3.40177×10^{-4}
137	1.05167×10^{-4}	3.70067×10^{-2}	2.144503	0.188392	5.14750×10^{-3}	3.35212×10^{-4}
138	1.02537×10^{-4}	3.64703×10^{-2}	2.144503	0.188394	5.14750×10^{-3}	3.30355×10^{-4}
139	9.99891×10^{-5}	3.59456×10^{-2}	2.144503	0.188397	5.14750×10^{-3}	3.25603×10^{-4}
140	9.75202×10^{-5}	3.54320×10^{-2}	2.144503	0.188399	5.14750×10^{-3}	3.20954×10^{-4}
141	9.51273×10^{-5}	3.49294×10^{-2}	2.144503	0.188401	5.14750×10^{-3}	3.16403×10^{-4}
142	9.28077×10^{-5}	3.44374×10^{-2}	2.144503	0.188404	5.14750×10^{-3}	3.11950×10^{-4}
143	9.05586×10^{-5}	3.39558×10^{-2}	2.145293	0.188406	4.63593×10^{-3}	3.07589×10^{-4}
144	8.83775×10^{-5}	3.34841×10^{-2}	2.145293	0.188408	4.63593×10^{-3}	3.03317×10^{-4}
145	8.62619×10^{-5}	3.30223×10^{-2}	2.145293	0.188411	4.63593×10^{-3}	2.99134×10^{-4}

S2.3. Stretching Exponent $\beta = \frac{2}{3}$

Table S3. For the real relaxation spectrum $\mathcal{H}(\tau)$ (8) of the parameters $G_0 = 0.78$ MPa, $\beta = 2/3$, $\tau_r = 1.08$ s and its Post–Widder n-the order approximate models $\mathcal{H}_n(\tau)$ (32) the quality indices: the mean relative index $J(n)$ (40) of the discrepancy between the models $\mathcal{H}_n(\tau)$ and $\mathcal{H}_{n+1}(\tau)$, the mean relative index $Q(n)$ (39) of the real spectrum $\mathcal{H}(\tau)$ approximation, the relaxation times τ_n^* and the spectra models maxima $\mathcal{H}_n(\tau_n^*)$ defined in equation (35) with the square relative errors

$ERR\tau^*(n)$ (55) and $ERRH^*(n)$ (56) of the real spectrum maximum $\left(\tau^*, \mathcal{H}(\tau^*)\right)$ approximation; $\tau^* = 1.931459$ s, $\mathcal{H}(\tau^*) = 0.338774$ MPa.

n	$J(n)$ [%]	$Q(n)$ [%]	τ_n^* [s]	$\mathcal{H}_n(\tau_n^*)$ [MPa]	$ERR\tau^*(n)$ [%]	$ERRH^*(n)$ [%]
1	11.86987	50.496119	1.079893	0.191297	19.43866	18.950792
2	1.559557	19.172710	1.283164	0.237259	11.26614	8.97927
3	0.483316	11.925662	1.399532	0.260881	7.58462	5.286679
4	0.237808	8.583919	1.478371	0.275582	5.502954	3.479431
5	0.147610	6.595342	1.536327	0.28567	4.185185	2.45716
6	0.103551	5.269375	1.580826	0.293031	3.295622	1.823201
7	7.79033×10^{-2}	4.325854	1.616159	0.298637	2.664884	1.403721
8	6.12226×10^{-2}	3.624646	1.645449	0.303045	2.192781	1.112315
9	4.95562×10^{-2}	3.086620	1.669492	0.306599	1.839601	0.902013
10	4.09798×10^{-2}	2.663420	1.689992	0.309524	1.562964	0.745495
11	3.44450×10^{-2}	2.323786	1.707594	0.31197	1.343397	0.626003
12	2.93303×10^{-2}	2.046634	1.722998	0.314046	1.164885	0.532802
13	2.52422×10^{-2}	1.817247	1.73614	0.315828	1.022636	0.458764
14	2.19192×10^{-2}	1.625069	1.747772	0.317375	0.904459	0.399012
15	1.91804×10^{-2}	1.462347	1.758267	0.318729	0.804059	0.350119
16	1.68964×10^{-2}	1.323271	1.767604	0.319924	0.719704	0.309622
17	1.49725×10^{-2}	1.203416	1.776172	0.320986	0.646405	0.275714
18	1.33376×10^{-2}	1.099354	1.783549	0.321936	0.586449	0.247049
19	1.19375×10^{-2}	1.008396	1.790544	0.322791	0.532289	0.222604
20	1.07303×10^{-2}	0.928409	1.796739	0.323563	0.486515	0.201595
21	9.68290×10^{-3}	0.857680	1.802541	0.324266	0.445516	0.183409
22	8.76906×10^{-3}	0.794820	1.807945	0.324907	0.40895	0.167566
23	7.96769×10^{-3}	0.738693	1.812947	0.325494	0.376495	0.15368
24	7.26165×10^{-3}	0.688364	1.817127	0.326033	0.350409	0.141444
25	6.63694×10^{-3}	0.643054	1.821316	0.326531	0.325202	0.130607
n	$J(n)$ [%]	$Q(n)$ [%]	τ_n^* [s]	$\mathcal{H}_n(\tau_n^*)$ [MPa]	$ERR\tau^*(n)$ [%]	$ERRH^*(n)$ [%]
26	6.08199×10^{-3}	0.602112	1.825514	0.326992	0.300882	0.120965
27	5.58719×10^{-3}	0.564990	1.82888	0.327419	0.282067	0.112349
28	5.14451×10^{-3}	0.531225	1.832252	0.327817	0.263827	0.104618
29	4.74719×10^{-3}	0.500420	1.835631	0.328188	0.246165	9.76560×10^{-2}
30	4.38952×10^{-3}	0.472238	1.838592	0.328534	0.231187	9.13645×10^{-2}
31	4.06662×10^{-3}	0.446387	1.841134	0.328859	0.218705	8.56604×10^{-2}
32	3.77435×10^{-3}	0.422615	1.844104	0.329164	0.204559	8.04727×10^{-2}
33	3.50914×10^{-3}	0.400705	1.846228	0.329451	0.194731	7.57413×10^{-2}
34	3.26792×10^{-3}	0.380465	1.84878	0.329721	0.183242	7.14141×10^{-2}
35	3.04802×10^{-3}	0.361730	1.85091	0.329976	0.173923	6.74466×10^{-2}
36	2.84714×10^{-3}	0.344352	1.853042	0.330217	0.164837	6.38001×10^{-2}
37	2.66326×10^{-3}	0.328204	1.855177	0.330446	0.155985	6.04409×10^{-2}
38	2.49462×10^{-3}	0.313172	1.856887	0.330662	0.149072	5.73397×10^{-2}
39	2.33968×10^{-3}	0.299154	1.859026	0.330868	0.140643	5.44708×10^{-2}
40	2.19707×10^{-3}	0.286060	1.860739	0.331063	0.134069	5.18115×10^{-2}
41	2.06559×10^{-3}	0.273812	1.862025	0.331249	0.129238	4.93420×10^{-2}
42	1.94418×10^{-3}	0.262338	1.86374	0.331426	0.12293	4.70446×10^{-2}
43	1.83190×10^{-3}	0.251573	1.865458	0.331595	0.116774	4.49038×10^{-2}
44	1.72792×10^{-3}	0.241460	1.866747	0.331757	0.112257	4.29056×10^{-2}
45	1.63147×10^{-3}	0.231947	1.868037	0.331911	0.107826	4.10377×10^{-2}
46	1.54191×10^{-3}	0.222987	1.869328	0.332059	0.103482	3.92891×10^{-2}
47	1.45862×10^{-3}	0.214539	1.870619	0.332201	9.92236×10^{-2}	3.76497×10^{-2}
48	1.38107×10^{-3}	0.206563	1.871912	0.332337	9.50521×10^{-2}	3.61107×10^{-2}

49	1.30878×10^{-3}	0.199026	1.873206	0.332467	9.09674×10^{-2}	3.46640×10^{-2}
50	1.24131×10^{-3}	0.191895	1.874068	0.332592	8.82925×10^{-2}	3.33025×10^{-2}
51	1.17828×10^{-3}	0.185142	1.875364	0.332712	8.43529×10^{-2}	3.20195×10^{-2}
52	1.11932×10^{-3}	0.178740	1.876227	0.332828	8.17749×10^{-2}	3.08093×10^{-2}
53	1.06412×10^{-3}	0.172666	1.877524	0.332939	7.79808×10^{-2}	2.96663×10^{-2}
54	1.01239×10^{-3}	0.166897	1.878389	0.333046	7.55001×10^{-2}	2.85856×10^{-2}
55	9.63850×10^{-4}	0.161413	1.879254	0.33315	7.30583×10^{-2}	2.75629×10^{-2}
56	9.18275×10^{-4}	0.156196	1.880120	0.33325	7.06556×10^{-2}	2.65941×10^{-2}
57	8.75439×10^{-4}	0.151229	1.880986	0.333346	6.82920×10^{-2}	2.56754×10^{-2}
58	8.35143×10^{-4}	0.146495	1.881852	0.333439	6.59675×10^{-2}	2.48035×10^{-2}
59	7.97204×10^{-4}	0.141981	1.882719	0.333529	6.36822×10^{-2}	2.39752×10^{-2}
60	7.61454×10^{-4}	0.137673	1.883586	0.333616	6.14362×10^{-2}	2.31877×10^{-2}
61	7.27739×10^{-4}	0.133558	1.884020	0.333700	6.03279×10^{-2}	2.24383×10^{-2}
62	6.95919×10^{-4}	0.129626	1.884888	0.333781	5.81409×10^{-2}	2.17246×10^{-2}
63	6.65865×10^{-4}	0.125865	1.885756	0.333860	5.59933×10^{-2}	2.10445×10^{-2}
64	6.37457×10^{-4}	0.122266	1.886190	0.333936	5.49343×10^{-2}	2.03958×10^{-2}
65	6.10587×10^{-4}	0.118819	1.887059	0.334010	5.28458×10^{-2}	1.97765×10^{-2}
66	5.85152×10^{-4}	0.115517	1.887494	0.334082	5.18165×10^{-2}	1.91851×10^{-2}
67	5.61061×10^{-4}	0.112350	1.888363	0.334152	4.97874×10^{-2}	1.86197×10^{-2}
68	5.38226×10^{-4}	0.109312	1.888798	0.334219	4.87877×10^{-2}	1.80790×10^{-2}
69	5.16569×10^{-4}	0.106396	1.889668	0.334285	4.68180×10^{-2}	1.75615×10^{-2}
70	4.96015×10^{-4}	0.103596	1.890103	0.334349	4.58481×10^{-2}	1.70658×10^{-2}
71	4.76498×10^{-4}	0.100904	1.890539	0.334411	4.48881×10^{-2}	1.65909×10^{-2}
72	4.57952×10^{-4}	9.83169×10^{-2}	1.891409	0.334471	4.29979×10^{-2}	1.61355×10^{-2}
73	4.40320×10^{-4}	9.58278×10^{-2}	1.891845	0.33453	4.20677×10^{-2}	1.56986×10^{-2}
74	4.23547×10^{-4}	9.34323×10^{-2}	1.892281	0.334587	4.11475×10^{-2}	1.52791×10^{-2}
75	4.07582×10^{-4}	9.11256×10^{-2}	1.892716	0.334642	4.02373×10^{-2}	1.48763×10^{-2}
76	3.92379×10^{-4}	8.89034×10^{-2}	1.893152	0.334696	3.93370×10^{-2}	1.44892×10^{-2}

n	$J(n)$ [%]	$Q(n)$ [%]	τ_n^* [s]	$\mathcal{H}_n(\tau_n^*)$ [MPa]	$ERR\tau^*(n)$ [%]	$ERRH^*(n)$ [%]
77	3.77892×10^{-4}	8.67617×10^{-2}	1.894024	0.334749	3.75665×10^{-2}	1.41170×10^{-2}
78	3.64082×10^{-4}	8.46965×10^{-2}	1.89446	0.3348	3.66962×10^{-2}	1.37589×10^{-2}
79	3.50910×10^{-4}	8.27044×10^{-2}	1.894897	0.334851	3.58359×10^{-2}	1.34143×10^{-2}
80	3.38340×10^{-4}	8.07818×10^{-2}	1.895333	0.334899	3.49856×10^{-2}	1.30825×10^{-2}
81	3.26339×10^{-4}	7.89256×10^{-2}	1.89577	0.334947	3.41453×10^{-2}	1.27628×10^{-2}
82	3.14876×10^{-4}	7.71327×10^{-2}	1.896206	0.334993	3.33151×10^{-2}	1.24547×10^{-2}
83	3.03921×10^{-4}	7.54004×10^{-2}	1.896643	0.335039	3.24949×10^{-2}	1.21576×10^{-2}
84	2.93448×10^{-4}	7.37258×10^{-2}	1.89708	0.335083	3.16847×10^{-2}	1.18710×10^{-2}
85	2.83430×10^{-4}	7.21065×10^{-2}	1.89708	0.335126	3.16847×10^{-2}	1.15944×10^{-2}
86	2.73844×10^{-4}	7.05400×10^{-2}	1.897517	0.335169	3.08846×10^{-2}	1.13274×10^{-2}
87	2.64668×10^{-4}	6.90241×10^{-2}	1.897953	0.33521	3.00945×10^{-2}	1.10695×10^{-2}
88	2.55879×10^{-4}	6.75566×10^{-2}	1.898391	0.33525	2.93145×10^{-2}	1.08203×10^{-2}
89	2.47459×10^{-4}	6.61355×10^{-2}	1.898828	0.33529	2.85445×10^{-2}	1.05794×10^{-2}
90	2.39388×10^{-4}	6.47588×10^{-2}	1.899265	0.335328	2.77846×10^{-2}	1.03464×10^{-2}
91	2.31650×10^{-4}	6.34247×10^{-2}	1.899702	0.335366	2.70348×10^{-2}	1.01211×10^{-2}
92	2.24227×10^{-4}	6.21315×10^{-2}	1.899702	0.335403	2.70348×10^{-2}	9.90308×10^{-3}
93	2.17103×10^{-4}	6.08774×10^{-2}	1.90014	0.335439	2.62951×10^{-2}	9.69199×10^{-3}
94	2.10266×10^{-4}	5.96610×10^{-2}	1.900577	0.335474	2.55655×10^{-2}	9.48758×10^{-3}
95	2.03699×10^{-4}	5.84808×10^{-2}	1.901015	0.335509	2.48460×10^{-2}	9.28959×10^{-3}
96	1.97391×10^{-4}	5.73352×10^{-2}	1.901015	0.335543	2.48460×10^{-2}	9.09771×10^{-3}
97	1.91330×10^{-4}	5.62230×10^{-2}	1.901453	0.335576	2.41366×10^{-2}	8.91170×10^{-3}
98	1.85503×10^{-4}	5.51430×10^{-2}	1.901891	0.335609	2.34373×10^{-2}	8.73136×10^{-3}
99	1.79899×10^{-4}	5.40937×10^{-2}	1.901891	0.335641	2.34373×10^{-2}	8.55644×10^{-3}

100	1.74509×10^{-4}	5.30742×10^{-2}	1.902329	0.335672	2.27481×10^{-2}	8.38670×10^{-3}
101	1.69322×10^{-4}	5.20832×10^{-2}	1.902767	0.335702	2.20690×10^{-2}	8.22198×10^{-3}
102	1.64330×10^{-4}	5.11198×10^{-2}	1.902767	0.335732	2.20690×10^{-2}	8.06205×10^{-3}
103	1.59523×10^{-4}	5.01829×10^{-2}	1.903205	0.335762	2.14001×10^{-2}	7.90674×10^{-3}
104	1.54892×10^{-4}	4.92716×10^{-2}	1.903643	0.335791	2.07414×10^{-2}	7.75590×10^{-3}
105	1.50432×10^{-4}	4.83848×10^{-2}	1.903643	0.335819	2.07414×10^{-2}	7.60930×10^{-3}
106	1.46132×10^{-4}	4.75218×10^{-2}	1.904082	0.335847	2.00927×10^{-2}	7.46683×10^{-3}
107	1.41988×10^{-4}	4.66818×10^{-2}	1.904082	0.335874	2.00927×10^{-2}	7.32833×10^{-3}
108	1.37991×10^{-4}	4.58638×10^{-2}	1.90452	0.335901	1.94543×10^{-2}	7.19363×10^{-3}
109	1.34137×10^{-4}	4.50671×10^{-2}	1.90452	0.335927	1.94543×10^{-2}	7.06264×10^{-3}
110	1.30417×10^{-4}	4.42911×10^{-2}	1.904959	0.335953	1.88260×10^{-2}	6.93516×10^{-3}
111	1.26827×10^{-4}	4.35349×10^{-2}	1.905398	0.335978	1.82079×10^{-2}	6.81113×10^{-3}
112	1.23362×10^{-4}	4.27980×10^{-2}	1.905398	0.336003	1.82079×10^{-2}	6.69036×10^{-3}
113	1.20016×10^{-4}	4.20796×10^{-2}	1.905836	0.336028	1.75999×10^{-2}	6.57281×10^{-3}
114	1.16784×10^{-4}	4.13792×10^{-2}	1.905836	0.336052	1.75999×10^{-2}	6.45830×10^{-3}
115	1.13661×10^{-4}	4.06961×10^{-2}	1.906275	0.336075	1.70022×10^{-2}	6.34679×10^{-3}
116	1.10644×10^{-4}	4.00299×10^{-2}	1.906275	0.336099	1.70022×10^{-2}	6.23811×10^{-3}
117	1.07727×10^{-4}	3.93798×10^{-2}	1.906714	0.336121	1.64146×10^{-2}	6.13222×10^{-3}
118	1.04907×10^{-4}	3.87455×10^{-2}	1.906714	0.336144	1.64146×10^{-2}	6.02898×10^{-3}
119	1.02180×10^{-4}	3.81264×10^{-2}	1.907153	0.336166	1.58373×10^{-2}	5.92836×10^{-3}
120	9.95415×10^{-5}	3.75221×10^{-2}	1.907153	0.336187	1.58373×10^{-2}	5.83020×10^{-3}
121	9.69890×10^{-5}	3.69320×10^{-2}	1.907153	0.336209	1.58373×10^{-2}	5.73448×10^{-3}
122	9.45187×10^{-5}	3.63557×10^{-2}	1.907593	0.336230	1.52701×10^{-2}	5.64108×10^{-3}
123	9.21276×10^{-5}	3.57928×10^{-2}	1.907593	0.336250	1.52701×10^{-2}	5.54996×10^{-3}
124	8.98125×10^{-5}	3.52429×10^{-2}	1.908032	0.336271	1.47132×10^{-2}	5.46103×10^{-3}
125	8.75705×10^{-5}	3.47056×10^{-2}	1.908032	0.336291	1.47132×10^{-2}	5.37421×10^{-3}
126	8.53988×10^{-5}	3.41805×10^{-2}	1.908471	0.33631	1.41664×10^{-2}	5.28945×10^{-3}
127	8.32948×10^{-5}	3.36672×10^{-2}	1.908471	0.33633	1.41664×10^{-2}	5.20667×10^{-3}
n	$J(n)$ [%]	$Q(n)$ [%]	τ_n^* [s]	$\mathcal{H}_n(\tau_n^*)$ [MPa]	$ERR\tau^*(n)$ [%]	$ERRH^*(n)$ [%]
128	8.12559×10^{-5}	3.31654×10^{-2}	1.908471	0.336349	1.41664×10^{-2}	5.12583×10^{-3}
129	7.92797×10^{-5}	3.26748×10^{-2}	1.908911	0.336368	1.36299×10^{-2}	5.04685×10^{-3}
130	7.73639×10^{-5}	3.21950×10^{-2}	1.908911	0.336386	1.36299×10^{-2}	4.96968×10^{-3}
131	7.55063×10^{-5}	3.17256×10^{-2}	1.90935	0.336404	1.31037×10^{-2}	4.89428×10^{-3}
132	7.37047×10^{-5}	3.12665×10^{-2}	1.90935	0.336422	1.31037×10^{-2}	4.82056×10^{-3}
133	7.19570×10^{-5}	3.08173×10^{-2}	1.90935	0.33644	1.31037×10^{-2}	4.74851×10^{-3}
134	7.02615×10^{-5}	3.03776×10^{-2}	1.90979	0.336457	1.25877×10^{-2}	4.67806×10^{-3}
135	6.86160×10^{-5}	2.99474×10^{-2}	1.90979	0.336474	1.25877×10^{-2}	4.60916×10^{-3}
136	6.70190×10^{-5}	2.95262×10^{-2}	1.90979	0.336491	1.25877×10^{-2}	4.54179×10^{-3}
137	6.54687×10^{-5}	2.91138×10^{-2}	1.91023	0.336508	1.20819×10^{-2}	4.47586×10^{-3}
138	6.39634×10^{-5}	2.87100×10^{-2}	1.91023	0.336524	1.20819×10^{-2}	4.41136×10^{-3}
139	6.25015×10^{-5}	2.83146×10^{-2}	1.91023	0.33654	1.20819×10^{-2}	4.34827×10^{-3}
140	6.10816×10^{-5}	2.79273×10^{-2}	1.91067	0.336556	1.15864×10^{-2}	4.28649×10^{-3}
141	5.97022×10^{-5}	2.75479×10^{-2}	1.91067	0.336572	1.15864×10^{-2}	4.22603×10^{-3}
142	5.83618×10^{-5}	2.71762×10^{-2}	1.91067	0.336587	1.15864×10^{-2}	4.16685×10^{-3}
143	5.70593×10^{-5}	2.68119×10^{-2}	1.91111	0.336603	1.11012×10^{-2}	4.10889×10^{-3}
144	5.57932×10^{-5}	2.64549×10^{-2}	1.91111	0.336618	1.11012×10^{-2}	4.05213×10^{-3}
145	5.45624×10^{-5}	2.61051×10^{-2}	1.91111	0.336633	1.11012×10^{-2}	3.99656×10^{-3}

S3. Results of Numerical Studies for KWW Spectra of Unknown Analytical Models

S3.1. Stretching Exponents $\beta = 0.1$ *and* $\beta = 0.2$

Table S4. For the real relaxation spectrum $\mathcal{H}(\tau)$ (4) of the parameters $G_0 = 0.78$ MPa, $\tau_r = 1.08$ s, $\beta = 0.1$ and $\beta = 0.2$ and its Post–Widder n-the order approximate models $\mathcal{H}_n(\tau)$ (32) the quality indices: the mean relative index $J(n)$ (40) of the discrepancy between the models $\mathcal{H}_n(\tau)$ and $\mathcal{H}_{n+1}(\tau)$, the relaxation times τ_n^* and the spectra models maxima $\mathcal{H}_n(\tau_n^*)$ defined in equation (35).

n	$\beta = 0.1$			$\beta = 0.2$		
	$J(n)$ [%]	τ_n^* [s]	$\mathcal{H}_n(\tau_n^*)$ [MPa]	$J(n)$ [%]	τ_n^* [s]	$\mathcal{H}_n(\tau_n^*)$ [MPa]
1	1.37362×10^{-1}	1.079197	0.028695	5.02662×10^{-1}	1.080191	0.057389
2	1.39155×10^{-2}	1.530396	0.028847	5.36231×10^{-2}	1.558489	0.058666
3	3.34528×10^{-3}	1.701080	0.028885	1.35968×10^{-2}	1.751882	0.058997
4	1.17845×10^{-3}	1.790050	0.028901	4.99243×10^{-3}	1.854835	0.059144
5	5.16581×10^{-4}	1.844953	0.028911	2.25864×10^{-3}	1.917369	0.059226
6	2.61056×10^{-4}	1.883673	0.028917	1.16967×10^{-3}	1.960225	0.059279
7	1.45819×10^{-4}	1.908735	0.028921	6.66126×10^{-4}	1.991159	0.059315
8	8.77401×10^{-5}	1.926840	0.028924	4.07142×10^{-4}	2.013289	0.059342
9	5.59165×10^{-5}	1.945117	0.028926	2.62842×10^{-4}	2.031918	0.059362
10	3.73037×10^{-5}	1.956167	0.028928	1.77256×10^{-4}	2.046945	0.059378
11	2.58321×10^{-5}	1.967279	0.028930	1.23878×10^{-4}	2.058289	0.059391
12	1.84512×10^{-5}	1.974722	0.028931	8.91830×10^{-5}	2.067789	0.059402
13	1.35285×10^{-5}	1.982193	0.028932	6.58384×10^{-5}	2.075422	0.059411
14	1.01437×10^{-5}	1.989693	0.028933	4.96623×10^{-5}	2.083082	0.059419
15	7.75445×10^{-6}	1.993453	0.028933	3.81662×10^{-5}	2.088846	0.059426
16	6.02913×10^{-6}	1.997220	0.028934	2.98144×10^{-5}	2.094626	0.059431
17	4.75811×10^{-6}	2.000995	0.028935	2.36285×10^{-5}	2.100421	0.059437
18	3.80510×10^{-6}	2.004777	0.028935	1.89677×10^{-5}	2.104294	0.059441
19	3.07921×10^{-6}	2.008566	0.028936	1.54020×10^{-5}	2.108174	0.059445
n	$\beta = 0.1$			$\beta = 0.2$		
	$J(n)$ [%]	τ_n^* [s]	$\mathcal{H}_n(\tau_n^*)$ [MPa]	$J(n)$ [%]	τ_n^* [s]	$\mathcal{H}_n(\tau_n^*)$ [MPa]
20	2.51846×10^{-6}	2.012362	0.028936	1.26365×10^{-5}	2.112061	0.059449
21	2.07976×10^{-6}	2.016165	0.028936	1.04650×10^{-5}	2.114007	0.059452
22	1.73256×10^{-6}	2.016165	0.028937	8.74078×10^{-6}	2.117905	0.059455
23	1.45489×10^{-6}	2.019976	0.028937	7.35758×10^{-6}	2.119857	0.059458
24	1.23069×10^{-6}	2.023793	0.028937	6.23756×10^{-6}	2.123765	0.059460
25	1.04806×10^{-6}	2.023793	0.028938	5.32284×10^{-6}	2.125722	0.059462
26	8.98084×10^{-7}	2.027618	0.028938	4.56986×10^{-6}	2.127681	0.059464
27	7.74005×10^{-7}	2.027618	0.028938	3.94548×10^{-6}	2.129642	0.059466
28	6.70633×10^{-7}	2.027618	0.028938	3.42421×10^{-6}	2.131604	0.059468
29	5.83958×10^{-7}	2.031450	0.028938	2.98627×10^{-6}	2.131604	0.059470
30	5.10845×10^{-7}	2.031450	0.028939	2.61618×10^{-6}	2.133568	0.059471
31	4.48825×10^{-7}	2.031450	0.028939	2.30169×10^{-6}	2.135534	0.059473
32	3.95938×10^{-7}	2.035289	0.028939	2.03308×10^{-6}	2.137502	0.059474
33	3.50616×10^{-7}	2.035289	0.028939	1.80254×10^{-6}	2.137502	0.059475
34	3.11598×10^{-7}	2.035289	0.028939	1.60377×10^{-6}	2.139472	0.059476
35	2.77860×10^{-7}	2.035289	0.028939	1.43167×10^{-6}	2.139472	0.059478
36	2.48567×10^{-7}	2.039136	0.028939	1.28205×10^{-6}	2.141443	0.059479
37	2.23035×10^{-7}	2.039136	0.028940	1.15148×10^{-6}	2.141443	0.059480
38	2.00699×10^{-7}	2.039136	0.028940	1.03712×10^{-6}	2.143417	0.059481
39	1.81090×10^{-7}	2.039136	0.028940	9.36608×10^{-7}	2.143417	0.059482
40	1.63818×10^{-7}	2.042990	0.028940	8.47987×10^{-7}	2.145392	0.059482
41	1.48558×10^{-7}	2.042990	0.028940	7.69605×10^{-7}	2.145392	0.059483
42	1.35033×10^{-7}	2.042990	0.028940	7.00074×10^{-7}	2.147369	0.059484
43	1.23014×10^{-7}	2.042990	0.028940	6.38222×10^{-7}	2.147369	0.059485

https://doi.org/10.3390/ma19153256

44	1.12302×10⁻⁷	2.042990	0.028940	5.83054×10⁻⁷	2.147369	0.059485
45	1.02732×10⁻⁷	2.042990	0.028940	5.33720×10⁻⁷	2.149348	0.059486
46	9.41595×10⁻⁸	2.042990	0.028940	4.89496×10⁻⁷	2.149348	0.059487
47	8.64629×10⁻⁸	2.046851	0.028940	4.49759×10⁻⁷	2.149348	0.059487
48	7.95369×10⁻⁸	2.046851	0.028940	4.13974×10⁻⁷	2.151328	0.059488
49	7.32907×10⁻⁸	2.046851	0.028941	3.81679×10⁻⁷	2.151328	0.059488
50	6.76459×10⁻⁸	2.046851	0.028941	3.52473×10⁻⁷	2.151328	0.059489
51	6.25342×10⁻⁸	2.046851	0.028941	3.26008×10⁻⁷	2.151328	0.059490
52	5.78963×10⁻⁸	2.046851	0.028941	3.01981×10⁻⁷	2.153311	0.059490
53	5.36806×10⁻⁸	2.046851	0.028941	2.80128×10⁻⁷	2.153311	0.059491
54	4.98417×10⁻⁸	2.046851	0.028941	2.60216×10⁻⁷	2.153311	0.059491
55	4.63399×10⁻⁸	2.050719	0.028941	2.42042×10⁻⁷	2.153311	0.059491
56	4.31403×10⁻⁸	2.050719	0.028941	2.25428×10⁻⁷	2.155295	0.059492
57	4.02121×10⁻⁸	2.050719	0.028941	2.10216×10⁻⁷	2.155295	0.059492
58	3.75283×10⁻⁸	2.050719	0.028941	1.96265×10⁻⁷	2.155295	0.059493
59	3.50646×10⁻⁸	2.050719	0.028941	1.83452×10⁻⁷	2.155295	0.059493
60	3.27999×10⁻⁸	2.050719	0.028941	1.71669×10⁻⁷	2.155295	0.059493
61	3.07150×10⁻⁸	2.050719	0.028941	1.60816×10⁻⁷	2.157281	0.059494
62	2.87933×10⁻⁸	2.050719	0.028941	1.50808×10⁻⁷	2.157281	0.059494
63	2.70195×10⁻⁸	2.050719	0.028941	1.41566×10⁻⁷	2.157281	0.059494
64	2.53802×10⁻⁸	2.050719	0.028941	1.33022×10⁻⁷	2.157281	0.059495
65	2.38634×10⁻⁸	2.050719	0.028941	1.25113×10⁻⁷	2.157281	0.059495
66	2.24582×10⁻⁸	2.050719	0.028941	1.17782×10⁻⁷	2.157281	0.059495
67	2.11549×10⁻⁸	2.050719	0.028941	1.10981×10⁻⁷	2.159269	0.059496
68	1.99448×10⁻⁸	2.054595	0.028941	1.04664×10⁻⁷	2.159269	0.059496
69	1.88201×10⁻⁸	2.054595	0.028941	9.87903×10⁻⁸	2.159269	0.059496
n		$\beta = 0.1$			$\beta = 0.2$	
	$J(n)$ [%]	τ_n^* [s]	$\mathcal{H}_n(\tau_n^*)$ [MPa]	$J(n)$ [%]	τ_n^* [s]	$\mathcal{H}_n(\tau_n^*)$ [MPa]
70	1.77735×10⁻⁸	2.054595	0.028941	9.33227×10⁻⁸	2.159269	0.059497
71	1.67986×10⁻⁸	2.054595	0.028942	8.82282×10⁻⁸	2.159269	0.059497
72	1.58897×10⁻⁸	2.054595	0.028942	8.34766×10⁻⁸	2.159269	0.059497
73	1.50414×10⁻⁸	2.054595	0.028942	7.90407×10⁻⁸	2.159269	0.059497
74	1.42490×10⁻⁸	2.054595	0.028942	7.48955×10⁻⁸	2.161258	0.059498
75	1.35081×10⁻⁸	2.054595	0.028942	7.10186×10⁻⁸	2.161258	0.059498
76	1.28148×10⁻⁸	2.054595	0.028942	6.73893×10⁻⁸	2.161258	0.059498
77	1.21653×10⁻⁸	2.054595	0.028942	6.39890×10⁻⁸	2.161258	0.059498
78	1.15565×10⁻⁸	2.054595	0.028942	6.08004×10⁻⁸	2.161258	0.059498
79	1.09853×10⁻⁸	2.054595	0.028942	5.78080×10⁻⁸	2.161258	0.059499
80	1.04489×10⁻⁸	2.054595	0.028942	5.49974×10⁻⁸	2.161258	0.059499
81	9.94494×10⁻⁹	2.054595	0.028942	5.23556×10⁻⁸	2.161258	0.059499
82	9.47096×10⁻⁹	2.054595	0.028942	4.98706×10⁻⁸	2.161258	0.059499
83	9.02488×10⁻⁹	2.054595	0.028942	4.75312×10⁻⁸	2.163250	0.059500
84	8.60476×10⁻⁹	2.054595	0.028942	4.53275×10⁻⁸	2.163250	0.059500
85	8.20880×10⁻⁹	2.054595	0.028942	4.32500×10⁻⁸	2.163250	0.059500
86	7.83536×10⁻⁹	2.054595	0.028942	4.12901×10⁻⁸	2.163250	0.059500
87	7.48291×10⁻⁹	2.054595	0.028942	3.94401×10⁻⁸	2.163250	0.059500
88	7.15006×10⁻⁹	2.058478	0.028942	3.76925×10⁻⁸	2.163250	0.059500
89	6.83552×10⁻⁹	2.058478	0.028942	3.60406×10⁻⁸	2.163250	0.059501
90	6.53808×10⁻⁹	2.058478	0.028942	3.44783×10⁻⁸	2.163250	0.059501
91	6.25665×10⁻⁹	2.058478	0.028942	3.29997×10⁻⁸	2.163250	0.059501
92	5.99019×10⁻⁹	2.058478	0.028942	3.15995×10⁻⁸	2.163250	0.059501
93	5.73778×10⁻⁹	2.058478	0.028942	3.02728×10⁻⁸	2.163250	0.059501

94	5.49852×10^{-9}	2.058478	0.028942	2.90150×10^{-8}	2.163250	0.059501
95	5.27160×10^{-9}	2.058478	0.028942	2.78219×10^{-8}	2.165243	0.059502
96	5.05626×10^{-9}	2.058478	0.028942	2.66894×10^{-8}	2.165243	0.059502
97	4.85181×10^{-9}	2.058478	0.028942	2.56140×10^{-8}	2.165243	0.059502
98	4.65760×10^{-9}	2.058478	0.028942	2.45922×10^{-8}	2.165243	0.059502
99	4.47300×10^{-9}	2.058478	0.028942	2.36209×10^{-8}	2.165243	0.059502
100	4.29746×10^{-9}	2.058478	0.028942	2.26971×10^{-8}	2.165243	0.059502
⋮	⋮	⋮	⋮	⋮	⋮	⋮
140	1.12393×10^{-9}	2.062369	0.028943	5.95956×10^{-9}	2.169236	0.059506
141	1.09247×10^{-9}	2.062369	0.028943	5.79317×10^{-9}	2.169236	0.059506
142	1.06211×10^{-9}	2.062369	0.028943	5.63255×10^{-9}	2.169236	0.059506
143	1.03279×10^{-9}	2.062369	0.028943	5.47746×10^{-9}	2.169236	0.059506
144	1.00448×10^{-9}	2.062369	0.028943	5.32768×10^{-9}	2.169236	0.059506
145	9.77134×10^{-10}	2.062369	0.028943	5.18297×10^{-9}	2.169236	0.059506

https://doi.org/10.3390/ma19153256

Table S5. The processes of determining the approximate model $\mathcal{H}_n(\tau)$ of the real relaxation spectrum $\mathcal{H}(\tau)$ (4) with the parameters G_0 = 0.78 MPa, β = 0.1, and τ_r = 1.08 s by using the Algorithm for pre-assumed accuracies ε of the successive models $\mathcal{H}_n(\tau)$ and $\mathcal{H}_{n+1}(\tau)$ discrepancy; n - order of the Post–Widder approximation, $J(n)$ (40) - mean relative index of $\mathcal{H}_n(\tau)$ and $\mathcal{H}_{n+1}(\tau)$ discrepancy.

$\varepsilon > 2.59 \times 10^{-5}$ %		$\varepsilon = 10^{-5}$%		$\varepsilon = 5 \times 10^{-6}$%		$\varepsilon = 10^{-6}$%	
n	$J(n)$ [%]	n	$J(n)$ [%]	n	$J(n)$ [%]	n	$J(n)$ [%]
1	0.1373622	1	0.1373622	1	0.1373622	1	0.1373622
11	2.58321×10^{-5}	11	2.58321×10^{-5}	11	2.58321×10^{-5}	11	2.58321×10^{-5}
		14	1.01437×10^{-5}	16	6.02913×10^{-6}	21	2.07976×10^{-6}
		15	7.75445×10^{-6}	17	4.75811×10^{-6}	23	1.45489×10^{-6}
						25	1.04806×10^{-6}
						26	8.98084×10^{-7}

Table S6. The processes of determining the approximate model $\mathcal{H}_n(\tau)$ of the real relaxation spectrum $\mathcal{H}(\tau)$ (4) with the parameters G_0 = 0.78 MPa, τ_r = 1.08 s, and β = 0.2 by using the Algorithm for pre-assumed accuracies ε of the successive models $\mathcal{H}_n(\tau)$ and $\mathcal{H}_{n+1}(\tau)$ discrepancy; n - order of the Post–Widder approximation, $J(n)$ (40)- mean relative index of $\mathcal{H}_n(\tau)$ and $\mathcal{H}_{n+1}(\tau)$ discrepancy.

$\varepsilon > 1.24 \times 10^{-4}$ %		$\varepsilon = 10^{-5}$%		$\varepsilon = 5 \times 10^{-6}$%		$\varepsilon = 10^{-6}$%	
n	$J(n)$ [%]	n	$J(n)$ [%]	n	$J(n)$ [%]	n	$J(n)$ [%]
1	0.502662	1	0.502662	1	0.502662	1	0.502662
11	1.23878×10^{-4}	11	1.23878×10^{-4}	11	1.23878×10^{-4}	11	1.23878×10^{-4}
		14	4.96623×10^{-5}	21	1.04650×10^{-5}	21	1.04650×10^{-5}
		15	3.81662×10^{-5}	23	7.35758×10^{-6}	31	2.30169×10^{-6}
				25	5.32284×10^{-6}	33	1.80254×10^{-6}
				26	4.56986×10^{-6}	35	1.43167×10^{-6}
						37	1.15148×10^{-6}
						38	1.03712×10^{-6}
						39	9.36608×10^{-7}

S3.2. Stretching exponents $\beta = 0.45$ and $\beta = 0.55$

Table S7. For the real relaxation spectrum $\mathcal{H}(\tau)$ (4) of the parameters G_0 = 0.78 MPa, τ_r = 1.08 s, β = 0.45, and β = 0.55 and its Post–Widder n-the order approximate models $\mathcal{H}_n(\tau)$ (32) the quality indices: the mean relative index $J(n)$ (40) of the models $\mathcal{H}_n(\tau)$ and $\mathcal{H}_{n+1}(\tau)$ discrepancy, the relaxation times τ_n^* and the spectra models maxima $\mathcal{H}_n(\tau_n^*)$ defined in equation (35).

n	$\beta = 0.45$			$\beta = 0.55$		
	$J(n)$ [%]	τ_n^* [s]	$\mathcal{H}_n(\tau_n^*)$ [MPa]	$J(n)$ [%]	τ_n^* [s]	$\mathcal{H}_n(\tau_n^*)$ [MPa]
1	5.787156	1.079803	0.129126	8.239754	1.080037	0.157820
2	1.630475	1.452269	0.144058	2.026983	1.373867	0.184564
3	0.878501	1.645918	0.149524	1.109752	1.536669	0.196101
4	0.557280	1.762418	0.152264	0.703607	1.641037	0.202466
5	0.381372	1.839286	0.153886	0.422585	1.713997	0.206464
6	0.273798	1.894216	0.154950	0.272224	1.767698	0.209191
7	0.203503	1.934683	0.155700	0.184432	1.809076	0.211162
8	0.155380	1.965395	0.156255	0.130161	1.841170	0.212650
9	0.121245	1.990811	0.156682	9.47968×10^{-2}	1.867484	0.213810
10	9.63350×10^{-2}	2.010713	0.157021	7.08681×10^{-2}	1.888923	0.214740

n	$\beta = 0.45$			$\beta = 0.55$		
	$J(n)$ [%]	τ_n^* [s]	$\mathcal{H}_n(\tau_n^*)$ [MPa]	$J(n)$ [%]	τ_n^* [s]	$\mathcal{H}_n(\tau_n^*)$ [MPa]
11	7.77256×10^{-2}	2.027450	0.157296	5.41339×10^{-2}	1.906487	0.215501
12	6.35448×10^{-2}	2.040940	0.157524	4.21403×10^{-2}	1.921840	0.216134
13	5.25534×10^{-2}	2.052817	0.157716	3.33417×10^{-2}	1.934928	0.216670
14	4.39069×10^{-2}	2.063052	0.157880	2.67706×10^{-2}	1.946303	0.217129
15	3.70159×10^{-2}	2.071621	0.158021	2.17550×10^{-2}	1.956538	0.217526
16	3.14601×10^{-2}	2.080225	0.158144	1.78854×10^{-2}	1.965007	0.217873
17	2.69341×10^{-2}	2.087134	0.158252	1.48361×10^{-2}	1.972903	0.218180
18	2.32125×10^{-2}	2.093198	0.158348	1.24270×10^{-2}	1.980221	0.218451
19	2.01263×10^{-2}	2.098410	0.158434	1.04829×10^{-2}	1.986339	0.218694
20	1.75474×10^{-2}	2.103635	0.158511	8.91361×10^{-3}	1.991862	0.218913
21	1.53770×10^{-2}	2.107999	0.158580	7.62866×10^{-3}	1.997400	0.219110
22	1.35386×10^{-2}	2.112372	0.158643	6.56995×10^{-3}	2.001718	0.219289
23	1.19720×10^{-2}	2.115877	0.158700	5.69252×10^{-3}	2.006046	0.219453
24	1.06297×10^{-2}	2.119388	0.158753	4.95502×10^{-3}	2.010382	0.219603
25	9.47360×10^{-3}	2.122905	0.158801	4.33767×10^{-3}	2.014107	0.219741
26	8.47310×10^{-3}	2.125546	0.158845	3.81341×10^{-3}	2.017216	0.219868
27	7.60337×10^{-3}	2.128191	0.158886	3.36628×10^{-3}	2.020330	0.219985
28	6.84414×10^{-3}	2.130839	0.158924	2.98335×10^{-3}	2.023449	0.220094
29	6.17877×10^{-3}	2.133490	0.158960	2.65412×10^{-3}	2.025948	0.220196
30	5.59348×10^{-3}	2.135259	0.158993	2.36682×10^{-3}	2.028449	0.220291
31	5.07684×10^{-3}	2.137916	0.159024	2.12107×10^{-3}	2.030954	0.220379
32	4.61930×10^{-3}	2.139689	0.159053	1.90493×10^{-3}	2.033462	0.220462
33	4.21283×10^{-3}	2.141463	0.159080	1.71666×10^{-3}	2.035345	0.220540
34	3.85066×10^{-3}	2.143239	0.159105	1.55001×10^{-3}	2.037858	0.220613
35	3.52705×10^{-3}	2.145016	0.159129	1.40409×10^{-3}	2.039745	0.220682
36	3.23714×10^{-3}	2.146795	0.159152	1.27417×10^{-3}	2.041004	0.220748
37	2.97675×10^{-3}	2.147685	0.159173	1.15985×10^{-3}	2.042894	0.220809
38	2.74231×10^{-3}	2.149466	0.159194	1.05752×10^{-3}	2.044785	0.220868
39	2.53075×10^{-3}	2.150357	0.159213	9.67093×10^{-4}	2.046047	0.220923
40	2.33942×10^{-3}	2.152141	0.159231	8.85730×10^{-4}	2.047310	0.220976
41	2.16601×10^{-3}	2.153033	0.159249	8.12384×10^{-4}	2.049206	0.221026
42	2.00854×10^{-3}	2.153925	0.159265	7.47213×10^{-4}	2.050471	0.221073
43	1.86525×10^{-3}	2.154818	0.159281	6.88192×10^{-4}	2.051736	0.221119
44	1.73464×10^{-3}	2.156605	0.159296	6.34648×10^{-4}	2.053002	0.221162
45	1.61536×10^{-3}	2.157499	0.159310	5.86846×10^{-4}	2.053636	0.221203
46	1.50626×10^{-3}	2.158394	0.159324	5.42505×10^{-4}	2.054903	0.221243
47	1.40628×10^{-3}	2.159288	0.159337	5.03561×10^{-4}	2.056172	0.221281
48	1.31454×10^{-3}	2.160184	0.159350	4.67251×10^{-4}	2.056806	0.221317
49	1.23022×10^{-3}	2.161079	0.159362	4.34025×10^{-4}	2.058076	0.221352
50	1.15260×10^{-3}	2.161975	0.159373	4.04178×10^{-4}	2.058711	0.221385
51	1.08106×10^{-3}	2.161975	0.159384	3.76764×10^{-4}	2.059981	0.221417
52	1.01503×10^{-3}	2.162871	0.159395	3.51552×10^{-4}	2.060617	0.221448
53	9.53994×10^{-4}	2.163768	0.159405	3.28337×10^{-4}	2.061253	0.221478
54	8.97514×10^{-4}	2.164665	0.159415	3.06935×10^{-4}	2.062525	0.221506
55	8.45182×10^{-4}	2.165562	0.159425	2.87615×10^{-4}	2.063161	0.221534
56	7.96635×10^{-4}	2.165562	0.159434	2.69744×10^{-4}	2.063798	0.221560
57	7.51547×10^{-4}	2.166460	0.159443	2.52812×10^{-4}	2.064435	0.221586
58	7.09625×10^{-4}	2.167358	0.159451	2.37494×10^{-4}	2.065072	0.221611
59	6.70604×10^{-4}	2.167358	0.159460	2.23280×10^{-4}	2.065709	0.221634
60	6.34246×10^{-4}	2.168257	0.159468	2.10399×10^{-4}	2.066346	0.221657

https://doi.org/10.3390/ma19153256

n	$\beta = 0.45$			$\beta = 0.55$		
	$J(n)$ [%]	τ_n^* [s]	$\mathcal{H}_n(\tau_n^*)$ [MPa]	$J(n)$ [%]	τ_n^* [s]	$\mathcal{H}_n(\tau_n^*)$ [MPa]
61	6.00334×10^{-4}	2.168257	0.159475	1.98106×10^{-4}	2.066984	0.221680
62	5.68671×10^{-4}	2.169156	0.159483	1.86666×10^{-4}	2.067622	0.221701
63	5.39081×10^{-4}	2.170055	0.159490	1.76281×10^{-4}	2.068260	0.221722
64	5.11402×10^{-4}	2.170055	0.159497	1.66588×10^{-4}	2.068898	0.221742
65	4.85486×10^{-4}	2.170954	0.159504	1.57291×10^{-4}	2.069536	0.221762
66	4.61201×10^{-4}	2.170954	0.159510	1.48839×10^{-4}	2.070175	0.221781
67	4.38423×10^{-4}	2.171854	0.159517	1.40930×10^{-4}	2.070813	0.221799
68	4.17042×10^{-4}	2.171854	0.159523	1.33524×10^{-4}	2.070813	0.221817
69	3.96955×10^{-4}	2.172755	0.159529	1.26584×10^{-4}	2.071452	0.221835
70	3.78069×10^{-4}	2.172755	0.159535	1.20075×10^{-4}	2.072091	0.221852
71	3.60298×10^{-4}	2.172755	0.159541	1.13966×10^{-4}	2.072731	0.221868
72	3.43564×10^{-4}	2.173656	0.159546	1.08397×10^{-4}	2.072731	0.221884
73	3.27796×10^{-4}	2.173656	0.159551	1.02996×10^{-4}	2.073370	0.221899
74	3.12926×10^{-4}	2.174557	0.159557	9.79161×10^{-5}	2.074010	0.221914
75	2.98893×10^{-4}	2.174557	0.159562	9.32817×10^{-5}	2.074010	0.221929
76	2.85642×10^{-4}	2.174557	0.159567	8.87721×10^{-5}	2.074650	0.221943
77	2.73120×10^{-4}	2.175458	0.159571	8.46560×10^{-5}	2.075290	0.221957
78	2.61280×10^{-4}	2.175458	0.159576	8.07703×10^{-5}	2.075290	0.221971
79	2.50077×10^{-4}	2.176360	0.159581	7.69775×10^{-5}	2.075931	0.221984
80	2.39471×10^{-4}	2.176360	0.159585	7.35135×10^{-5}	2.075931	0.221997
81	2.29424×10^{-4}	2.176360	0.159590	7.02374×10^{-5}	2.076571	0.222010
82	2.19900×10^{-4}	2.177262	0.159594	6.71373×10^{-5}	2.077212	0.222022
83	2.10867×10^{-4}	2.177262	0.159598	6.42020×10^{-5}	2.077212	0.222034
84	2.02295×10^{-4}	2.177262	0.159602	6.14214×10^{-5}	2.077853	0.222045
85	1.94156×10^{-4}	2.178165	0.159606	5.87858×10^{-5}	2.077853	0.222057
86	1.86423×10^{-4}	2.178165	0.159610	5.62864×10^{-5}	2.078494	0.222068
87	1.79073×10^{-4}	2.178165	0.159613	5.39149×10^{-5}	2.078494	0.222079
88	1.72084×10^{-4}	2.178165	0.159617	5.16636×10^{-5}	2.079135	0.222089
89	1.65433×10^{-4}	2.179068	0.159621	4.95254×10^{-5}	2.079135	0.222100
90	1.59101×10^{-4}	2.179068	0.159624	4.74936×10^{-5}	2.079135	0.222110
91	1.53070×10^{-4}	2.179068	0.159628	4.56356×10^{-5}	2.079777	0.222120
92	1.47324×10^{-4}	2.179068	0.159631	4.37956×10^{-5}	2.079777	0.222130
93	1.41846×10^{-4}	2.179971	0.159634	4.20448×10^{-5}	2.080418	0.222139
94	1.36620×10^{-4}	2.179971	0.159638	4.04434×10^{-5}	2.080418	0.222148
95	1.31634×10^{-4}	2.179971	0.159641	3.88535×10^{-5}	2.081060	0.222158
96	1.26874×10^{-4}	2.179971	0.159644	3.73992×10^{-5}	2.081060	0.222166
97	1.22328×10^{-4}	2.180875	0.159647	3.59529×10^{-5}	2.081060	0.222175
98	1.17984×10^{-4}	2.180875	0.159650	3.45738×10^{-5}	2.081702	0.222184
99	1.13832×10^{-4}	2.180875	0.159653	3.33123×10^{-5}	2.081702	0.222192
100	1.09862×10^{-4}	2.180875	0.159655	3.21069×10^{-5}	2.081702	0.222200
⋮	⋮	⋮	⋮	⋮	⋮	⋮
140	3.28118×10^{-5}	2.187211	0.159736	8.99585×10^{-6}	2.088779	0.222432
141	3.19713×10^{-5}	2.187211	0.159737	8.76648×10^{-6}	2.088779	0.222436
142	3.11576×10^{-5}	2.187211	0.159738	8.52999×10^{-6}	2.088779	0.222440
143	3.03697×10^{-5}	2.187211	0.159740	8.30124×10^{-6}	2.088779	0.222444
144	2.96066×10^{-5}	2.187211	0.159741	8.07992×10^{-6}	2.089423	0.222448
145	2.88673×10^{-5}	2.187211	0.159742	7.87906×10^{-6}	2.089423	0.222452

Table S8. The processes of determining the approximate model $\mathcal{H}_n(\tau)$ of the real relaxation spectrum $\mathcal{H}(\tau)$ (4) with the parameters $G_0 = 0.78$ MPa, $\tau_r = 1.08$ s, and $\beta = 0.45$ by using the Algorithm for pre-assumed accuracies ε of the successive models $\mathcal{H}_n(\tau)$ and $\mathcal{H}_{n+1}(\tau)$ discrepancy; n - order of the Post–Widder approximation, $J(n)$ (40) - mean relative index of s $\mathcal{H}_n(\tau)$ and $\mathcal{H}_{n+1}(\tau)$ discrepancy.

$\varepsilon = 10^{-2}\%$		$\varepsilon = 5\times10^{-3}\%$		$\varepsilon = 10^{-3}\%$		$\varepsilon = 5\times10^{-4}\%$	
n	$J(n)$ [%]	n	$J(n)$ [%]	n	$J(n)$ [%]	n	$J(n)$ [%]
1	5.787156	1	5.787156	1	5.787156	1	5.787156
11	7.77256×10^{-2}	11	7.77256×10^{-2}	11	7.77256×10^{-2}	11	7.77256×10^{-2}
16	3.14601×10^{-2}	21	1.53770×10^{-2}	21	1.53770×10^{-2}	21	1.53770×10^{-2}
19	2.01263×10^{-2}	24	1.06297×10^{-2}	31	5.07684×10^{-3}	31	5.07684×10^{-3}
21	1.53770×10^{-2}	26	8.47310×10^{-3}	36	3.23714×10^{-3}	41	2.16601×10^{-3}
23	1.19720×10^{-2}	28	6.84414×10^{-3}	39	2.53075×10^{-3}	44	1.73464×10^{-3}
24	1.06297×10^{-2}	30	5.59348×10^{-3}	42	2.00854×10^{-3}	47	1.40628×10^{-3}
25	9.47360×10^{-3}	31	5.07684×10^{-3}	44	1.73464×10^{-3}	50	1.15260×10^{-3}
		32	4.61930×10^{-3}	46	1.50626×10^{-3}	52	1.01503×10^{-3}
				48	1.31454×10^{-3}	54	8.97514×10^{-4}
				50	1.15260×10^{-3}	56	7.96635×10^{-4}
				51	1.08106×10^{-3}	58	7.09625×10^{-4}
				52	1.01503×10^{-3}	60	6.34246×10^{-4}
				53	9.53994×10^{-4}	62	5.68671×10^{-4}
						63	5.39081×10^{-4}
						64	5.11402×10^{-4}
						65	4.85486×10^{-4}

Table S9. The processes of determining the approximate model $\mathcal{H}_n(\tau)$ of the real relaxation spectrum $\mathcal{H}(\tau)$ (4) with the parameters $G_0 = 0.78$ MPa, $\tau_r = 1.08$ s, and $\beta = 0.55$ by using the Algorithm for pre-assumed accuracies ε of the successive models $\mathcal{H}_n(\tau)$ and $\mathcal{H}_{n+1}(\tau)$ discrepancy; n - order of the Post–Widder approximation, $J(n)$ (40) - mean relative index of $\mathcal{H}_n(\tau)$ and $\mathcal{H}_{n+1}(\tau)$ discrepancy.

$\varepsilon = 10^{-2}\%$		$\varepsilon = 5\times10^{-3}\%$		$\varepsilon = 10^{-3}\%$		$\varepsilon = 5\times10^{-4}\%$		$\varepsilon = 10^{-4}\%$	
n	$J(n)$ [%]	n	$J(n)$ [%]	n	$J(n)$ [%]	n	$J(n)$ [%]	n	$J(n)$ [%]
1	8.239754	1	8.239754	1	8.239754	1	8.239754	1	8.239754
11	5.41339×10^{-2}	11	5.41339×10^{-2}	11	5.41339×10^{-2}	11	5.41339×10^{-2}	11	5.41339×10^{-2}
16	1.78854×10^{-2}	21	7.62866×10^{-3}	21	7.62866×10^{-3}	21	7.62866×10^{-3}	21	7.62866×10^{-3}
18	1.24270×10^{-2}	23	5.69252×10^{-3}	26	3.81341×10^{-3}	31	2.12107×10^{-3}	31	2.12107×10^{-3}
19	1.04829×10^{-2}	24	4.95502×10^{-3}	29	2.65412×10^{-3}	34	1.55001×10^{-3}	41	8.12384×10^{-4}
20	8.91361×10^{-3}			32	1.90493×10^{-3}	37	1.15985×10^{-3}	46	5.42505×10^{-4}
				34	1.55001×10^{-3}	39	9.67093×10^{-4}	51	3.76764×10^{-4}
				36	1.27417×10^{-3}	41	8.12384×10^{-4}	54	3.06935×10^{-4}
				38	1.05752×10^{-3}	43	6.88192×10^{-4}	57	2.52812×10^{-4}
				39	9.67093×10^{-4}	45	5.86846×10^{-4}	60	2.10399×10^{-4}
						46	5.42505×10^{-4}	62	1.86666×10^{-4}
						47	5.03561×10^{-4}	64	1.66588×10^{-4}
						48	4.67251×10^{-4}	66	1.48839×10^{-4}
								68	1.33524×10^{-4}
								70	1.20075×10^{-4}
								71	1.13966×10^{-4}
								72	1.08397×10^{-4}
								73	1.02996×10^{-4}
								74	9.79161×10^{-5}

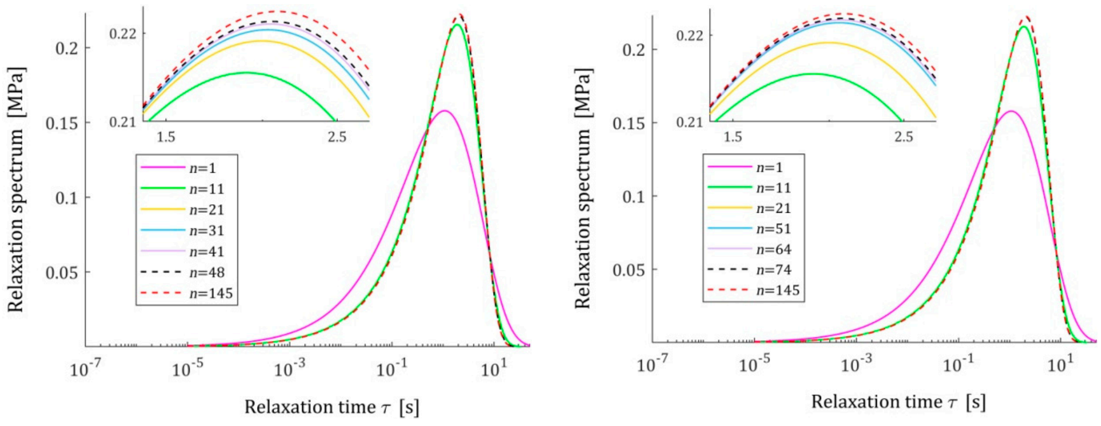

(**a**) (**b**)

Figure S1. For the KWW spectrum $\mathcal{H}(\tau)$ (4) of the parameters of the parameters $G_0 = 0.78$ MPa, $\beta = 0.55$, and $\tau_r = 1.08$ s, the approximate models $\mathcal{H}_n(\tau)$ (32) of the order n iterated by the Algorithm for the accuracies ε of the successive models $\mathcal{H}_n(\tau)$ and $\mathcal{H}_{n+1}(\tau)$ discrepancy; (**a**) $\varepsilon = 5 \times 10^{-4}\%$ and (**b**) $\varepsilon = 10^{-4}\%$.

S3.3. Stretching Exponents $\beta = 0.75$ *and* $\beta = 0.85$

Table S10. For the real relaxation spectrum $\mathcal{H}(\tau)$ (4) of the parameters $G_0 = 0.78$ MPa, $\tau_r = 1.08$ s, $\beta = 0.75$, and $\beta = 0.85$ and its Post–Widder n-the order approximate models $\mathcal{H}_n(\tau)$ (32) the quality indices: the mean relative index $J(n)$ (40) of the discrepancy between the models $\mathcal{H}_n(\tau)$ and $\mathcal{H}_{n+1}(\tau)$, the relaxation times τ_n^* and the spectra models maxima $\mathcal{H}_n(\tau_n^*)$ defined in equation (35).

n	$\beta = 0.75$			$\beta = 0.85$		
	$J(n)$ [%]	τ_n^* [s]	$\mathcal{H}_n(\tau_n^*)$ [MPa]	$J(n)$ [%]	τ_n^* [s]	$\mathcal{H}_n(\tau_n^*)$ [MPa]
1	17.215717	1.080073	0.215209	17.311980	1.080032	0.243904
2	3.531353	1.223864	0.278654	3.904418	1.160158	0.332567
3	1.670146	1.306534	0.315091	1.619045	1.206084	0.389911
4	1.166959	1.363916	0.339723	0.980007	1.237992	0.432388
5	0.814887	1.406718	0.357782	0.570543	1.262345	0.46599
6	0.548236	1.440554	0.371701	0.367387	1.281853	0.493644
7	0.388319	1.468209	0.382805	0.253417	1.298071	0.51702
8	0.285654	1.491364	0.391891	0.183574	1.311953	0.537168
9	0.216449	1.511287	0.399476	0.138008	1.323971	0.554792
10	0.167936	1.528144	0.405907	0.106745	1.334438	0.570392
11	0.132982	1.543048	0.411432	8.45380×10^{-2}	1.343874	0.584333
12	0.107080	1.556246	0.416231	6.82193×10^{-2}	1.352255	0.596890
13	8.74832×10^{-2}	1.568004	0.420439	5.59579×10^{-2}	1.359937	0.608279
14	7.23370×10^{-2}	1.57860	0.424158	4.65418×10^{-2}	1.366906	0.618668
15	6.04954×10^{-2}	1.588008	0.427469	3.91481×10^{-2}	1.373342	0.628193
16	5.10775×10^{-2}	1.59684	0.430436	3.32410×10^{-2}	1.379237	0.636965
17	4.35016×10^{-2}	1.604449	0.433109	2.85277×10^{-2}	1.384774	0.645075
18	3.73372×10^{-2}	1.611775	0.43553	2.46470×10^{-2}	1.389949	0.6526
19	3.22628×10^{-2}	1.618493	0.437732	2.14625×10^{-2}	1.394565	0.659604
20	2.80363×10^{-2}	1.624595	0.439744	1.87985×10^{-2}	1.399197	0.666143
21	2.45321×10^{-2}	1.630075	0.441589	1.65628×10^{-2}	1.403263	0.672264
22	2.15649×10^{-2}	1.635249	0.443287	1.46641×10^{-2}	1.407145	0.678006
23	1.90444×10^{-2}	1.640115	0.444855	1.30630×10^{-2}	1.411039	0.683406
24	1.68969×10^{-2}	1.644669	0.446307	1.16816×10^{-2}	1.414357	0.688494
25	1.50620×10^{-2}	1.64891	0.447655	1.04871×10^{-2}	1.417683	0.693298

https://doi.org/10.3390/ma19153256

n	$\beta = 0.75$			$\beta = 0.85$		
	$J(n)$ [%]	τ_n^* [s]	$\mathcal{H}_n(\tau_n^*)$ [MPa]	$J(n)$ [%]	τ_n^* [s]	$\mathcal{H}_n(\tau_n^*)$ [MPa]
26	1.34727×10^{-2}	1.652834	0.44891	9.45211×10^{-3}	1.421016	0.697841
27	1.20928×10^{-2}	1.656767	0.450081	8.54259×10^{-3}	1.423964	0.702145
28	1.08917×10^{-2}	1.660052	0.451177	7.75206×10^{-3}	1.426721	0.706229
29	9.84399×10^{-3}	1.663343	0.452204	7.05458×10^{-3}	1.429483	0.710109
30	8.92816×10^{-3}	1.666641	0.453168	6.43821×10^{-3}	1.432053	0.713801
31	8.10367×10^{-3}	1.669615	0.454075	5.89275×10^{-3}	1.434628	0.717318
32	7.39118×10^{-3}	1.672263	0.454931	5.40187×10^{-3}	1.437008	0.720673
33	6.74619×10^{-3}	1.674915	0.455738	4.96652×10^{-3}	1.439193	0.723877
34	6.17910×10^{-3}	1.677571	0.456502	4.58001×10^{-3}	1.441382	0.72694
35	5.67160×10^{-3}	1.679898	0.457225	4.23040×10^{-3}	1.443575	0.729872
36	5.20915×10^{-3}	1.682229	0.457911	3.91382×10^{-3}	1.44557	0.73268
37	4.80132×10^{-3}	1.68423	0.458562	3.62684×10^{-3}	1.447369	0.735372
38	4.43467×10^{-3}	1.686232	0.459182	3.36642×10^{-3}	1.44937	0.737956
39	4.09849×10^{-3}	1.688237	0.459771	3.13458×10^{-3}	1.451173	0.740439
40	3.79562×10^{-3}	1.690245	0.460333	2.91923×10^{-3}	1.452778	0.742825
41	3.52241×10^{-3}	1.69192	0.460869	2.72325×10^{-3}	1.454586	0.74512
42	3.27066×10^{-3}	1.693932	0.461381	2.54870×10^{-3}	1.456194	0.74733
43	3.04315×10^{-3}	1.69561	0.461871	2.38578×10^{-3}	1.457603	0.74946
44	2.83731×10^{-3}	1.696954	0.46234	2.23362×10^{-3}	1.459215	0.751513
45	2.64674×10^{-3}	1.698636	0.462789	2.09813×10^{-3}	1.460627	0.753494
46	2.47404×10^{-3}	1.699982	0.463219	1.97119×10^{-3}	1.46204	0.755406
47	2.31374×10^{-3}	1.701329	0.463632	1.85221×10^{-3}	1.463455	0.757254
48	2.16826×10^{-3}	1.702678	0.464029	1.74635×10^{-3}	1.464668	0.75904
49	2.03287×10^{-3}	1.704027	0.46441	1.64410×10^{-3}	1.466086	0.760767
50	1.90985×10^{-3}	1.705378	0.464777	1.55318×10^{-3}	1.467301	0.762438
51	1.79506×10^{-3}	1.70673	0.46513	1.46753×10^{-3}	1.468518	0.764056
52	1.69065×10^{-3}	1.707744	0.46547	1.38681×10^{-3}	1.469736	0.765623
53	1.59036×10^{-3}	1.708759	0.465798	1.31293×10^{-3}	1.470752	0.767142
54	1.50157×10^{-3}	1.710114	0.466114	1.24317×10^{-3}	1.471971	0.768615
55	1.41596×10^{-3}	1.71113	0.46642	1.17930×10^{-3}	1.472989	0.770044
56	1.33797×10^{-3}	1.712147	0.466714	1.11887×10^{-3}	1.474006	0.771431
57	1.26476×10^{-3}	1.713165	0.466999	1.06354×10^{-3}	1.475025	0.772778
58	1.19600×10^{-3}	1.714183	0.467275	1.01110×10^{-3}	1.476044	0.774086
59	1.13330×10^{-3}	1.714863	0.467541	9.61371×10^{-4}	1.477064	0.775357
60	1.07427×10^{-3}	1.715882	0.467799	9.15835×10^{-4}	1.477881	0.776593
61	1.01868×10^{-3}	1.716902	0.468049	8.72579×10^{-4}	1.478902	0.777795
62	9.66283×10^{-4}	1.717582	0.46829	8.31476×10^{-4}	1.47972	0.778964
63	9.18473×10^{-4}	1.718263	0.468525	7.92407×10^{-4}	1.480538	0.780102
64	8.73318×10^{-4}	1.719284	0.468753	7.56640×10^{-4}	1.481356	0.78121
65	8.30650×10^{-4}	1.719965	0.468973	7.22582×10^{-4}	1.48238	0.782289
66	7.90313×10^{-4}	1.720647	0.469188	6.91415×10^{-4}	1.482994	0.783341
67	7.53491×10^{-4}	1.721328	0.469396	6.60457×10^{-4}	1.483814	0.784366
68	7.17334×10^{-4}	1.722352	0.469598	6.32132×10^{-4}	1.484634	0.785365
69	6.84324×10^{-4}	1.723034	0.469794	6.05096×10^{-4}	1.485455	0.78634
70	6.53024×10^{-4}	1.723717	0.469985	5.80377×10^{-4}	1.486071	0.78729
71	6.23332×10^{-4}	1.724058	0.470171	5.55687×10^{-4}	1.486892	0.788218
72	5.96228×10^{-4}	1.724741	0.470352	5.33118×10^{-4}	1.487509	0.789123
73	5.69433×10^{-4}	1.725425	0.470528	5.11526×10^{-4}	1.488331	0.790008
74	5.44974×10^{-4}	1.726108	0.470699	4.90866×10^{-4}	1.488948	0.790871
75	5.21705×10^{-4}	1.726792	0.470866	4.72002×10^{-4}	1.489565	0.791715

n	$\beta = 0.75$			$\beta = 0.85$		
	$J(n)$ [%]	τ_n^* [s]	$\mathcal{H}_n(\tau_n^*)$ [MPa]	$J(n)$ [%]	τ_n^* [s]	$\mathcal{H}_n(\tau_n^*)$ [MPa]
76	4.99561×10^{-4}	1.727134	0.471029	4.53039×10^{-4}	1.490183	0.792539
77	4.78479×10^{-4}	1.727819	0.471188	4.35731×10^{-4}	1.4908	0.793345
78	4.58401×10^{-4}	1.728503	0.471342	4.19131×10^{-4}	1.491418	0.794133
79	4.39272×10^{-4}	1.728846	0.471493	4.03208×10^{-4}	1.492037	0.794904
80	4.21824×10^{-4}	1.729531	0.47164	3.87931×10^{-4}	1.492655	0.795657
81	4.04414×10^{-4}	1.729873	0.471784	3.74012×10^{-4}	1.493274	0.796395
82	3.88536×10^{-4}	1.730559	0.471924	3.59916×10^{-4}	1.493893	0.797116
83	3.73369×10^{-4}	1.730901	0.472061	3.47078×10^{-4}	1.494306	0.797823
84	3.58875×10^{-4}	1.731587	0.472195	3.34734×10^{-4}	1.494925	0.798515
85	3.45022×10^{-4}	1.73193	0.472325	3.22863×10^{-4}	1.495545	0.799192
86	3.31775×10^{-4}	1.732273	0.472453	3.11443×10^{-4}	1.495958	0.799855
87	3.19106×10^{-4}	1.73296	0.472578	3.00457×10^{-4}	1.496579	0.800505
88	3.06984×10^{-4}	1.733303	0.4727	2.89885×10^{-4}	1.496992	0.801142
89	2.95949×10^{-4}	1.733646	0.472819	2.80286×10^{-4}	1.497406	0.801767
90	2.84824×10^{-4}	1.73399	0.472936	2.71032×10^{-4}	1.498027	0.802379
91	2.74699×10^{-4}	1.734333	0.47305	2.61569×10^{-4}	1.498441	0.802979
92	2.64478×10^{-4}	1.735021	0.473162	2.52982×10^{-4}	1.498855	0.803567
93	2.55177×10^{-4}	1.735364	0.473272	2.44700×10^{-4}	1.499476	0.804145
94	2.46252×10^{-4}	1.735708	0.473379	2.36711×10^{-4}	1.49989	0.804711
95	2.37685×10^{-4}	1.736052	0.473484	2.29003×10^{-4}	1.500305	0.805267
96	2.29459×10^{-4}	1.736396	0.473587	2.22034×10^{-4}	1.50072	0.805812
97	2.21559×10^{-4}	1.73674	0.473688	2.14843×10^{-4}	1.501134	0.806347
98	2.13969×10^{-4}	1.737084	0.473786	2.07901×10^{-4}	1.501549	0.806873
99	2.06677×10^{-4}	1.737428	0.473883	2.01630×10^{-4}	1.501964	0.807389
100	1.99668×10^{-4}	1.737772	0.473978	1.95565×10^{-4}	1.502379	0.807895
101	1.93310×10^{-4}	1.738116	0.474071	1.89294×10^{-4}	1.502794	0.808393
102	1.86819×10^{-4}	1.738461	0.474162	1.83633×10^{-4}	1.50321	0.808882
103	1.80576×10^{-4}	1.738805	0.474252	1.78155×10^{-4}	1.503625	0.809362
104	1.74917×10^{-4}	1.739149	0.47434	1.72856×10^{-4}	1.504041	0.809834
105	1.69126×10^{-4}	1.739494	0.474426	1.67728×10^{-4}	1.504248	0.810298
106	1.63880×10^{-4}	1.739838	0.474511	1.62766×10^{-4}	1.504664	0.810754
107	1.58823×10^{-4}	1.740183	0.474594	1.58308×10^{-4}	1.50508	0.811203
108	1.53638×10^{-4}	1.740528	0.474675	1.53649×10^{-4}	1.505496	0.811644
109	1.48944×10^{-4}	1.740528	0.474756	1.49138×10^{-4}	1.505704	0.812077
110	1.44416×10^{-4}	1.740873	0.474834	1.45090×10^{-4}	1.50612	0.812503
111	1.40047×10^{-4}	1.741217	0.474911	1.40852×10^{-4}	1.506536	0.812923
112	1.35830×10^{-4}	1.741562	0.474987	1.37051×10^{-4}	1.506744	0.813335
113	1.31760×10^{-4}	1.741907	0.475062	1.33067×10^{-4}	1.507161	0.813741
114	1.27831×10^{-4}	1.741907	0.475135	1.29496×10^{-4}	1.507369	0.814141
115	1.24037×10^{-4}	1.742252	0.475207	1.26030×10^{-4}	1.507786	0.814534
116	1.20372×10^{-4}	1.742598	0.475278	1.22667×10^{-4}	1.507994	0.814921
117	1.16832×10^{-4}	1.742943	0.475348	1.19403×10^{-4}	1.508411	0.815302
118	1.13412×10^{-4}	1.742943	0.475416	1.16234×10^{-4}	1.508619	0.815677
119	1.10107×10^{-4}	1.743288	0.475484	1.13157×10^{-4}	1.509036	0.816046
120	1.07132×10^{-4}	1.743633	0.47555	1.10170×10^{-4}	1.509244	0.81641
121	1.04038×10^{-4}	1.743633	0.475615	1.07268×10^{-4}	1.509662	0.816768
122	1.01046×10^{-4}	1.743979	0.475679	1.04451×10^{-4}	1.50987	0.81712
123	9.83562×10^{-5}	1.744324	0.475742	1.01714×10^{-4}	1.510079	0.817468
124	9.55523×10^{-5}	1.744324	0.475805	9.90552×10^{-5}	1.510496	0.817810
125	9.30330×10^{-5}	1.744473	0.475836	9.66946×10^{-5}	1.510611	0.818048

n	$\beta = 0.75$			$\beta = 0.85$		
	$J(n)$ [%]	τ_n^* [s]	$\mathcal{H}_n(\tau_n^*)$ [MPa]	$J(n)$ [%]	τ_n^* [s]	$\mathcal{H}_n(\tau_n^*)$ [MPa]
⋮	⋮	⋮	⋮	⋮	⋮	⋮
140	9.20315×10^{-5}	1.744682	0.475872	9.59742×10^{-5}	1.510705	0.818244
141	9.04034×10^{-5}	1.745015	0.475926	9.41797×10^{-5}	1.510914	0.818480
142	8.80422×10^{-5}	1.745015	0.475985	9.19484×10^{-5}	1.511331	0.818807
143	8.55744×10^{-5}	1.745361	0.476043	8.95684×10^{-5}	1.51154	0.81913
144	8.33600×10^{-5}	1.745707	0.476101	8.74584×10^{-5}	1.511749	0.819449
145	8.12128×10^{-5}	1.745707	0.476157	8.52053×10^{-5}	1.511958	0.819762

Table S11. The process of determining the approximate model $\mathcal{H}_n(\tau)$ of the real relaxation spectrum $\mathcal{H}(\tau)$ (4) with the parameters $G_0 = 0.78$ MPa, $\beta = 0.75$, and $\tau_r = 1.08$ s by using the Algorithm for pre-assumed accuracies ε of the successive models $\mathcal{H}_n(\tau)$ and $\mathcal{H}_{n+1}(\tau)$ discrepancy; n - order of the Post–Widder approximation, $J(n)$ (40) - mean relative index of $\mathcal{H}_n(\tau)$ and $\mathcal{H}_{n+1}(\tau)$ discrepancy.

$\varepsilon = 10^{-2}\%$		$\varepsilon = 5\times10^{-3}\%$		$\varepsilon = 10^{-3}\%$		$\varepsilon = 5\times10^{-4}\%$		$\varepsilon = 10^{-4}\%$	
n	$J(n)$ [%]	n	$J(n)$ [%]	n	$J(n)$ [%]	n	$J(n)$ [%]	n	$J(n)$ [%]
1	17.215717	1	17.215717	1	17.215717	1	17.215717	1	17.215717
11	0.132982	11	0.132982	11	0.132982	11	0.132982	11	0.132982
21	2.45321×10^{-2}	21	2.45321×10^{-2}	21	2.45321×10^{-2}	21	2.45321×10^{-2}	21	2.45321×10^{-2}
23	1.90444×10^{-2}	24	1.68969×10^{-2}	31	8.10367×10^{-3}	31	8.10367×10^{-3}	31	8.10367×10^{-3}
25	1.50620×10^{-2}	27	1.20928×10^{-2}	36	5.20915×10^{-3}	41	3.52241×10^{-3}	41	3.52241×10^{-3}
27	1.20928×10^{-2}	29	9.84399×10^{-3}	41	3.52241×10^{-3}	46	2.47404×10^{-3}	51	1.79506×10^{-3}
28	1.08917×10^{-2}	31	8.10367×10^{-3}	44	2.83731×10^{-3}	49	2.03287×10^{-3}	61	1.01868×10^{-3}
29	9.84399×10^{-3}	33	6.74619×10^{-3}	47	2.31374×10^{-3}	52	1.69065×10^{-3}	71	6.23332×10^{-4}
		35	5.67160×10^{-3}	49	2.03287×10^{-3}	55	1.41596×10^{-3}	76	4.99561×10^{-4}
		36	5.20915×10^{-3}	51	1.79506×10^{-3}	58	1.19600×10^{-3}	79	4.39272×10^{-4}
		37	4.80132×10^{-3}	53	1.59036×10^{-3}	60	1.07427×10^{-3}	82	3.88536×10^{-4}
				55	1.41596×10^{-3}	62	9.66283×10^{-4}	85	3.45022×10^{-4}
				57	1.26476×10^{-3}	64	8.73318×10^{-4}	88	3.06984×10^{-4}
				59	1.13330×10^{-3}	66	7.90313×10^{-4}	91	2.74699×10^{-4}
				60	1.07427×10^{-3}	68	7.17334×10^{-4}	94	2.46252×10^{-4}
				61	1.01868×10^{-3}	70	6.53024×10^{-4}	96	2.29459×10^{-4}
				62	9.66283×10^{-4}	72	5.96228×10^{-4}	98	2.13969×10^{-4}
						73	5.69433×10^{-4}	100	1.99668×10^{-4}
						74	5.44974×10^{-4}	102	1.86819×10^{-4}
						75	5.21705×10^{-4}	104	1.74917×10^{-4}
						76	4.99561×10^{-4}	106	1.63880×10^{-4}
								108	1.53638×10^{-4}
								110	1.44416×10^{-4}
								112	1.35830×10^{-4}
								114	1.27831×10^{-4}
								116	1.20372×10^{-4}
								117	1.16832×10^{-4}
								118	1.13412×10^{-4}
								119	1.10107×10^{-4}
								120	1.07132×10^{-4}
								121	1.04038×10^{-4}
								122	1.01046×10^{-4}
								123	9.83562×10^{-5}

Table S12. The processes of determining the approximate model $\mathcal{H}_n(\tau)$ of the real relaxation spectrum $\mathcal{H}(\tau)$ (4) with the parameters G_0 = 0.78 MPa, β = 0.85, and τ_r = 1.08 s by using the Algorithm for pre-assumed accuracies ε of the successive models $\mathcal{H}_n(\tau)$ and $\mathcal{H}_{n+1}(\tau)$ discrepancy; n - order of the Post–Widder approximation, $J(n)$ (40) - mean relative index of $\mathcal{H}_n(\tau)$ and $\mathcal{H}_{n+1}(\tau)$ discrepancy.

$\varepsilon = 10^{-2}\%$		$\varepsilon = 5 \times 10^{-3}\%$		$\varepsilon = 10^{-3}\%$		$\varepsilon = 5 \times 10^{-4}\%$		$\varepsilon = 10^{-4}\%$	
n	$J(n)$ [%]	n	$J(n)$ [%]	n	$J(n)$ [%]	n	$J(n)$ [%]	n	$J(n)$ [%]
1	17.311980	1	17.311980	1	17.311980	1	17.311980	1	17.311980
11	8.45380×10^{-2}	11	8.45380×10^{-2}	11	8.45380×10^{-2}	11	8.45380×10^{-2}	11	8.45380×10^{-2}
16	3.32410×10^{-2}	21	1.65628×10^{-2}	21	1.65628×10^{-2}	21	1.65628×10^{-2}	21	1.65628×10^{-2}
19	2.14625×10^{-2}	24	1.16816×10^{-2}	31	5.89275×10^{-3}	31	5.89275×10^{-3}	31	5.89275×10^{-3}
21	1.65628×10^{-2}	26	9.45211×10^{-3}	36	3.91382×10^{-3}	41	2.72325×10^{-3}	41	2.72325×10^{-3}
23	1.30630×10^{-2}	28	7.75206×10^{-3}	39	3.13458×10^{-3}	46	1.97119×10^{-3}	51	1.46753×10^{-3}
25	1.04871×10^{-2}	30	6.43821×10^{-3}	42	2.54870×10^{-3}	49	1.64410×10^{-3}	61	8.72579×10^{-4}
26	9.45211×10^{-3}	32	5.40187×10^{-3}	45	2.09813×10^{-3}	52	1.38681×10^{-3}	66	6.91415×10^{-4}
		33	4.96652×10^{-3}	47	1.85221×10^{-3}	55	1.17930×10^{-3}	71	5.55687×10^{-4}
				49	1.64410×10^{-3}	57	1.06354×10^{-3}	76	4.53039×10^{-4}
				51	1.46753×10^{-3}	59	9.61371×10^{-4}	79	4.03208×10^{-4}
				53	1.31293×10^{-3}	61	8.72579×10^{-4}	82	3.59916×10^{-4}
				55	1.17930×10^{-3}	63	7.92407×10^{-4}	85	3.22863×10^{-4}
				56	1.11887×10^{-3}	65	7.22582×10^{-4}	88	2.89885×10^{-4}
				57	1.06354×10^{-3}	67	6.60457×10^{-4}	91	2.61569×10^{-4}
				58	1.01110×10^{-3}	69	6.05096×10^{-4}	94	2.36711×10^{-4}
				59	9.61371×10^{-4}	70	5.80377×10^{-4}	96	2.22034×10^{-4}
						71	5.55687×10^{-4}	98	2.07901×10^{-4}
						72	5.33118×10^{-4}	100	1.95565×10^{-4}
						73	5.11526×10^{-4}	102	1.83633×10^{-4}
						74	4.90866×10^{-4}	104	1.72856×10^{-4}
								106	1.62766×10^{-4}
								108	1.53649×10^{-4}
								110	1.45090×10^{-4}
								112	1.37051×10^{-4}
								114	1.29496×10^{-4}
								116	1.22667×10^{-4}
								117	1.19403×10^{-4}
								118	1.16234×10^{-4}
								119	1.13157×10^{-4}
								120	1.10170×10^{-4}
								121	1.07268×10^{-4}
								122	1.04451×10^{-4}
								123	1.01714×10^{-4}
								124	9.90552×10^{-5}

https://doi.org/10.3390/ma19153256

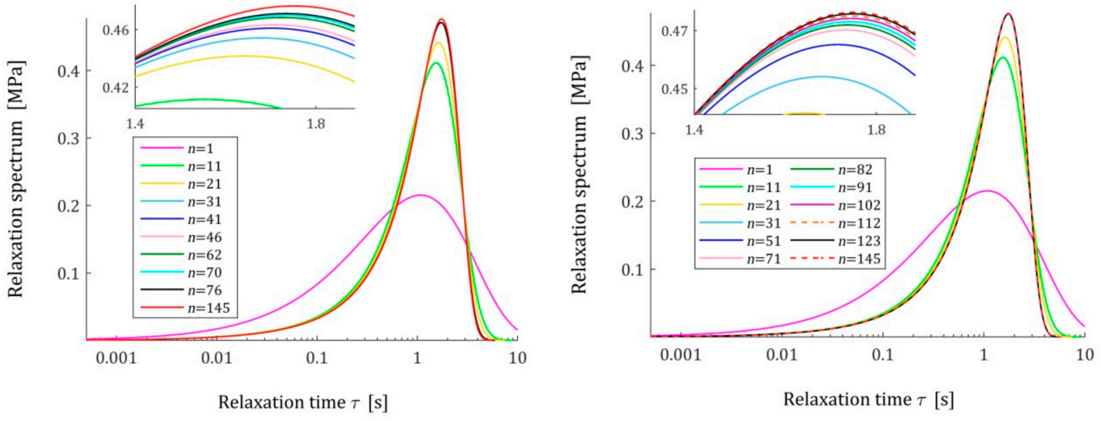

(**a**) (**b**)

Figure S2. For the KWW spectrum $\mathcal{H}(\tau)$ (4) of the parameters $G_0 = 0.78$ MPa, $\beta = 0.75$, and $\tau_r = 1.08$ s, the approximate models $\mathcal{H}_n(\tau)$ (32) of the order n iterated by the Algorithm for the accuracies ε of the successive models $\mathcal{H}_n(\tau)$ and $\mathcal{H}_{n+1}(\tau)$ discrepancy; (**a**) $\varepsilon = 5 \times 10^{-4}\%$ and (**b**) $\varepsilon = 10^{-4}\%$.

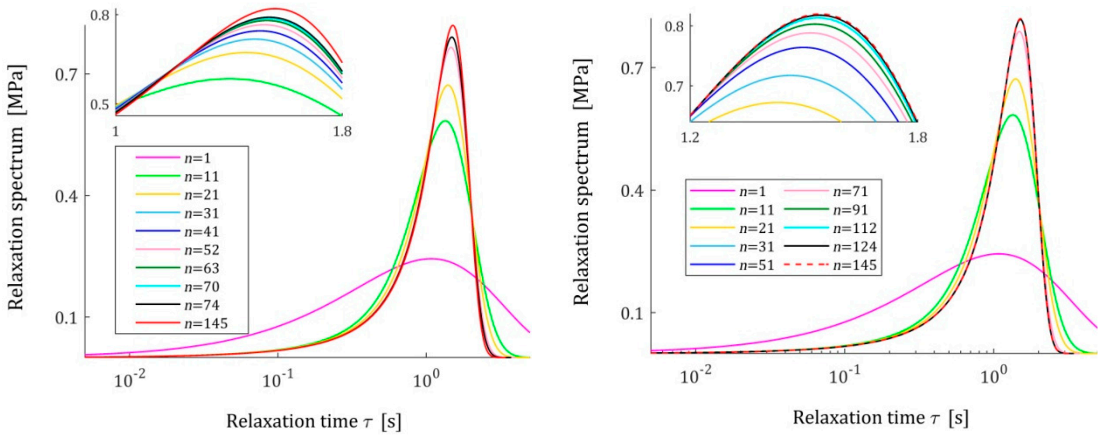

(**a**) (**b**)

Figure S3. For the KWW spectrum $\mathcal{H}(\tau)$ (4) of the parameters $G_0 = 0.78$ MPa, $\beta = 0.85$, and $\tau_r = 1.08$ s, the approximate models $\mathcal{H}_n(\tau)$ (32) of the order n iterated by the Algorithm for the accuracies ε of the successive models $\mathcal{H}_n(\tau)$ and $\mathcal{H}_{n+1}(\tau)$ discrepancy; (**a**) $\varepsilon = 5 \times 10^{-4}\%$ and (**b**) $\varepsilon = 10^{-4}\%$.

S4. Sensitivity of the KWW Spectrum Models to β Perturbations

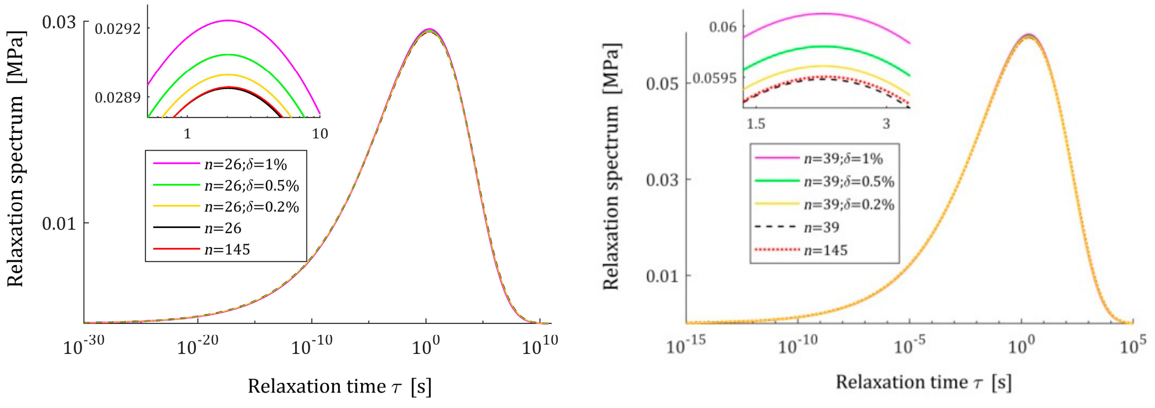

(**a**) (**b**)

Figure S4. The KWW spectrum $\mathcal{H}(\tau)$ (7) (solid red line) of the parameters: $G_0 = 0.78$ MPa, $\tau_r = 1.08$ s and the original $\mathcal{H}_n(\tau)$ (32) and perturbed $\bar{\mathcal{H}}_n(\tau)$ models determined for 0.2%, 0.5% and 1% perturbations of the stretching exponents: (**a**) $\beta = 0.1$, (**b**) $\beta = 0.2$.

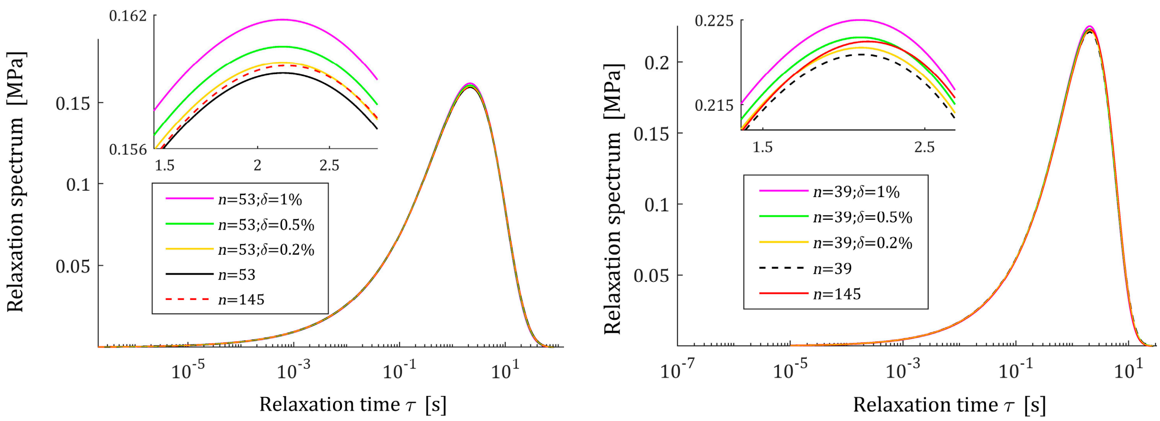

(**a**) (**b**)

Figure S5. The KWW spectrum $\mathcal{H}(\tau)$ (7) (solid red line) of the parameters: $G_0 = 0.78$ MPa, $\tau_r = 1.08$ s and the original $\mathcal{H}_n(\tau)$ (32) and perturbed $\bar{\mathcal{H}}_n(\tau)$ models determined for 0.2%, 0.5% and 1% perturbations of the stretching exponents: (**a**) $\beta = 0.45$, (**b**) $\beta = 0.55$.

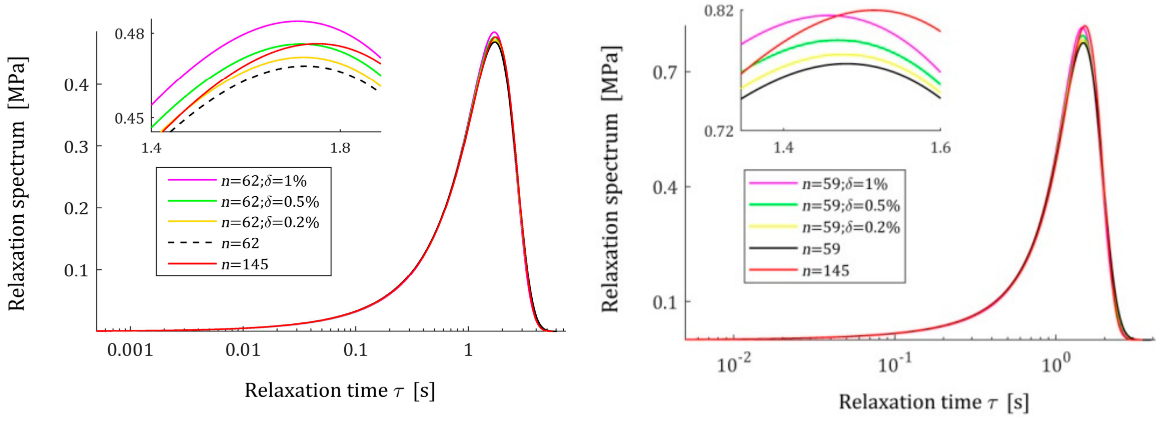

(**a**) (**b**)

Figure S6. The KWW spectrum $\mathcal{H}(\tau)$ (7) (solid red line) of the parameters: $G_0 = 0.78$ MPa, $\tau_r = 1.08$ s and the original $\mathcal{H}_n(\tau)$ (32) and perturbed $\bar{\mathcal{H}}_n(\tau)$ models determined for 0.2%, 0.5% and 1% perturbations of the stretching exponents: (**a**) $\beta = 0.75$, (**b**) $\beta = 0.85$.

https://doi.org/10.3390/ma19153256
